# Melanoma and immunotherapy bridge 2015: Naples, Italy. 1–5 December 2015

**DOI:** 10.1186/s12967-016-0791-2

**Published:** 2016-07-25

**Authors:** Vashisht G. Y. Nanda, Weiyi Peng, Patrick Hwu, Michael A. Davies, Gennaro Ciliberto, Luigi Fattore, Debora Malpicci, Luigi Aurisicchio, Paolo Antonio Ascierto, Carlo M. Croce, Rita Mancini, Stefani Spranger, Thomas F. Gajewski, Yangyang Wang, Soldano Ferrone, Claire Vanpouille-Box, Erik Wennerberg, Karsten A. Pilones, Silvia C. Formenti, Sandra Demaria, Haidong Tang, Yang Wang, Yang-Xin Fu, Reinhard Dummer, Igor Puzanov, Ahmad Tarhini, Joe-Marc Chauvin, Ornella Pagliano, Julien Fourcade, Zhaojun Sun, Hong Wang, Cindy Sanders, John M. Kirkwood, Tseng-hui Timothy Chen, Mark Maurer, Alan J. Korman, Hassane M. Zarour, David F. Stroncek, Veronica Huber, Licia Rivoltini, Magdalena Thurin, Tilman Rau, Alessandro Lugli, Franck Pagès, Jorge Camarero, Arantxa Sancho, Claudio Jommi, Yago Pico de Coaña, Maria Wolodarski, Yuya Yoshimoto, Giusy Gentilcore, Isabel Poschke, Giuseppe V. Masucci, Johan Hansson, Rolf Kiessling, Giosuè Scognamiglio, Francesco Sabbatino, Federica Zito Marino, Anna Maria Anniciello, Monica Cantile, Margherita Cerrone, Stefania Scala, Crescenzo D’alterio, Angela Ianaro, Giuseppe Cirin, Giuseppina Liguori, Gerardo Bott, Paul B. Chapman, Caroline Robert, James Larkin, John B. Haanen, Antoni Ribas, David Hogg, Omid Hamid, Alessandro Testori, Paul Lorigan, Jeffrey A. Sosman, Keith T. Flaherty, Huibin Yue, Shelley Coleman, Ivor Caro, Axel Hauschild, Grant A. McArthur, Mario Sznol, Margaret K. Callahan, Harriet Kluger, Michael A. Postow, RuthAnn Gordan, Neil H. Segal, Naiyer A. Rizvi, Alexander Lesokhin, Michael B. Atkins, Matthew M. Burke, Amanda Ralabate, Angel Rivera, Stephanie A. Kronenberg, Blessing Agunwamba, Mary Ruisi, Christine Horak, Joel Jiang, Jedd Wolchok, Paolo A. Ascierto, Gabriella Liszkay, Michele Maio, Mario Mandalà, Lev Demidov, Daniil Stoyakovskiy, Luc Thomas, Luis de la Cruz-Merino, Victoria Atkinson, Caroline Dutriaux, Claus Garbe, Matthew Wongchenko, Ilsung Chang, Daniel O. Koralek, Isabelle Rooney, Yibing Yan, Brigitte Dréno, Ryan Sullivan, Manish Patel, Stephen Hodi, Rodabe Amaria, Peter Boasberg, Jeffrey Wallin, Xian He, Edward Cha, Nicole Richie, Marcus Ballinger, David C. Smith, Todd M. Bauer, Jeffrey S. Wasser, Jason J. Luke, Ani S. Balmanoukian, David R. Kaufman, Yufan Zhao, Janet Maleski, Lance Leopold, Tara C. Gangadhar, Georgina V. Long, Olivier Michielin, Ari VanderWalde, Robert H. I. Andtbacka, Jonathan Cebon, Eugenio Fernandez, Josep Malvehy, Anthony J. Olszanski, Christine Gause, Lisa Chen, Jeffrey Chou, F. Stephen Hodi, Benjamin Brady, Laurent Mortier, Jessica C. Hassel, Piotr Rutkowski, Catriona McNeil, Ewa Kalinka-Warzocha, Celeste Lebbé, Lars Ny, Matias Chacon, Paola Queirolo, Carmen Loquai, Parneet Cheema, Alfonso Berrocal, Karmele Mujika Eizmendi, Gil Bar-Sela, Christine Horak, Helene Hardy, Jeffrey S. Weber, Jean-Jacques Grob, Ivan Marquez-Rodas, Henrik Schmidt, Karen Briscoe, Jean-François Baurain, Jedd D. Wolchok, Rosamaria Pinto, Simona De Summa, Vito Michele Garrisi, Sabino Strippoli, Amalia Azzariti, Gabriella Guida, Michele Guida, Stefania Tommasi, Nicolas Jacquelot, David Enot, Caroline Flament, Jonathan M. Pitt, Nadège Vimond, Carolin Blattner, Takahiro Yamazaki, Maria-Paula Roberti, Marie Vetizou, Romain Daillere, Vichnou Poirier-Colame, Michaë la Semeraro, Anne Caignard, Craig L Slingluff, Federica Sallusto, Sylvie Rusakiewicz, Benjamin Weide, Aurélien Marabelle, Holbrook Kohrt, Stéphane Dalle, Andréa Cavalcanti, Guido Kroemer, Anna Maria Di Giacomo, Michaele Maio, Phillip Wong, Jianda Yuan, Viktor Umansky, Alexander Eggermont, Laurence Zitvogel, Passarelli Anna, Tucci Marco, Stucci Stefania, Mannavola Francesco, Capone Mariaelena, Madonna Gabriele, Ascierto Paolo Antonio, Silvestris Franco, María Paula Roberti, David P. Enot, Michaela Semeraro, Sarah Jégou, Camila Flores, Tseng-hui Timothy Chen, Byoung S. Kwon, Ana Carrizossa Anderson, Christophe Borg, François Aubin, Maha Ayyoub, Anna Lisa De Presbiteris, Fabiola Gilda Cordaro, Rosa Camerlingo, Federica Fratangelo, Nicola Mozzillo, Giuseppe Pirozzi, Eduardo J. Patriarca, Emilia Caputo, Maria Letizia Motti, Rosaria Falcon, Roberta Miceli, Mariaelena Capone, Gabriele Madonna, Domenico Mallardo, Maria Vincenza Carrier, Elisabetta Panza, Paola De Cicco, Chiara Armogida, Giuseppe Ercolano, Gerardo Botti, Giuseppe Cirino, Angela Sandru, Miri Blank, Timea Balatoni, Judit Olasz, Emil Farkas, Andras Szollar, Akos Savolt, Maria Godeny, Orsolya Csuka, Szabolcs Horvath, Klara Eles, Yehuda Shoenfeld, Miklos Kasler, Susan Costantini, Francesca Capone, Farnaz Moradi, Pontus Berglund, Karin Leandersson, Rickard Linnskog, Tommy Andersson, Chandra Prakash Prasad, Cristiana Lo Nigro, Laura Lattanzio, Hexiao Wang, Charlotte Proby, Nelofer Syed, Marcella Occelli, Carolina Cauchi, Marco Merlano, Catherine Harwood, Alastair Thompson, Tim Crook, Katia Bifulco, Vincenzo Ingangi, Michele Minopoli, Concetta Ragone, Antonello Pessi, Francesco Mannavola, Stella D’Oronzo, Claudia Felici, Marco Tucci, Antonio Doronzo, Franco Silvestris, Anna Ferretta, Stefania Guida, Imma Maida, Tiziana Cocco, Anna Passarelli, Davide Quaresmini, Ornella Franzese, Belinda Palermo, Cosmo Di Donna, Isabella Sperduti, MariaLaura Foddai, Helena Stabile, Angela Gismondi, Angela Santoni, Paola Nisticò, Andrea P. Sponghini, Francesca Platini, Elena Marra, David Rondonotti, Oscar Alabiso, Maria T. Fierro, Paola Savoia, Florian Stratica, Pietro Quaglino, Gianluca Di Monta, Caracò Corrado, Massimiliano Di Marzo, Marone Ugo, Maria Luisa Di Cecilia, Mozzillo Nicola, Celeste Fusciello, Antonio Marra, Rosario Guarrasi, Carlo Baldi, Rosa Russo, Giovanni Di Giulio, Vincenzo Faiola, Pio Zeppa, Stefano Pepe, Elisabetta Gambale, Consiglia Carella, Alessandra Di Paolo, Michele De Tursi, Laura Marra, Fara De Murtas, Valeria Sorrentino, Silviu Voinea, Eugenia Panaitescu, Madalina Bolovan, Adina Stanciu, Sabin Cinca, Chiara Botti, Gabriella Aquino, Annamaria Anniciello, Cristina Fortes, Simona Mastroeni, Alessio Caggiati, Francesca Passarelli, Alba Zappalà, Maria Capuano, Riccardo Bono, Maurizio Nudo, Claudia Marino, Paola Michelozzi, Valeria De Biasio, Vincenzo C. Battarra, Silvia Formenti, Maria Libera Ascierto, Tracee L. McMiller, Alan E. Berger, Ludmila Danilova, Robert A. Anders, George J. Netto, Haiying Xu, Theresa S. Pritchard, Jinshui Fan, Chris Cheadle, Leslie Cope, Charles G. Drake, Drew M. Pardoll, Janis M. Taube, Suzanne L. Topalian, Sacha Gnjatic, Sarah Nataraj, Naoko Imai, Adeeb Rahman, Achim A. Jungbluth, Linda Pan, Ralph Venhaus, Andrew Park, Frédéric F. Lehmann, Nikoletta Lendvai, Adam D. Cohen, Hearn J. Cho, Speiser Daniel, Vera Hirsh

**Affiliations:** 1 Department of Melanoma Medical Oncology, University of Texas MD Anderson Cancer Center, Houston, TX USA; 2 Istituto Nazionale per lo Studio e la Cura dei Tumori “Fondazione G. Pascale”, Naples, Italy; 3 Dipartimento di Medicina Sperimentale e Clinica, Università degli Studi di Catanzaro “Magna Graecia”, Catanzaro, Italy; 4 Takis s.r.l., Rome, Italy; 5 Department of Molecular Virology, Immunology and Medical Genetics, The Ohio State University Comprehensive Cancer Center, Columbus, OH USA; 6 Dipartimento di Chirurgia “P. Valdoni”, Sapienza Università di Roma, Rome, Italy; 7 Dipartimento di Medicina Clinica e Molecolare, Sapienza Università di Roma, Rome, Italy; 8 Department of Pathology, University of Chicago, Chicago, IL USA; 9 Department of Medicine, University of Chicago, Chicago, IL USA; 10 Department of Surgery, Massachusetts General Hospital, Harvard Medical School, Boston, MA USA; 11 Department of Pathology, NYU School of Medicine, New York, NY USA; 12 Department of Radiation Oncology, NYU School of Medicine, New York, NY USA; 13 Department of Radiation Oncology, Weill Cornell Medical College, New York, NY USA; 14 Department of Pathology, University of Texas Southwestern, Dallas, USA; 15 Department of Dermatology, University of Zurich Hospital, Zurich, Switzerland; 16 Vanderbilt-Ingram Cancer Center, Vanderbilt University, Nashville, TN USA; 17 Memorial Sloan Kettering Cancer Center, New York, USA; 18 Melanoma and Skin Cancer Program, University of Pittsburgh Cancer Institute, Pittsburgh, Pennsylvania USA; 19 Department of Medicine and Division of Hematology/Oncology, University of Pittsburgh, School of Medicine, Pittsburgh, PA 15213 USA; 20 Department of Immunology, University of Pittsburgh, School of Medicine, Pittsburgh, PA 15213 USA; 21 Department of Biostatistics, School of Medicine, University of Pittsburgh, Pittsburgh, PA 15213 USA; 22 Biologics Discovery California, Bristol-Myers Squibb, Redwood City, CA 94063 USA; 23 Cell Processing Section, Department of Transfusion Medicine, Clinical Center, National Institutes of Health, Bethesda, MD USA; 24 Fondazione IRCCS Istituto Nazionale dei Tumori, Milan, Italy; 25 Cancer Diagnosis Program, Division of Cancer Treatment and Diagnosis, NCI, NIH, Rockville, MD USA; 26 Institute of Pathology, University of Bern, Bern, Switzerland; 27 Laboratory of Immunology, Hopital Européen Georges Pompidou, Paris, France; 28 INSERM team 15, Cordeliers Research Center, Paris, France; 29 Oncology Area, Spanish Medicines Agency and Medical Devices (AEMPS), Madrid, Spain; 30 Research Institute Hospital Puerta de Hierro Majadahonda, Madrid, Spain; 31 Department of Pharmaceutical Sciences, Università del Piemonte Orientale, Novara, Italy; 32 Cergas (Centre for Research on Health and Social Care Management), Università Bocconi, Milano, Italy; 33 Department of Oncology-Pathology, Cancer Center Karolinska, Karolinska Institutet, Stockholm, Sweden; 34 Division of Translational Medicine, Sidra Medical and Research Center, Doha, Qatar; 35 Division of Molecular Oncology of Gastrointestinal Tumors, German Cancer Research Center, Heidelberg, Germany; 36 Pathology Unit, Istituto Nazionale per lo Studio e la Cura dei Tumori Fondazione “G. Pascale”, Napoli, Italy; 37 Faculty of Pharmacy and Medicine, Department of Medicine, University of Salerno, Baronissi, Salerno, Italy; 38 Molecular Immunology and Immuneregulation, Istituto Nazionale per lo Studio e la Cura dei Tumori “Fondazione G. Pascale”, Napoli, Italy; 39 Department of Pharmacy, University of Naples Federico II, Napoli, Italy; 40 Unit of Medical Oncology and Innovative Therapy, Istituto Nazionale per lo Studio e la Cura Tumori Fondazione “G. Pascale”, Napoli, Italy; 41 Melanoma and Immunotherapies Service, Memorial Sloan Kettering Cancer Center, New York, NY USA; 42 Dermatology Unit, Institut Gustave Roussy, Paris, France; 43 Department of Medical Oncology and Hematology, The Royal Marsden Hospital, London, UK; 44 Division of Immunology, The Netherlands Cancer Institute, Amsterdam, The Netherlands; 45 Department of Medicine, Jonsson Comprehensive Cancer Center at University of California Los Angeles, Los Angeles, CA USA; 46 Department of Medicine, Princess Margaret Hospital and University Health Network, Toronto, Canada; 47 Melanoma Center, The Angeles Clinic and Research Institute, Los Angeles, CA USA; 48 Unit of Melanoma, Cancer Immunotherapy and Innovative Therapy, Istituto Nazionale Tumori Fondazione Pascale, Naples, Italy; 49 Divisione chirurgia dermatoncologica, Europeo di Oncologia, Milan, Italy; 50 Institute of Cancer Sciences, University of Manchester, Manchester, UK; 51 Dermatologische Klinik, University of Zurich, Zurich, Switzerland; 52 Division of Hematology and Oncology, Vanderbilt University School of Medicine, Nashville, TN USA; 53 Department of Medicine, Massachusetts General Hospital, Boston, MA USA; 54 Department of Biostatistics, Genentech Inc., San Francisco, CA USA; 55 Product Development Oncology, Genentech Inc., San Francisco, CA USA; 56 Dermatology Product Development, Genentech Inc., San Francisco, CA USA; 57 Department of Dermatology, University of Kiel, Kiel, Germany; 58 Skin and Melanoma Service, Peter MacCallum Cancer Centre, East Melbourne, Australia; 59 Yale University School of Medicine and Smilow Cancer Center, Yale-New Haven Hospital, New Haven, CT USA; 60 Weill Cornell Medical College, New York, NY USA; 61 Georgetown-Lombardi Comprehensive Cancer Center, Washington, DC USA; 62 University of Pittsburgh Medical Center, Pittsburgh, PA USA; 63 Bristol-Myers Squibb, Princeton, NJ USA; 64 Bristol-Myers Squibb, Lawrenceville, NJ USA; 65 Unit of Melanoma, Cancer Immunotherapy and Innovative Therapy, Istituto Nazionale Tumori Fondazione G. Pascale, Naples, Italy; 66 Skin and Melanoma Service, Peter MacCallum Cancer Centre, Melbourne, VIC Australia; 67 Division of Cancer Services National Institute of Oncology, Budapest, Hungary; 68 Division of Medical Oncology and Immunotherapy, Azienda Ospedaliera Universitaria Senese, Siena, Italy; 69 Division of Medical Oncology, Papa Giovanni XXIII Hospital, Bergamo, Italy; 70 Department of Biotherapy, N. N. Blokhin Russian Cancer Research Center, Moscow, Russia; 71 Department of Chemotherapy, Moscow City Oncology Hospital 62, Krasnogorsk, Russia; 72 Department of Dermatology, Centre Hospitalier Lyon Sud, Pierre-Bénite, France; 73 Department of Clinical Oncology, Hospital Universitario Virgen Macarena, Seville, Spain; 74 Division of Cancer Services, Princess Alexandra Hospital, Woolloongabba, QLD Australia; 75 Department of Dermato-Oncology, Hôpital Saint André, Bordeaux, France; 76 Deptartment of Dermatology, University of Tübingen, Tübingen, Germany; 77 Oncology Biomarker Development, Genentech Inc., South San Francisco, CA USA; 78 Product Development Oncology, Genentech, Inc., South San Francisco, CA USA; 79 Department of Dermato Cancerology, Nantes University, Nantes, France; 80 Dana-Farber Cancer Institute, Boston, MD USA; 81 The Angeles Clinic and Research Institute, Los Angeles, CA USA; 82 Florida Cancer Specialists/Sarah Cannon Research Institute, Sarasota, FL USA; 83 The University of Texas MD Anderson Cancer Center, Houston, TX USA; 84 Genentech, Inc., South San Francisco, CA USA; 85 Department of Pathology and Department of Medicine-Section of Hematology/Oncology, University of Chicago, Chicago, IL USA; 86 Research Department, The Angeles Clinic and Research Institute, Los Angeles, CA USA; 87 Department of Internal Medicine and Department of Urology, University of Michigan, Ann Arbor, MI USA; 88 Drug Development Unit, Sarah Cannon Research Institute/Tennessee Oncology PLLC, Nashville, TN USA; 89 Division of Hematology and Medical Oncology, University of Connecticut Health Center, Farmington, CT USA; 90 Department of Medicine-Section of Hematology/Oncology, University of Chicago, Chicago, USA; 91 Merck Research Laboratories, Merck & Co. Inc., Kenilworth, NJ USA; 92 Incyte Corporation, Drug Development, Wilmington, DE USA; 93 Hematology-Oncology Division, Abramson Cancer Center of the University of Pennsylvania, Philadelphia, PA USA; 94 University Hospital of Zurich, Zurich, Switzerland; 95 Melanoma Institute Australia, The University of Sydney and The Mater Hospital, Sydney, Australia; 96 University of California at Los Angeles Medical Center, Los Angeles, CA USA; 97 Vanderbilt University Medical Center, Nashville, TN USA; 98 Centre Hospitalier Universitaire Vaudois, Lausanne, Switzerland; 99 The West Clinic, Memphis, TN USA; 100 University of Utah Huntsman Cancer Institute, Salt Lake City, UT USA; 101 Olivia Newton-John Cancer Center, Heidelberg, Australia; 102 Hopitaux Universitaires de Geneve;, Geneva, Switzerland; 103 Hospital Clinic i Provincial de Barcelona, Barcelona, Spain; 104 Fox Chase Cancer Center, Philadelphia, PA USA; 105 The University of Chicago Medicine, Chicago, IL USA; 106 University of Pittsburgh Cancer Institute, Pittsburgh, PA USA; 107 Merck & Co., Inc., Kenilworth, NJ USA; 108 Amgen Inc., Thousand Oaks, CA USA; 109 Princess Alexandra Hospital, Gallipoli Medical Research Foundation, Woolloongabba, Greenslopes, QLD Australia; 110 Istituto Nazionale Tumori Fondazione Pascale, Naples, Italy; 111 Melanoma Institute Australia, Sydney, NSW Australia; 112 Cabrini Health, Melbourne, VIC Australia; 113 Hôpital Saint André Centre Hospitalier Universitaire, Bordeaux, France; 114 University Hospital of Siena, Siena, Italy; 115 Hôpital Claude Huriez, Lille, France; 116 University Hospital Heidelberg, Heidelberg, Germany; 117 Sklodowska-Curie Memorial Cancer Center, Warsaw, Poland; 118 Chris O’Brien Lifehouse, Royal Prince Alfred Hospital and Melanoma Institute Australia, Camperdown, NSW Australia; 119 Wojewódzki Szpital Specjalistyczny im. M., Lodz, Poland; 120 Hôpital Saint-Louis University Paris Diderot, Paris, France; 121 University of Gothenburg, Gothenburg, Sweden; 122 Instituto Alexander Fleming, Buenos Aires, Argentina; 123 IRCCS San Martino, IST Istituto Nazionale per la Ricerca sul Cancro, Genova, Italy; 124 University Hospital Mainz, 55122 Mainz, Germany; 125 Sunnybrook Health Sciences Centre, Toronto, Canada; 126 Consorcio Hospital General Universitario de Valencia, Valencia, Spain; 127 Onkologikoa, Donosti, Gipuzkoa Spain; 128 Rambam Health Care Campus, Haifa, Israel; 129 Bristol-Myers Squibb, Hopewell, NJ USA; 130 Gustave Roussy, Paris-Sud University, Villejuif Paris-Sud, France; 131 Melanoma Institute Australia, The University of Sydney, and Mater Hospital, Sydney, NSW Australia; 132 Moffitt Cancer Center, Tampa, FL USA; 133 Gallipoli Medical Research Foundation and Princess Alexandra Hospital, Greenslopes, QLD Australia; 134 Hospital Timone APHM, Aix-Marseille University, Marseille, France; 135 Universitaets Spital, Zurich, Switzerland; 136 Gustave Roussy and Paris-Sud University, Villejuif Paris-Sud, France; 137 Servicio de Oncología Médica, Hospital General Universitario Gregorio Marañón, Madrid, Spain; 138 Chris O’Brien Lifehouse, The Melanoma Institute Australia and Royal Prince Alfred Hospital, Camperdown, Australia; 139 Department of Oncology, Aarhus University Hospital, Aarhus, Denmark; 140 Department of Medical Oncology, Coffs Harbour Health Campus, Coffs Harbour, NSW Australia; 141 Cliniques Universitaires Saint-Luc, Bruxelles, Belgium; 142 Ludwig Center at Memorial Sloan Kettering Cancer Center, New York, NY USA; 143 Istituto Tumori “Giovanni Paolo II, Bari, Italy; 144 Università di Bari, Bari, Italy; 145 INSERM U1015, Gustave Roussy Cancer Institute, Villejuif, France; 146 University Paris Sud XI, Kremlin Bicêtre, France; 147 Gustave Roussy Cancer Institute, Villejuif, France; 148 Metabolomics and Cell Biology Platforms, Gustave Roussy Cancer Institute, Villejuif, France; 149 CIC Biotherapie IGR Curie, CIC1428, Gustave Roussy Cancer Institute, Villejuif, France; 150 Laboratory of Immunomonitoring in Oncology (L.I.O), UMS 3655 CNRS / US 23 INSERM, Gustave Roussy Cancer Institute, Villejuif, France; 151 Skin Cancer Unit, German Cancer Research Center (DKFZ), Heidelberg, Germany; 152 Department of Dermatology, Venereology and Allergology, University Medical Center Mannheim Ruprecht-Karl University of Heidelberg, Mannheim, Germany; 153 Center of Clinical Investigation Hôpital Necker Enfants Malades, Paris, France; 154 INSERM U1016, CNRS UMR8104 Cochin Institute, Paris, France; 155 Division of Surgical Oncology, Department of Surgery, University of Virginia, Charlottesville, VA USA; 156 Cellular Immunology Laboratory, Institute for Research in Biomedicine, Università della Svizzera Italiana (USI), Via Vincenzo Vela 6, 6500 Bellinzona, Switzerland; 157 Department of Immunology, Eberhard Karls Universität Tübingen, Tübingen, Germany; 158 Department of Dermatology, University Medical Center Tübingen, Tübingen, Germany; 159 Division of Oncology, Department of Medicine, Stanford University, Stanford, CA USA; 160 Centre Hospitalier Lyon-Sud, Hospices Civils de Lyon, University Claude Bernard Lyon 1, Lyon, France; 161 Equipe 11 labellisée par la Ligue contre le Cancer, Centre de Recherche des Cordeliers, Paris, France; 162 INSERM, U1138, Paris, France; 163 Université Paris Descartes, Sorbonne Paris Cité, Paris, France; 164 Université Pierre et Marie Curie, Paris, France; 165 Pôle de Biologie, Hôpital Européen Georges Pompidou, AP-HP, Paris, France; 166 Medical Oncology and Immunotherapy Division, University Hospital of Siena, Viale Bracci, 14, 53100 Siena, Italy; 167 Medical Oncology and Immunotherapy, Department of Oncology,, University Hospital of Siena, Istituto Toscano Tumori, Siena, Italy; 168 Ludwig Center for Cancer Immunotherapy, Department of Immunology and Department of Medicine,, Memorial Sloan-Kettering Cancer Center, New York, NY USA; 169 Medical Oncology Unit, Department of Biomedical Sciences and Clinical Oncology, University of Bari ‘Aldo Moro’, Bari, Italy; 170 Melanoma, Cancer Immunotherapy and Innovative Therapy Unit, INT “G. Pascale”, Napoli, Italy; 171 INSERM U1015, Gustave Roussy Cancer Institute, Villejuif, France; 172 Gustave Roussy Cancer Campus, Villejuif, France; 173 CIC Biothérapie IGR Curie CIC1428, Gustave Roussy Cancer Institute, Villejuif, France; 174 Metabolomics and Cell Biology Platforms, Gustave Roussy Cancer Institute, Villejuif, France; 175 Center of Clinical Investigation Hôpital Necker Enfants Malades, Paris, France; 176 INSERM U1138, Centre de Recherche des Cordeliers, Paris, France; 177 Department of Immunobiology, Yale School of Medicine, 10 Amistad Street, New Haven, CT 06519 USA; 178 Cancer Immunology Branch, Division of Cancer Biology, National Cancer Center, Ilsan, Goyang, Gyeonggi Korea; 179 Section of Clinical Immunology, Allergy, and Rheumatology, Department of Medicine, Tulane University Health Sciences Center, New Orleans, LA USA; 180 Evergrande Center for Immunologic Diseases and Ann Romney Center for Neurologic Diseases, Brigham and Women’s Hospital and Harvard Medical School, Boston, MA 02115 USA; 181 Departments of Dermatology, Cancer Institute Gustave Roussy, Villejuif, France; 182 Clinical Oncology, Melanoma Branch, Gustave Roussy Cancer Institute, Villejuif, France; 183 Department of Medical Oncology, University Hospital of Besancon, 3 Boulevard Alexander Fleming, Besancon, 25030 France; 184 Clinical Investigational Centre, CIC-1431, University Hospital of Besançon, Besançon, France; 185 INSERM U1098, University of Franche-Comté, Besançon, France; 186 Centre Hospitalier Lyon-Sud, Hospices Civils de Lyon, University Claude Bernard Lyon 1, Lyon, France; 187 Equipe 11 labellisée par la Ligue contre le Cancer, Centre de Recherche des Cordeliers, Paris, France; 188 Université Paris Descartes, Sorbonne Paris Cité, Paris, France; 189 Université Pierre et Marie Curie, Paris, France; 190 Pôle de Biologie, Hôpital Européen Georges Pompidou, AP-HP, Paris, France; 191 Division of Oncology, Department of Medicine, Stanford University, Stanford, CA USA; 192 Department of Surgery, Gustave Roussy Cancer Campus, Villejuif, France; 193 Saint Antoine Hospital, INSERM ERL 1157-CNRS UMR 7203, Paris, France; 194 Medical Oncology and Immunotherapy, Department of Oncology, University Hospital of Siena, Istituto Toscano Tumori, Siena, Italy; 195 Memorial Sloan-Kettering Cancer Center, Ludwig Institute for Cancer Research, Weill Cornell Medical College, New York, NY USA; 196 Division of Dermatooncology, Department of Dermatology, University Medical Center, Tübingen, Germany; 197 EA3181, SFR4234, Service de Dermatologie, Université de Franche Comté, Centre Hospitalier Universitaire (CHU), Besançon, France; 198 Institute of Genetics and Biophysics –I.G.B., A. Buzzati-Traverso– CNR, Naples, Italy; 199 Istituto Nazionale Tumori Fondazione G. Pascale, Naples, Italy; 200 IRCCS Istituto Nazionale Tumori “Fondazione G. Pascale”, Naples, Italy; 201 Institute of Genetics and Biophysics –I.G.B.– CNR, Naples, Italy; 202 University Parthenope, Naples, Italy; 203 Department of Experimental Oncology, Istituto Nazionale Tumori Fondazione “G. Pascale”, Naples, Italy; 204 Unit of Pathology, Istituto Nazionale per lo Studio e la cura dei tumori “Fondazione G. Pascale” IRCCS, Naples, Italy; 205 Department of Physiology and Pharmacology, University of Calgary, Calgary, Alberta Canada; 206 Molecular Immunology and Toxicology, National Institute of Oncology, Budapest, Hungary; 207 Center of Surgical and Molecular Tumorpathology, National Institute of Oncology, Budapest, Hungary; 208 Oncodermatology, National Institute of Oncology, Budapest, Hungary; 209 Zabludowitz Center for Autoimmune Diseases, Sheba Medical Center affiliated to Sackler Faculty of Medicine, Tel-Aviv University, Tel Aviv, Israel; 210 Pathogenetics, National Institute of Oncology, Budapest, Hungary; 211 Oncosurgery, National Institute of Oncology, Budapest, Hungary; 212 Department of Radiological Diagnostics, National Institute of Oncology, Budapest, Hungary; 213 Board of Directors, National Institute of Oncology, Budapest, Hungary; 214 Department of Cell and Experimental Pathology, Lund University, Lund, Sweden; 215 Center for Molecular Pathology, Institution of Translational Medicine, Lund University, Lund, Sweden; 216 Laboratory of Cancer Genetics and Translational Oncology, S. Croce & amp, Carle Teaching Hospital, Cuneo, Italy; 217 Dundee Cancer Center, University of Dundee, Ninewells Hospital and Medical School, Dundee, Scotland, UK; 218 Faculty of Medicine, Imperial College London, London, UK; 219 Medical Oncology, Oncology Department, S. Croce & amp, Carle Teaching Hospital, Cuneo, Italy; 220 Barts and the London School of Medicine and Dentistry, Queen Mary University of London, London, UK; 221 IRCCS Istituto Nazionale Tumori de “Fondazione G. Pascale”, Naples, Italy; 222 University ‘Parthenope’, Naples, Italy; 223 PeptiPharma, Viale Città D’Europa 679, 00144 Rome, Italy; 224 Medical Oncology Unit, University of Bari ‘Aldo Moro’, Bari, Italy; 225 Istituto Tumori “Giovanni Paolo II” I.R.R.C.S., Istituto di ricovero e cura a carattere scientifico, Bari, Italy; 226 Dipartimento di Scienze Mediche di Base, Neuroscienze e Organi di Senso, Universita degli Studi di Bari, Bari, Italy; 227 Department of Systems Medicine, University of Tor Vergata, Rome, Italy; 228 Department of Research, Advanced Diagnostics and Technological Innovation, Regina Elena National Cancer Institute, Rome, Italy; 229 Biostatistics and Scientific Direction, Regina Elena National Cancer Institute, Rome, Italy; 230 Department of Molecular Medicine, University of Rome “La Sapienza”, Rome, Italy; 231 A.O.U. Maggiore della Carità, S.C. di Oncologia, Novara, Italy; 232 A.O.U. Città della Salute e della Scienza, S.C. di Dermatologia, Torino, Italy; 233 Department Melanoma, Division Surgery of Melanoma, National Cancer Institute “Fondazione Pascale”, Naples, Italy; 234 Department of Onco-Hematology, AOU S. Giovanni di Dio e Ruggi D’Aragona, University of Salerno, Salerno, Italy; 235 Department of Medical, Oral and Biotechnological Sciences, University G. D’Annunzio of Chieti-Pescara, Chieti, Italy; 236 Pathology Unit, Istituto Nazionale Tumori Fondazione “G. Pascale”, Napoli, Italy; 237 Department of Surgical Oncology, Alexandru Trestioreanu” Oncologic Institute, “Carol Davila” University of Medicine and Pharmacy, Bucharest, Romania; 238 Department of Medical Informatics and Biostatistics, “Carol Davila” University of Medicine and Pharmacy, Bucharest, Romania; 239 Department of Carcinogenesis and Molecular Biology, “Alexandru Trestioreanu” Oncologic Institute, Bucharest, Romania; 240 Department of Surgical Oncology, “Carol Davila” University of Medicine and Pharmacy, “Alexandru Trestioreanu” Oncologic Institute, Bucharest, Romania; 241 Unit of Melanoma, Cancer Immunotherapy and Innovative Therapy, Istituto Nazionale Tumori Fondazione “G. Pascale”, Napoli, Italy; 242 Pathology Unit, Istituto Nazionale Tumori de Fondazione “G. Pascale”, Napoli, Italy; 243 Epidemiology Unit, IDI-IRCCS, Rome, Italy; 244 Plastic Surgery Unit, IDI-IRCCS, Rome, Italy; 245 Pathology Unit, IDI-IRCCS, Rome, Italy; 246 Oncology Unit, IDI-IRCCS, Rome, Italy; 247 Division Dermatology Villa Paola, Capranica, Italy; 248 Division Dermatology, IDI-IRCCS, Rome, Italy; 249 Department of Epidemiology of the Regional Health Service, Rome, Italy; 250 Mental Health Department, Hospital of Lauria, Rome, Italy; 251 Dermatology and Oncology surgery of skin growths Department, Hospital of Caserta, Caserta, Italy; 252 Department of Oncology, The Johns Hopkins University School of Medicine and Sidney Kimmel Comprehensive Cancer Center, Baltimore, MD 21287 USA; 253 Department of Surgery, The Johns Hopkins University School of Medicine and Sidney Kimmel Comprehensive Cancer Center, Baltimore, MD 21287 USA; 254 The Lowe Family Genomics Core, The Johns Hopkins University School of Medicine and Sidney Kimmel Comprehensive Cancer Center, Baltimore, MD 21287 USA; 255 Oncology Bioinformatics Core, The Johns Hopkins University School of Medicine and Sidney Kimmel Comprehensive Cancer Center, Baltimore, MD 21287 USA; 256 Department of Pathology, The Johns Hopkins University School of Medicine and Sidney Kimmel Comprehensive Cancer Center, Baltimore, MD 21287 USA; 257 The James Buchanan Brady Urological Institute, The Johns Hopkins University School of Medicine and Sidney Kimmel Comprehensive Cancer Center, Baltimore, MD 21287 USA; 258 Department of Dermatology, The Johns Hopkins University School of Medicine and Sidney Kimmel Comprehensive Cancer Center, Baltimore, MD 21287 USA; 259 Icahn School of Medicine at Mount Sinai, New York city, USA; 260 Ludwig Institute for Cancer Research, New York, NY USA; 261 GSK Vaccines, Rixensart, Belgium; 262 University of Pennsylvania, Philadelphia, PA USA; 263 Department of Oncology and Ludwig Cancer Research Center, University of Lausanne, Lausanne, Switzerland; 264 Campbell Family Institute, Princess Margaret Hospital, Toronto, Canada; 265 McGill University, MUHC Royal Victoria Hospital, Montreal, QC Canada

## Abstract

MELANOMA BRIDGE 2015

KEYNOTE SPEAKER PRESENTATIONS

Molecular and immuno-advances

K1 Immunologic and metabolic consequences of PI3K/AKT/mTOR activation in melanoma

Vashisht G. Y. Nanda, Weiyi Peng, Patrick Hwu, Michael A. Davies

K2 Non-mutational adaptive changes in melanoma cells exposed to BRAF and MEK inhibitors help the establishment of drug resistance

Gennaro Ciliberto, Luigi Fattore, Debora Malpicci, Luigi Aurisicchio, Paolo Antonio Ascierto, Carlo M. Croce, Rita Mancini

K3 Tumor-intrinsic beta-catenin signaling mediates tumor-immune avoidance

Stefani Spranger, Thomas F. Gajewski

K4 Intracellular tumor antigens as a source of targets of antibody-based immunotherapy of melanoma

Yangyang Wang, Soldano Ferrone

Combination therapies

K5 Harnessing radiotherapy to improve responses to immunotherapy in cancer

Claire Vanpouille-Box, Erik Wennerberg, Karsten A. Pilones, Silvia C. Formenti, Sandra Demaria

K6 Creating a T cell-inflamed tumor microenvironment overcomes resistance to checkpoint blockade

Haidong Tang, Yang Wang, Yang-Xin Fu

K7 Biomarkers for treatment decisions?

Reinhard Dummer

K8 Combining oncolytic therapies in the era of checkpoint inhibitors

Igor Puzanov

K9 Immune checkpoint blockade for melanoma: should we combine or sequence ipilimumab and PD-1 antibody therapy?

Michael A. Postow

News in immunotherapy

K10 An update on adjuvant and neoadjuvant therapy for melanom

Ahmad Tarhini

K11 Targeting multiple inhibitory receptors in melanoma

Joe-Marc Chauvin, Ornella Pagliano, Julien Fourcade, Zhaojun Sun, Hong Wang, Cindy Sanders, John M. Kirkwood, Tseng-hui Timothy Chen, Mark Maurer, Alan J. Korman, Hassane M. Zarour

K12 Improving adoptive immune therapy using genetically engineered T cells

David F. Stroncek

Tumor microenvironment and biomarkers

K13 Myeloid cells and tumor exosomes: a crosstalk for assessing immunosuppression?

Veronica Huber, Licia Rivoltini

K14 Update on the SITC biomarker taskforce: progress and challenges

Magdalena Thurin

World-wide immunoscore task force: an update

K15 The immunoscore in colorectal cancer highlights the importance of digital scoring systems in surgical pathology

Tilman Rau, Alessandro Lugli

K16 The immunoscore: toward an integrated immunomonitoring from the diagnosis to the follow up of cancer’s patients

Franck Pagès

Economic sustainability of melanoma treatments: regulatory, health technology assessment and market access issues

K17 Nivolumab, the regulatory experience in immunotherapy

Jorge Camarero, Arantxa Sancho

K18 Evidence to optimize access for immunotherapies

Claudio Jommi

ORAL PRESENTATIONS

Molecular and immuno-advances

O1 Ipilimumab treatment results in CD4 T cell activation that is concomitant with a reduction in Tregs and MDSCs

Yago Pico de Coaña, Maria Wolodarski, Yuya Yoshimoto, Giusy Gentilcore, Isabel Poschke, Giuseppe V. Masucci, Johan Hansson, Rolf Kiessling

O2 Evaluation of prognostic and therapeutic potential of COX-2 and PD-L1 in primary and metastatic melanoma

Giosuè Scognamiglio, Francesco Sabbatino, Federica Zito Marino, Anna Maria Anniciello, Monica Cantile, Margherita Cerrone, Stefania Scala, Crescenzo D’alterio, Angela Ianaro, Giuseppe Cirino, Paolo Antonio Ascierto, Giuseppina Liguori, Gerardo Botti

O3 Vemurafenib in patients with BRAFV600 mutation–positive metastatic melanoma: final overall survival results of the BRIM-3 study

Paul B. Chapman, Caroline Robert, James Larkin, John B. Haanen, Antoni Ribas, David Hogg, Omid Hamid, Paolo Antonio Ascierto, Alessandro Testori, Paul Lorigan, Reinhard Dummer, Jeffrey A. Sosman, Keith T. Flaherty, Huibin Yue, Shelley Coleman, Ivor Caro, Axel Hauschild, Grant A. McArthur

O4 Updated survival, response and safety data in a phase 1 dose-finding study (CA209-004) of concurrent nivolumab (NIVO) and ipilimumab (IPI) in advanced melanoma

Mario Sznol, Margaret K. Callahan, Harriet Kluger, Michael A. Postow, RuthAnn Gordan, Neil H. Segal, Naiyer A. Rizvi, Alexander Lesokhin, Michael B. Atkins, John M. Kirkwood, Matthew M. Burke, Amanda Ralabate, Angel Rivera, Stephanie A. Kronenberg, Blessing Agunwamba, Mary Ruisi, Christine Horak, Joel Jiang, Jedd Wolchok

Combination therapies

O5 Efficacy and correlative biomarker analysis of the coBRIM study comparing cobimetinib (COBI) + vemurafenib (VEM) vs placebo (PBO) + VEM in advanced BRAF-mutated melanoma patients (pts)

Paolo A. Ascierto, Grant A. McArthur, James Larkin, Gabriella Liszkay, Michele Maio, Mario Mandalà, Lev Demidov, Daniil Stoyakovskiy, Luc Thomas, Luis de la Cruz-Merino, Victoria Atkinson, Caroline Dutriaux, Claus Garbe, Matthew Wongchenko, Ilsung Chang, Daniel O. Koralek, Isabelle Rooney, Yibing Yan, Antoni Ribas, Brigitte Dréno

O6 Preliminary clinical safety, tolerability and activity results from a Phase Ib study of atezolizumab (anti-PDL1) combined with vemurafenib in BRAFV600-mutant metastatic melanoma

Ryan Sullivan, Omid Hamid, Manish Patel, Stephen Hodi, Rodabe Amaria, Peter Boasberg, Jeffrey Wallin, Xian He, Edward Cha, Nicole Richie, Marcus Ballinger, Patrick Hwu

O7 Preliminary safety and efficacy data from a phase 1/2 study of epacadostat (INCB024360) in combination with pembrolizumab in patients with advanced/metastatic melanoma

Thomas F. Gajewski, Omid Hamid, David C. Smith, Todd M. Bauer, Jeffrey S. Wasser, Jason J. Luke, Ani S. Balmanoukian, David R. Kaufman, Yufan Zhao, Janet Maleski, Lance Leopold, Tara C. Gangadhar

O8 Primary analysis of MASTERKEY-265 phase 1b study of talimogene laherparepvec (T-VEC) and pembrolizumab (pembro) for unresectable stage IIIB-IV melanoma

Reinhard Dummer, Georgina V. Long, Antoni Ribas, Igor Puzanov, Olivier Michielin, Ari VanderWalde, Robert H.I. Andtbacka, Jonathan Cebon, Eugenio Fernandez, Josep Malvehy, Anthony J. Olszanski, Thomas F. Gajewski, John M. Kirkwood, Christine Gause, Lisa Chen, David R. Kaufman, Jeffrey Chou, F. Stephen Hodi

News in immunotherapy

O9 Two-year survival and safety update in patients (pts) with treatment-naïve advanced melanoma (MEL) receiving nivolumab (NIVO) or dacarbazine (DTIC) in CheckMate 066

Victoria Atkinson, Paolo A. Ascierto, Georgina V. Long, Benjamin Brady, Caroline Dutriaux, Michele Maio, Laurent Mortier, Jessica C. Hassel, Piotr Rutkowski, Catriona McNeil, Ewa Kalinka-Warzocha, Celeste Lebbé, Lars Ny, Matias Chacon, Paola Queirolo, Carmen Loquai, Parneet Cheema, Alfonso Berrocal, Karmele Mujika Eizmendi, Luis De La Cruz-Merino, Gil Bar-Sela, Christine Horak, Joel Jiang, Helene Hardy, Caroline Robert

O10 Efficacy and safety of nivolumab (NIVO) in patients (pts) with advanced melanoma (MEL) who were treated beyond progression in CheckMate 066/067

Georgina V. Long, Jeffrey S. Weber, James Larkin, Victoria Atkinson, Jean-Jacques Grob, Reinhard Dummer, Caroline Robert, Ivan Marquez-Rodas, Catriona McNeil, Henrik Schmidt, Karen Briscoe, Jean-François Baurain, F. Stephen Hodi, Jedd D. Wolchok

Tumor microenvironment and biomarkers

O11 New biomarkers for response/resistance to BRAF inhibitor therapy in metastatic melanoma

Rosamaria Pinto, Simona De Summa, Vito Michele Garrisi, Sabino Strippoli, Amalia Azzariti, Gabriella Guida, Michele Guida, Stefania Tommasi

O12 Chemokine receptor patterns in lymphocytes mirror metastatic spreading in melanoma and response to ipilimumab

Nicolas Jacquelot, David Enot, Caroline Flament, Jonathan M. Pitt, Nadège Vimond, Carolin Blattner, Takahiro Yamazaki, Maria-Paula Roberti, Marie Vetizou, Romain Daillere, Vichnou Poirier-Colame, Michaëla Semeraro, Anne Caignard, Craig L Slingluff Jr, Federica Sallusto, Sylvie Rusakiewicz, Benjamin Weide, Aurélien Marabelle, Holbrook Kohrt, Stéphane Dalle, Andréa Cavalcanti, Guido Kroemer, Anna Maria Di Giacomo, Michaele Maio, Phillip Wong, Jianda Yuan, Jedd Wolchok, Viktor Umansky, Alexander Eggermont, Laurence Zitvogel

O13 Serum levels of PD1- and CD28-positive exosomes before Ipilimumab correlate with therapeutic response in metastatic melanoma patients

Passarelli Anna, Tucci Marco, Stucci Stefania, Mannavola Francesco, Capone Mariaelena, Madonna Gabriele, Ascierto Paolo Antonio, Silvestris Franco

O14 Immunological prognostic factors in stage III melanomas

María Paula Roberti, Nicolas Jacquelot, David P Enot, Sylvie Rusakiewicz, Michaela Semeraro, Sarah Jégou, Camila Flores, Lieping Chen, Byoung S. Kwon, Ana Carrizossa Anderson, Caroline Robert, Christophe Borg, Benjamin Weide, François Aubin, Stéphane Dalle, Michele Maio, Jedd D. Wolchok, Holbrook Kohrt, Maha Ayyoub, Guido Kroemer, Aurélien Marabelle, Andréa Cavalcanti, Alexander Eggermont, Laurence Zitvogel

POSTER PRESENTATIONS

Molecular and immuno-advances

P1 Human melanoma cells resistant to B-RAF and MEK inhibition exhibit mesenchymal-like features

Anna Lisa De Presbiteris, Fabiola Gilda Cordaro, Rosa Camerlingo, Federica Fratangelo, Nicola Mozzillo, Giuseppe Pirozzi, Eduardo J. Patriarca, Paolo A. Ascierto, Emilia Caputo

P2 Anti-proliferative and pro-apoptotic effect of ABT888 on melanoma cell lines and its potential role in the treatment of melanoma resistant to B-RAF inhibitors

Federica Fratangelo, Rosa Camerlingo, Emilia Caputo, Maria Letizia Motti, Rosaria Falcone, Roberta Miceli, Mariaelena Capone, Gabriele Madonna, Domenico Mallardo, Maria Vincenza Carriero, Giuseppe Pirozzi and Paolo Antonio Ascierto

P3 Involvement of the L-cysteine/CSE/H2S pathway in human melanoma progression

Elisabetta Panza, Paola De Cicco, Chiara Armogida, Giuseppe Ercolano, Rosa Camerlingo, Giuseppe Pirozzi, Giosuè Scognamiglio, Gerardo Botti, Giuseppe Cirino, Angela Ianaro

P4 Cancer stem cell antigen revealing pattern of antibody variable region genes were defined by immunoglobulin repertoire analysis in patients with malignant melanoma

Beatrix Kotlan, Gabriella Liszkay, Miri Blank, Timea Balatoni, Judit Olasz, Emil Farkas, Andras Szollar, Akos Savolt, Maria Godeny, Orsolya Csuka, Szabolcs Horvath, Klara Eles, Yehuda Shoenfeld and Miklos Kasler

P5 Upregulation of Neuregulin-1 expression is a hallmark of adaptive response to BRAF/MEK inhibitors in melanoma

Debora Malpicci, Luigi Fattore, Susan Costantini, Francesca Capone, Paolo Antonio Ascierto, Rita Mancini, Gennaro Ciliberto

P6 HuR positively regulates migration of HTB63 melanoma cells

Farnaz Moradi, Pontus Berglund, Karin Leandersson, Rickard Linnskog, Tommy Andersson, Chandra Prakash Prasad

P7 Prolyl 4- (C-P4H) hydroxylases have opposing effects in malignant melanoma: implication in prognosis and therapy

Cristiana Lo Nigro, Laura Lattanzio, Hexiao Wang, Charlotte Proby, Nelofer Syed, Marcella Occelli, Carolina Cauchi, Marco Merlano, Catherine Harwood, Alastair Thompson, Tim Crook

P8 Urokinase receptor antagonists: novel agents for the treatment of melanoma

Maria Letizia Motti, Katia Bifulco, Vincenzo Ingangi, Michele Minopoli, Concetta Ragone, Federica Fratangelo, Antonello Pessi, Gennaro Ciliberto, Paolo Antonio Ascierto, Maria Vincenza Carriero

P9 Exosomes released by melanoma cell lines enhance chemotaxis of primary tumor cells

Francesco Mannavola, Stella D’Oronzo, Claudia Felici, Marco Tucci, Antonio Doronzo, Franco Silvestris

P10 New insights in mitochondrial metabolic reprogramming in melanoma

Anna Ferretta, Gabriella Guida, Stefania Guida, Imma Maida, Tiziana Cocco, Sabino Strippoli, Stefania Tommasi, Amalia Azzariti, Michele Guida

P11 Lenalidomide restrains the proliferation in melanoma cells through a negative regulation of their cell cycle

Stella D’Oronzo, Anna Passarelli, Claudia Felici, Marco Tucci, Davide Quaresmini, Franco Silvestris

Combination therapies

P12 Chemoimmunotherapy elicits polyfunctional anti-tumor CD8 + T cells depending on the activation of an AKT pathway sustained by ICOS

Ornella Franzese, Belinda Palermo, Cosmo Di Donna, Isabella Sperduti, MariaLaura Foddai, Helena Stabile, Angela Gismondi, Angela Santoni, Paola Nisticò

P13 Favourable toxicity profile of combined BRAF and MEK inhibitors in metastatic melanoma patients

Andrea P. Sponghini, Francesca Platini, Elena Marra, David Rondonotti, Oscar Alabiso, Maria T. Fierro, Paola Savoia, Florian Stratica, Pietro Quaglino

P14 Electrothermal bipolar vessel sealing system dissection reduces seroma output or time to drain removal following axillary and ilio-inguinal node dissection in melanoma patients: a pilot study

Di Monta Gianluca, Caracò Corrado, Di Marzo Massimiliano, Marone Ugo, Di Cecilia Maria Luisa, Mozzillo Nicola

News in immunotherapy

P15 Clinical and immunological response to ipilimumab in a metastatic melanoma patient with HIV infection

Francesco Sabbatino, Celeste Fusciello1, Antonio Marra, Rosario Guarrasi, Carlo Baldi, Rosa Russo, Di Giulio Giovanni, Vincenzo Faiola, Pio Zeppa, Stefano Pepe

P16 Immunotherapy and hypophysitis: a case report

Elisabetta Gambale, Consiglia Carella, Alessandra Di Paolo, Michele De Tursi

Tumor microenvironment and biomarkers

P17 New immuno- histochemical markers for the differential diagnosis of atypical melanocytic lesions with uncertain malignant potential

Laura Marra, Giosuè Scognamiglio, Monica Cantile, Margherita Cerrone, Fara De Murtas, Valeria Sorrentino, Anna Maria Anniciello, Gerardo Botti

P18 Utility of simultaneous measurement of three serum tumor markers in melanoma patients

Angela Sandru, Silviu Voinea, Eugenia Panaitescu, Madalina Bolovan, Adina Stanciu, Sabin Cinca

P19 The significance of various cut-off levels of melanoma inhibitory activity in evaluation of cutaneous melanoma patients

Angela Sandru, Silviu Voinea, Eugenia Panaitescu, Madalina Bolovan, Adina Stanciu, Sabin Cinca

P20 The long noncoding RNA HOTAIR is associated to metastatic progression of melanoma and it can be identified in the blood of patients with advanced disease

Chiara Botti, Giosuè Scognamiglio, Laura Marra, Gabriella Aquino, Rosaria Falcone, Annamaria Anniciello, Paolo Antonio Ascierto, Gerardo Botti, Monica Cantile

Other

P21 The effect of Sentinel Lymph Node Biopsy in melanoma mortality: timing of dissection

Cristina Fortes, Simona Mastroeni, Alessio Caggiati, Francesca Passarelli, Alba Zappalà, Maria Capuano, Riccardo Bono, Maurizio Nudo, Claudia Marino, Paola Michelozzi

P22 Epidemiological survey on related psychopathology in melanoma

Valeria De Biasio, Vincenzo C. Battarra

IMMUNOTHERAPY BRIDGE

KEYNOTE SPEAKER PRESENTATIONS

Immunotherapy beyond melanoma

K19 Predictor of response to radiation and immunotherapy

Silvia Formenti

K20 Response and resistance to PD-1 pathway blockade: clues from the tumor microenvironment

Maria Libera Ascierto, Tracee L. McMiller, Alan E. Berger, Ludmila Danilova, Robert A. Anders, George J. Netto, Haiying Xu, Theresa S. Pritchard, Jinshui Fan, Chris Cheadle, Leslie Cope, Charles G. Drake, Drew M. Pardoll, Janis M. Taube and Suzanne L. Topalian

K21 Combination immunotherapy with autologous stem cell transplantation, protein immunization, and PBMC reinfusion in myeloma patients

Sacha Gnjatic, Sarah Nataraj, Naoko Imai, Adeeb Rahman, Achim A. Jungbluth, Linda Pan, Ralph Venhaus, Andrew Park, Frédéric F. Lehmann, Nikoletta Lendvai, Adam D. Cohen, and Hearn J. Cho

K22 Anti-cancer immunity despite T cell “exhaustion”

Speiser Daniel

Immunotherapy in oncology (I-O): data from clinical trial

K23 The Checkpoint Inhibitors for the Treatment of Metastatic Non-small Cell Lung Cancer (NSCLC)

Vera Hirsh

## MELANOMA BRIDGE 2015

### KEYNOTE SPEAKER PRESENTATIONS

#### Molecular and immuno-advances

##### K1 Immunologic and metabolic consequences of PI3K/AKT/mTOR activation in melanoma

###### Vashisht G. Y. Nanda, Weiyi Peng, Patrick Hwu, Michael A. Davies

####### Department of Melanoma Medical Oncology, University of Texas MD Anderson Cancer Center, Houston, TX, USA

######## **Correspondence:** Michael A. Davies

*Journal of Translational Medicine* 2016, **14(Suppl 1)**:K1

**Background:** The PI3K/AKT/mTOR signaling pathway has been implicated in multiple cancers, and as a regulator of many key oncogenic processes. Our studies implicate a role for this pathway in resistance to both targeted and immune therapies for melanoma.

**Materials and methods:** Melanoma cell lines and clinical specimens were utilized to study the significance and functional consequences of the PI3K/AKT/mTOR pathway. Analyses of clinical specimens were performed under institution review board-approved protocols.

**Results:** Pilot whole genome expression profiling and synthetic lethality screens implicated oxidative phosphorylation (OxPhos) in resistance to BRAF and MEK inhibitors in BRAF-mutant human melanoma cell lines. Characterization of panels of human cell lines with de novo or acquired resistance to MAPK pathway inhibitors demonstrated that ~50 % of the cell lines exhibited a high OxPhos phenotype. The presence of high OxPhos correlated with increased expression of PGC1-alpha and with sensitivity to combined inhibition of the MAPK pathway and mTORC1/2. mTORC1/2 inhibition caused cytoplasmic sequestration of MITF and subsequent decreased expression of MITF-regulated genes, including PGC1-alpha. In vitro testing demonstrated that a direct OxPhos inhibitor similarly achieved growth inhibition and apoptosis in some human melanoma cell lines with high OxPhos. Further, the OxPhos inhibitor abrogated the growth of inhibitor-resistant BRAF mutant human melanoma cell lines in vivo. Activation of the PI3K/AKT/mTOR pathway by loss of PTEN was also shown to promote resistance to T cell mediated cell killing in vitro and in vivo. Loss of PTEN correlated with decreased CD8 cell infiltrates in clinical specimens and increased expression of immunosuppressive cytokines. While pan-PI3K inhibitors inhibited immune cell viability and function, isoform-selective inhibitors did not significantly affect immune function and they produced synergy with immunotherapy.

**Conclusions:** The PI3K/AKT/mTOR signaling pathway is an important regulator of key cellular processes in melanoma, and should be considered as a candidate combinatorial partner for both targeted and immune therapies.

##### K2 Non-mutational adaptive changes in melanoma cells exposed to BRAF and MEK inhibitors help the establishment of drug resistance

###### Gennaro Ciliberto^1^, Luigi Fattore^1^, Debora Malpicci^2^, Luigi Aurisicchio^3^, Paolo Antonio Ascierto^1^, Carlo M. Croce^4^, Rita Mancini^5,6^

####### ^1^Istituto Nazionale per lo Studio e la Cura dei Tumori “Fondazione G. Pascale”, Naples, Italy; ^2^Dipartimento di Medicina Sperimentale e Clinica, Università degli Studi di Catanzaro “Magna Graecia”, Catanzaro, Italy; ^3^Takis s.r.l., Rome, Italy; ^4^Department of Molecular Virology, Immunology and Medical Genetics, The Ohio State University Comprehensive Cancer Center, Columbus, OH, USA; ^5^Dipartimento di Chirurgia “P. Valdoni”, Sapienza Università di Roma, Rome, Italy; ^6^Dipartimento di Medicina Clinica e Molecolare, Sapienza Università di Roma, Rome, Italy

######## **Correspondence:** Gennaro Ciliberto

*Journal of Translational Medicine* 2016, **14(Suppl 1)**:K2

**Background:** Approximately 50 % of melanoma patients present activating mutations in the BRAF oncogene. For these patients new therapeutic options have been made available in recent years represented by BRAF and MEK kinase inhibitors (KIs). However, the main issue remains the development of drug resistance, which is responsible for disease relapse within months after treatment. Established drug resistance is caused in most cases by several gene mutations which either reactivate the MAPK/ERK pathway or involve activation of receptor tyrosine kinase driven survival pathways. However, besides mutational events it is emerging that a wealth of adaptive changes take place in melanoma cells after drug exposure which heavily contribute to the development of resistance. Our group has focused its attention over the past years to some of these adaptive changes in the search of new therapeutic targets.

**Materials and methods:** Up to ten melanoma cell lines bearing different mutations in the BRAF gene were exposed to BRAFi and/or MEKi from 2 to 72 h and harvested for protein and RNA extraction. Drug resistant melanoma cell populations were selected using increasing drug concentrations over a 2 months period. Activation of RTKs and intracellular signaling was assesses by RTK arrays and western blotting. ErbB3 receptor was inhibited by monoclonal antibodies A3 and A4. Changes in miRNA levels were detected by Nanostring technology or by RT-PCR. Tumor biopsies were obtained from patients before KI therapy or after relapse and used for RNA extraction.

**Results:** We first showed that ErbB3 is the main receptor to be phosphorylated after short term exposure to KIs in different melanoma lines. ErbB3 activation and in turn AKT phosphorylation depends on an autocrine loop due to increased neuregulin-1 production. Anti-ErbB3 mAbs cause receptor internalization and degradation and potentiate the growth inhibitory effect of BRAFi and MEKi in vitro and in vivo. Most importantly mAbs-induced ErbB3 inhibition impairs the development of resistance in vitro and tumor relapse in vivo after therapy discontinuation. More recently we focused our attention on microRNA deregulations after drug exposure of melanoma cells. Our preliminary data show the involvement of a network of miRNAs on the modulation of the response to KIs.

**Conclusions:** Melanoma cells bearing activating BRAF mutations undergo a series of adaptive changes after exposure to drugs that inhibit the MAPK pathway, which help them to survive and help the development resistance. Identification of these adaptive changes is leading to identification of new targets for intervention.

##### K3 Tumor-intrinsic beta-catenin signaling mediates tumor-immune avoidance

###### Stefani Spranger^1^, Thomas F. Gajewski^1,2^

####### ^1^Department of Pathology, University of Chicago, Chicago, IL, USA; ^2^Department of Medicine, University of Chicago, Chicago, IL, USA

######## **Correspondence:** Stefani Spranger

*Journal of Translational Medicine* 2016, **14(Suppl 1)**:K3

**Background:** Growing evidence has emerged that a subset of melanoma patients shows signs for spontaneous anti-tumor immune responses and T cell infiltration into tumor sites, which represents an important prognostic value and which is associated with clinical responses to immunotherapies. However, the underlying molecular mechanisms that can explain the absence of a T cell response in the majority of patients are not defined.

**Results:** Exome sequencing and gene expression profiling of melanoma biopsies revealed activation of β-catenin in a major subset of non-T cell-infiltrated tumors. Using an inducible autochthonous mouse melanoma model (Braf^V600E^/PTEN^−/−^ ± CAT-STA^+^; BP and BPC) we herein demonstrate a causal effect between tumor-intrinsic active β-catenin signaling and T cell exclusion. Mechanistic studies revealed a lack of T cell priming against tumor-associated antigens in the context of β-catenin-expressing tumors. In-depth analysis indicated that absence of T cells was caused by defective recruitment of CD103^+^ dermal dendritic cells, due to repressed expression of the chemokine CCL4. Tumors expressing active β-catenin were resistant to therapy with anti-CTLA-4/anti-PD-L1 antibodies, mimicking the phenotype observed in humans. The absence of CD103^+^ dendritic cells led to defective early T cell priming and absence of systemic immunity. However, whether tumor-intrinsic β-catenin signaling is responsible for mediating tumor resistance even after an anti-tumor T cell response is established remains elusive. To test this notion, we used the spontaneously rejected tumor cell line MC57.SIY, which is known to induce an immunologic memory mediating immune surveillance. Following rejection of MC57.SIY in BP-SIY and BPC-SIY autochthonous tumors were induced. Although the primary SIY-specific CD8^+^ T cell response and the induced memory response were comparable between both tumor models, tumor protection was observed against BP-SIY tumors, whereas it was absent in BPC-SIY tumors. This increased tumor control in BP-SIY mice was accompanied by strong T cell infiltration and a boosted memory response. These results suggest that tumor-intrinsic β-catenin signaling might also be responsible for the observed exclusion of migrating effector T cells into the tumor site.

**Conclusion:** Taken together, these data provide strong evidence that up-regulation of β-catenin in tumor cells is a very potent mechanism of immune evasion against not only a primary immune response, but also against an immunologic memory. Moreover, tumor-intrinsic β-catenin activation likely mediates resistance not only to checkpoint blockade therapy but also to T cell adoptive transfer. Future studies will focus on therapeutic solutions targeting the activated β-catenin pathway with the intention to allow inflammation into this subset of tumors.

##### K4 Intracellular tumor antigens as a source of targets of antibody-based immunotherapy of melanoma

###### Yangyang Wang, Soldano Ferrone

####### Department of Surgery, Massachusetts General Hospital, Harvard Medical School, Boston, MA, USA

######## **Correspondence:** Soldano Ferrone

*Journal of Translational Medicine* 2016, **14(Suppl 1)**:K4

It is generally assumed that in order to be used as targets of antibody- based immunotherapy of malignant diseases, tumor antigens have to be expressed on the plasma membrane of tumor cells. This assumption has markedly reduced the number of tumor antigens which can be targeted with antibody-based immunotherapy, since only a few of the molecules expressed on the plasma membrane of tumor cells meet the criteria required to be used as targets of antibody-based immunotherapy. Intracellular TAs which analyses with T cells have shown to be much more diverse than those expressed on plasma membrane, have been excluded from studies to identify targets of antibody-based immunotherapy for the treatment of malignant diseases.

We have hypothesized that, under certain experimental conditions and especially in malignant cells, intracellular molecules may migrate to cell surface and become accessible to interact with the corresponding antibodies. Our hypothesis is supported by the isolation of the human monoclonal antibody (mAb) W9, which recognizes an extracellular epitope of the intracellular chaperone molecule glucose regulated protein of 94,000 Da (Grp94). The mAb W9 defined Grp94 epitope is expressed on various types of malignant cells including melanoma. In several types of cancer, the mAb W9 defined Grp94 epitope is expressed not only on differentiated cancer cells, but also on cancer initiating cells (CICs). The mAb W9 defined Grp94 epitope is upregulated on BRAF^V600E^ melanoma cells by incubation with BRAF inhibitor, and with chemotherapeutic agents. In contrast, the mAb W9 defined Grp94 epitope is not detectable on normal cells. Therefore it represents an attractive target to implement antibody-based immunotherapy of various types of solid tumors.

In this study we have investigated the in vitro and in vivo anti-tumor activity of mAb W9 utilizing malignant melanoma as a model. The results obtained indicate that mAb W9 inhibits the growth of melanoma cells. This effect reflects the induction of apoptosis as well as the inhibition of several signaling pathways including Erk, Akt and FAK pathways. The anti-tumor activity of mAb W9 is enhanced by the small molecule LDE225, an inhibitor of SHH pathway. Furthermore, mAb W9 delays the development of BRAF inhibitor resistance in melanoma cells with mutant BRAF. Lastly, mAb W9 induces the regression of experimental lung metastasis established in immunodeficient mice by intravenous injection of melanoma M21 cells.

If representative of a general phenomenon, these results suggest that intracellular tumor antigens may be a useful source of targets for antibody-based immunotherapy of melanoma and other types of solid tumors.

#### Combination therapies

##### K5 Harnessing radiotherapy to improve responses to immunotherapy in cancer

###### Claire Vanpouille-Box^1^, Erik Wennerberg^1^, Karsten A. Pilones^1,2^, Silvia C. Formenti^3^, Sandra Demaria^1,2^

####### ^1^Department of Pathology, NYU School of Medicine, New York, NY, USA; ^2^Department of Radiation Oncology, NYU School of Medicine; New York, NY, USA;^3^Department of Radiation Oncology, Weill Cornell Medical College, New York, NY, USA

######## **Correspondence:** Sandra Demaria

*Journal of Translational Medicine* 2016, **14(Suppl 1)**:K5

Recent successes of cancer immunotherapy have demonstrated the power of anti-tumor T cells to change outcome of metastatic disease and are transforming cancer treatment. Two key observations have emerged from clinical studies. The first is that a fraction of patients with metastatic disease possesses a sufficient number and repertoire of tumor-reactive T cells that can be unleashed by immune checkpoint blockade to cause tumor regression [1]. The second is that unique mutated proteins expressed by an individual tumor are a source of powerful tumor-specific T cell antigens [2]. An outstanding challenge is to devise novel strategies to vaccinate the vast majority of cancer patients who do not respond to immune checkpoint blockade against their own individual tumor.

Local tumor radiotherapy is increasingly considered an appealing strategy to achieve personalized vaccination, and we have shown that it can convert tumors refractory to immune checkpoint inhibitors into responsive ones by breaking down the barriers to immune cell infiltration and promoting development of anti-tumor T cells [3–5]. These pro-immunogenic effects of radiation are modulated by the pre-existing tumor microenvironment, and are dependent on the dose and fractionation of radiation employed [6, 7]. Immunogenic cell death is induced by radiation in a dose-dependent way, with higher ablative single doses being more effective in vitro [8]. However, in vivo the pro-immunogenic effects of radiation are countered by generation of immunosuppressive mediators. We have recently identified a critical role of transforming growth factor beta (TGFβ) in regulating development of anti-tumor T cells and tumor rejection following radiotherapy [9]. We are currently investigating the role of other TGFβ family members, as well as adenosinergic pathways in regulating the shift between immune activation and immune suppression in the tumor following radiotherapy. The overarching goal of our work is to define radiation interactions with the microenvironment of a given tumor, and devise a personalized approach to optimize in situ vaccination by radiation. Results of these studies will improve the design of clinical trials testing radiation in combination with immune checkpoint inhibitors and other immunotherapies.

References

1. Sharma P, Allison JP. The future of immune checkpoint therapy. Science. 2015;348(6230):56–61.

2. Schumacher TN, Schreiber RD. Neoantigens in cancer immunotherapy. Science 2015;348(6230):69–74.

3. Demaria S, Kawashima N, Yang AM, et al. Immune-mediated inhibition of metastases following treatment with local radiation and CTLA-4 blockade in a mouse model of breast cancer. Clin Cancer Res. 2005;11:728–34.

4. Pilones KA, Vanpouille-Box C, Demaria S. Combination of radiotherapy and immune checkpoint inhibitors. Semin Radiat Oncol. 2015;25(1):28–33.

5. Golden EB, Demaria S, Schiff PB, et al. An abscopal response to radiation and ipilimumab in a patient with metastatic non-small cell lung cancer. Cancer Immunol Res. 2013;1(6):365–72.

6. Dewan MZ, Galloway AE, Kawashima N, et al. Fractionated but not single dose radiotherapy induces an immune-mediated abscopal effect when combined with anti-CTLA-4 antibody. Clin Cancer Res. 2009;15(17):5379–88.

7. Demaria S, Formenti SC. Radiation as an immunological adjuvant: current evidence on dose and fractionation. Front Oncol. 2012;2:153.

8. Golden EB, Frances D, Pellicciotta I, et al. Radiation fosters dose-dependent and chemotherapy-induced immunogenic cell death. OncoImmunology. 2014;3:e28518.

9. Vanpouille-Box C, Diamond JM, Pilones KA, et al. TGFβ is a master regulator of radiation therapy-induced anti-tumor immunity. Cancer Res. 2015;75(11): 2232–42.

##### K6 Creating a T cell-inflamed tumor microenvironment overcomes resistance to checkpoint blockade

###### Haidong Tang, Yang Wang, Yang-Xin Fu

####### Department of Pathology, University of Texas Southwestern, Dallas, USA

######## **Correspondence:** Yang-Xin Fu

*Journal of Translational Medicine* 2016, **14(Suppl 1)**:K6

Immune checkpoint blockade results in impressive clinical responses. However, only a minority of patients benefit from such therapies. It is urgently desired to understand the mechanisms, and more importantly, to find approaches that can overcome tumor resistance to checkpoint blockade. Several clinical studies have shown strong correlations between a spontaneous T cell-inflamed tumor microenvironment and responses to checkpoint blockade. However, majority of established tumors are non-T cell-inflamed and resist to such immunotherapies. It raises possibility that preexisting T cells inside tumor tissues is essential for checkpoint blockade. Here we demonstrate that sufficient T cell infiltration in tumor tissues is a prerequisite for the response to PD-L1 blockade. Targeting tumors with tumor necrosis factor superfamily member LIGHT activates lymphotoxin beta receptor signaling, leading to the production of chemokines that recruit massive numbers of T cells. Furthermore, targeting non-T cell-inflamed tumor tissues by antibody-guided LIGHT creates a T cell-inflamed microenvironment and overcomes tumor resistance to checkpoint blockade. Our study indicates that targeting tumor with LIGHT may be an effective strategy for non-T cell-inflamed tumors which are resistant to checkpoint blockades.

##### K7 Biomarkers for treatment decisions?

###### Reinhard Dummer

####### Department of Dermatology, University of Zurich Hospital, Zurich, Switzerland

######## **Correspondence:** Reinhard Dummer

*Journal of Translational Medicine* 2016, **14(Suppl 1)**:K7

Melanoma includes a group of genetically and clinically district malignancies derived from the neural crest. UV-light contributes significantly. It is the major cause for genetical alterations. The mutation load is also relevant for immune responses. In addition, there are a number of driver mutations that can be targeted by small molecules. In this context melanoma is a paradigm for precision medicine. Biomarkers are essential in this context and will get more and more important for clinical treatment decisions.

##### K8 Combining oncolytic therapies in the era of checkpoint inhibitors

###### Igor Puzanov

####### Vanderbilt-Ingram Cancer Center, Vanderbilt University, Nashville, TN, USA

######## **Correspondence:** Igor Puzanov

*Journal of Translational Medicine* 2016, **14(Suppl 1)**:K8

**Background**: T-VEC is an HSV-derived oncolytic immunotherapy designed to induce systemic antitumor immunity. In a phase 3 melanoma study, T-VEC monotherapy demonstrated a significantly higher durable response rate (DRR, ≥6 months response) vs GM-CSF (Andtbacka et al. ASCO 2013). Combining T-VEC to promote release of tumor-derived antigens with an immune checkpoint inhibitor such as ipi to improve T cell responses may enhance efficacy compared to either alone. The phase 1b portion of this phase 1b/2 combination study (NCT01740297) completed enrollment and met its primary objective with no dose limiting toxicities and objective response rate (ORR) of 56 % (Puzanov et al. ASCO 2014).

**Materials and methods:** Key criteria: Stage IIIB-IV melanoma, no prior systemic tx, measurable disease, and ≥1 injectable cutaneous, subcutaneous, or nodal lesion. T-VEC was given intralesionally at ≤4 mL of 10^6^ PFU/mL at week 1, then 10^8^ PFU/mL at week 4 and then q2w. Ipi 3 mg/kg q3w was given as four infusions starting week 6. T-VEC continued until DLT, intolerance, all injectable tumors disappeared, or disease progression (PD) per immune-related response criteria (irRC). Blood was collected at weeks 1, 4, 6, 9, 15, and after tx completion and tumor assessments Q12W.

**Results:** Data cutoff was 22 Dec 2014 with all pts ≥17 months from start of tx. Of 18 pts receiving T-VEC + ipi, treatment-emergent gr 3/4 AEs occurred in 32 % and gr 3/4 immune-related AEs in two pts with no treatment-related deaths. Per irRC, ORR: 56 % (33 % CR), DRR: 44 %. Median time to response: 5.3 months (range 2.6–5.7). Median progression free survival (PFS): 10.6 months (2.6−19.3+). Median overall survival (OS) was not reached with 12 and 18 months survival at 72.2 and 67 %. On a lesion level, 24 and 11 of 35 injected index lesions and 8 and 5 of 16 uninjected index lesions regressed ≥50 and 100 %, respectively. Higher antibody (ab) titers to MAGEA3 occurred more frequently during tx in responders, but ab to 27 other melanoma-associated antigens showed less distinct changes.

**Conclusions:** At >17 months, TVEC + ipi continued to demonstrate durable responses with 2/3 of pts alive at 18 months and no new safety signals. Phase 2 (ipi vs T-VEC + ipi) is ongoing and Phase Ib/III study of T-VEC + pembrolizumab has been started.

##### K9 Immune checkpoint blockade for melanoma: should we combine or sequence ipilimumab and PD-1 antibody therapy?

###### Michael A. Postow

####### Memorial Sloan Kettering Cancer Center, New York, USA

######## **Correspondence:** Michael A. Postow

*Journal of Translational Medicine* 2016, **14(Suppl 1)**:K9

Immune checkpoint blockade with agents such as ipilimumab, pembrolizumab, and nivolumab has dramatically changed the outlook for patients with metastatic melanoma. Several studies have investigated the combination of various checkpoint blocking antibodies such as the combination of ipilimumab and nivolumab and the combination of ipilimumab and pembrolizumab. We await overall survival data from phase III studies to determine the true values of combining immune checkpoint inhibitors. Nonetheless, combinations have demonstrated impressive objective response rates and progression free survival. In this talk, we will discuss the available data for combining immune checkpoint inhibitors, side effects, and future directions.

#### News in immunotherapy

##### K10 An update on adjuvant and neoadjuvant therapy for melanoma

###### Ahmad Tarhini

####### Melanoma and Skin Cancer Program, University of Pittsburgh Cancer Institute, Pittsburgh, PA, USA

######## **Correspondence:** Ahmad Tarhini

*Journal of Translational Medicine* 2016, **14(Suppl 1)**:K10

Patients with Stages IIB-C/III/IV melanoma that surgically resectable carry a high risk for melanoma recurrence and death from melanoma with surgical management alone. Systemic adjuvant therapy of targets melanoma micrometastases and is indicated postoperatively where it may provide the greatest opportunity for cure before relapse into advanced inoperable stages. Multiple systemic therapeutic agents have been tested as adjuvant therapy for melanoma with durable benefits seen with interferon-α (IFN). In randomized clinical trials, IFNα has been tested as part of various regimens that vary by dose, duration, route of administration and formulation. Several randomized trials and three major meta-analyses have demonstrated a reproducible and significant impact on relapse free survival (RFS). Overall survival benefit was seen only in two of the three ECOG and U.S. Intergroup trials that tested the 1 year high dose regimen as compared to observation (E1684) and the GMK vaccine (E1694). Pegylated IFN was shown to improve RFS as tested in the EORTC 18,991 trial. Recently reported adjuvant trials include the phase III EORTC 18,071 trial that tested ipilimumab at 10 mg/kg versus placebo and has met it primary endpoint of RFS. The U.S. Intergroup E1609 trial is expected to inform the field on the clinical efficacy of ipilimumab versus interferon alfa-2b in the high-risk adjuvant setting, as well as the risk-to-benefit ratio of the two ipilimumab dosages (3 and 10 mg/kg) tested. Trials testing adjuvant therapy with the anti-PD1 antibodies pembrolizumab and nivolumab are ongoing. Neoadjuvant studies are also ongoing and carry significant promise.

##### K11 Targeting multiple inhibitory receptors in melanoma

###### Joe-Marc Chauvin^1,2^, Ornella Pagliano^1,2^, Julien Fourcade^1,2^, Zhaojun Sun^1,2^, Hong Wang^3^, Cindy Sanders^1,2^, John M. Kirkwood^1,2^, Tseng-hui Timothy Chen^4^, Mark Maurer^4^, Alan J. Korman^4^, Hassane M. Zarour^1,2^

####### ^1^Department of Medicine and Division of Hematology/Oncology, University of Pittsburgh, School of Medicine, Pittsburgh, PA, 15213, USA; ^2^Department of Immunology, University of Pittsburgh, School of Medicine, Pittsburgh, PA, 15213, USA; ^3^Department of Biostatistics, University of Pittsburgh, School of Medicine, Pittsburgh, PA, 15213, USA; ^4^Biologics Discovery California, Bristol-Myers Squibb, Redwood City, CA, 94063, USA

######## **Correspondence:** Hassane M. Zarour

*Journal of Translational Medicine* 2016, **14(Suppl 1)**:K11

**Background:** Multiple mechanisms of melanoma-induced immune escape contribute to the failure of the spontaneous or vaccine-induced immune T cell responses to promote tumor regression in humans. In particular, a number of inhibitory pathways play a critical role in impeding T cell responses to tumor antigens, including PD-1, Tim-3 and TIGIT.

**Results:** Our findings show that PD-1, Tim-3 and TIGIT are upregulated by CD8^+^ and CD4^+^ T cells at the periphery and at tumor sites in patients with metastatic melanoma. We observed that melanoma cells and antigen presenting cells in metastatic melanoma express the PD-1, Tim-3 and TIGIT inhibitory ligands. Dual PD-1/Tim-3 and dual PD-1/TIGIT blockades enhanced the expansion and function of TA-specific CD8^+^ T cells isolated from patients with advanced melanoma. Interestingly, CD8^+^ TILs downregulated CD226, the costimulatory counterpart to TIGIT, supporting an imbalance of TIGIT/CD226 expression in metastatic melanoma, which may contribute to melanoma-induced T cell dysfunction.

**Conclusion:** Collectively, our findings present the rationale for targeting multiple inhibitory pathways including PD-1, Tim-3 and TIGIT to better counteract melanoma-induced cell dysfunction and improve melanoma patients ‘clinical outcome.

##### K12 Improving adoptive immune therapy using genetically engineered T cells

###### David F. Stroncek

####### Cell Processing Section, Department of Transfusion Medicine, Clinical Center, National Institutes of Health, Bethesda, MD, USA

######## **Correspondence:** David F. Stroncek

*Journal of Translational Medicine* 2016, **14(Suppl 1)**:K12

T cells engineered to express high affinity T cell receptors or chimeric antigen receptors (CAR) have been successful for treating cancer and hematological malignancies. We are using anti-CD19 and anti-CD22 CAR T cells to treat patients with acute lymphocytic leukemia and B cell lymphomas. While early phase clinical studies with these therapies have been promising, occasionally, the CAR T cells fail to expand in culture resulting in inadequate doses of transduced cells for effective therapy. The poor expansion of the T cells appears to be related contamination of the autologous leukocyte apheresis products used as starting material with large quantities of monocytes or granulocytes. We have been evaluating manufacturing methods that make use of lymphocyte enrichment prior to initiating the T cell culture. The methods include plastic adherence, elutriation and CD4/CD8 cell selection. Another limitation of CAR T cell therapy is their limited in vivo survival. In order to improve the in vivo survival and expansion of CAR T cells a GMP method has been developed to isolate, transduce and expand stem memory T cells (Tscm). CD19 CAR Tscm will soon be tested in a clinical trial which will treat adults with B cell malignancies. Limited survival of genetically engineered T cells may also be due to immune reactions to the single chain variable portion of the mouse monoclonal antibody used in the vector. Investigators at our institution have developed a lentiviral vector with humanized anti-CD19 for CAR T cell therapy.

#### Tumor microenvironment and biomarkers

##### K13 Myeloid cells and tumor exosomes: a crosstalk for assessing immunosuppression?

###### Veronica Huber, Licia Rivoltini

####### Fondazione IRCCS Istituto Nazionale dei Tumori, Milan, Italy

######## **Correspondence:** Licia Rivoltini

*Journal of Translational Medicine* 2016, **14(Suppl 1)**:K13

**Background:** Exosomes are endosome-derived nanovesicles involved in intercellar cross-talk through the transfer of proteins and genetic material. Accumulating data suggests that exosomes released from tumor cells may play an essential role in modulating host microenvironment, conditioning tumor immunity both at local and systemic level. We have been collecting evidence that these nanovesicles exert a broad array of detrimental effects on the immune system, such as impairment of monocyte differentiation into dendritic cells and induction of suppressive effectors.

**Materials and methods:** An in vitro model on MDSC conversion from normal monocytes by tumor exosomes (Exo-MDSC) was developed and used to study the molecular pathways involved in MDSC generation. Gene-expression and miRNA profiling of exo-MDSC, blood myeloid cells and plasma exosomes isolated from melanoma patients, and in vitro studies with antagonists and mimics, were applied to identify the miRNAs responsible for exosome-MDSC cross-talk in vitro and in vivo.

**Results:** Compared to untreated monocytes, Exo-MDSC release higher levels of protumorigenic and immunosuppressive cyto/chemokines, down-modulate the expression of HLA-DR and acquire the ability to inhibit T cell proliferation. A gene-expression signature, involving CCL2, IL6 and PD1L, and a defined miRNA profiling have been identified in Exo-MDSC. Using miRNA mimics and inhibitors we demonstrated a strong impact on the regulation of HLA-DR, IL6 and CCL2 for four candidate miRNA, suggesting their involvement in MDSC differentiation. Furthermore, the silencing of these miRNA in melanoma cell lines abrogates exosome conversion potential, sustaining the hypothesis of miRNA transfer via exosomes. Importantly, peripheral blood monocytes from melanoma patients share defined traits of Exo-MDSC gene-expression and miRNA signature, in addition to phenotypic and functional features. In addition, extracellular vesicles isolated from plasma of melanoma patients, but not from healthy donors, induce MDSC conversion ex vivo, and show a higher concentration of myelo-conditioning miRNA.

**Conclusions:** Tumor exosomes are involved in triggering MDSC conversion by directly transferring myelo-active miRNAs to myeloid cells. Since MDSC accumulate in peripheral blood of tumor patients in association with disease progression, the identification of exosomes and myelo-conditioning miRNA paves the way for the development of novel immune-based therapeutic strategies and prognostic biomarkers in cancer.

Supported by Italian Association For Cancer Research—AIRC Grant code 12162

##### K14 Update on the SITC biomarker taskforce: progress and challenges

###### Magdalena Thurin

####### Cancer Diagnosis Program, Division of Cancer Treatment and Diagnosis, NCI, NIH, Rockville, MD, USA

######## **Correspondence:** Magdalena Thurin

*Journal of Translational Medicine* 2016, **14(Suppl 1)**:K14

Advances in cancer immunotherapy, including positive results from clinical trials testing new agents and combinations become applicable and effective treatment for many common and deadly cancers. However, a critical limitation in cancer therapy remains the identification of patients likely to respond to a given immunotherapy who could be stratified for treatment. The Society for Immunotherapy of Cancer (SITC) organized workshops and working groups in the past that focused on immune prognostic and predictive biomarkers and immunologic monitoring (1–2). SITC Taskforce on Immunotherapy Biomarkers developed a series of recommendations and resources for the field (3–4).

Although, the field still lacks standardized and validated biomarkers to identify the patients who are responding to a treatment specific immunotherapies and who will have minimal or acceptable toxicities there are, many promising candidate assays, novel technologies and molecules. Importantly, companion diagnostic assay for response in non-small cell lung cancer have been recently approved by the FDA for patients selection for immunotherapy (PD-L1 IHC 22C3 pharmDx).

In light of these developments SITC Immune Biomarkers Taskforce has reconvened to review the state of the art, identify current hurdles to further success and to make recommendations to the field. Topics being addressed by individual working groups include: (1) validation of candidate biomarkers, (2) identification of the most promising technologies, (3) testing of high throughput immune signatures and (4) investigation of the pre-treatment tumor microenvironment. Resultant recommendations will be published in JITC and discussed during the workshop in April 2016.

Specific focus of the talk will be an update from the activity of working group 1 (WG1), led by Drs. Magdalena Thurin and Giuseppe Masucci, focusing on immunologic assays’ standardization and validation. This group includes experts from the US National Cancer Institute (NCI), academia, the US FDA, biotech and pharma. They are developing roadmaps for standardization and validation of specific assays that are currently seen as the most promising, particularly with companion diagnostics as the goal. They are also examining proficiency panels of assays, and “integral” and “integrated” (versus exploratory) biomarkers in clinical trials, and statistical designs for biomarker validation.

References

1. Keilholz U, Weber J, Finke JH, Gabrilovich DI, Kast WM, Disis ML, et al. Immunologic monitoring of cancer vaccine therapy: results of a workshop sponsored by the Society for Biological Therapy. J Immunother. 2002;25:97–138.

2. Lotze MT, Wang E, Marincola FM, Hanna N, Bugelski PJ, Burns CA, et al. Workshop on cancer biometrics: identifying biomarkers and surrogates of cancer in patients: a meeting held at the Masur Auditorium, National Institutes of Health. J Immunother. 2005;28:79–119.

3. Butterfield LH, Disis ML, Fox BA, Lee PP, Khleif SN, Thurin M, et al. A systematic approach to biomarker discovery; preamble to “the iSBTc-FDA taskforce on immunotherapy biomarkers”. J Transl Med. 2008;6:81.

4. Butterfield LH, Palucka AK, Britten CM, Dhodapkar MV, Håkansson L, Janetzki S, et al. Recommendations from the iSBTc-SITC/FDA/NCI Workshop on Immunotherapy Biomarkers. Clin Cancer Res. 2011;17:3064–76.

#### World-wide immunoscore task force: an update

##### K15 The immunoscore in colorectal cancer highlights the importance of digital scoring systems in surgical pathology

###### Tilman Rau, Alessandro Lugli

####### Institute of Pathology, University of Bern, Bern, Switzerland

######## **Correspondence:** Tilman Rau

*Journal of Translational Medicine* 2016, **14(Suppl 1)**:K15

**Background:** The TNM classification from the UICC/AJCC and the guidelines of the WHO 2010 are the basis for the classification of colorectal cancer (CRC). Three clinical scenarios still require novel, reproducible and standardized biomarkers: malignant polyps, stage II CRC with adverse prognosis and the pre-operative management of colon and rectal cancer patients. The immunoscore using immune markers CD3 and CD8 is a promising prognostic approach based on immunohistochemistry and a digital scoring system. Nevertheless, there is still the challenge to implement the immunoscore in daily diagnostic practice in surgical pathology. The aim of this study is to present the results experienced at our Institute of Pathology as one of the participating centers.

**Materials and methods:** The immunoscore (CD3 and CD8) was performed on 331 well characterized CRC patients including the TNM classification, L and V stage, perineuronal infiltration, histological subtype, tumor grade, peritumoral inflammation, mismatch repair status, overall survival and information on adjuvant radio- and chemotherapy using the Roche Benchmark II Ultra and the Definiens Software for the digital pathology analysis. All the tests were performed at the Translational Research Unit (TRU) of the Institute of Pathology, University of Bern.

**Results:** The total turn-around time (TAT) for one case is approximately 15 h including 12 h for immunostaining, 10–20 min for scanning, 30 min for definition of the tumor area and 2 h for calculation. These results demand a well defined working process. Therefore, we elaborated a possible work flow approach based on the LEAN management system: phase 1: CRC diagnosis, block selection and digital order of CD3 and CD8 by the pathologist; phase 2: immunohistochemical staining and scanning by the immunohistochemistry laboratory; phase 3: definition of the tumor area, calculation and reporting of the results by the pathologist; phase 4: discussion of the results at the interdisciplinary CRC board.

**Conclusions:** The implementation of the immunoscore in surgical pathology on the operative level needs a well defined strategy which is simple, applicable and user-friendly. Therefore, based on our experience well defined working processes based on SOPs and TATs in the surgical pathology divisions and digital pathology development units which test novel and promising digital scoring systems before the implementation in the daily diagnostic practice should be considered.

##### K16 The immunoscore: toward an integrated immunomonitoring from the diagnosis to the follow up of cancer’s patients

###### Franck Pagès^1,2^

####### ^1^Laboratory of Immunology. Hopital Européen Georges Pompidou, Paris, France; ^2^INSERM team 15, Cordeliers Research Center, Paris, France

######## **Correspondence:** Franck Pagès

*Journal of Translational Medicine* 2016, **14(Suppl 1)**:K16

We provided evidence that the type, the density and the location of immune cells within colonic tumor strongly influence the prognosis, independently of the TNM classification [1–4]. Thus, the adaptive immune reaction composed of T lymphocytes (CD3) with cytotoxic (CD8) and memory (CD45RO) phenotype within the core of the tumor (CT) and the invasive margin (IM) is a highly significant parameter to predict recurrence and survival. Multivariate analyses have revealed that T cell–immune parameters not only remain meaningful when combined with the TNM staging system but also were shown to be more accurate in determining prognosis than are conventional clinicopathological criteria. These data strongly suggests that tumor behavior should now be considered as the result of a balance between the invasive tumor process and the response of the host of which the local immune reaction is a major component. We established a methodology named “Immunoscore” to assess in clinical practice the immune infiltrate. Each tumor is categorized into Hi or Lo density for each marker in each tumor region, according to a predetermined cut-off value using the minimum P value approach. Patients are stratified according to a score ranging from I0 to I4 depending on the total number of high densities observed (two markers assessed in CT, two markers assessed in IM). For example, I4 refers to a tumor with high densities of CD3 and CD8 cells in CT and IM regions. We recently applied this classification in patients with clinically localized rectal cancer treated by primary surgery [5] and showed that the Immunoscore could be a useful prognostic marker. Cox multivariate analysis supports the advantage of the Immunoscore compared with the tumor–node–metastasis (TNM) staging in predicting recurrence and survival and the lymph node ratio (LNR) added information in the prognostic model. The information carried by the LNR could reflect complementary aspects of the antitumor immunity not depicted by the Immunoscore. In an effort to promote use of the Immunoscore in routine clinical settings, an international task force was initiated, supported by the Society for Immunotherapy of Cancer (SITC) [6]. An independent international panel of expert laboratories has started to work on large retrospective cohorts of colonic cancers (TNM stage I-III) to test the Immunoscore in routine clinical settings. The availability of the Immunoscore could significantly improve the prognostic assessment of patients and better guide the therapeutic decision, and possibly choosing patients likely to benefit from immunotherapy.

References

1. Galon J, Costes A, Sanchez-Cabo F, Kirilovsky A, Mlecnik B, Lagorce-Pages C, Tosolini M, Camus M, Berger A, Wind P, et al. Type, density, and location of immune cells within human colorectal tumors predict clinical outcome. Science. 2006;313:1960–64.

2. Pagès F, Berger A, Camus M, Sanchez-Cabo F, Costes A, Molidor R, Mlecnik B, Kirilovsky A, Nilsson M, Damotte D, et al. Effector memory T cells, early metastasis, and survival in colorectal cancer. N Engl J Med. 2005;353(25):2654–66.

3. Pages F, Kirilovsky A, Mlecnik B, Asslaber M, Tosolini M, Bindea G, Lagorce C, Wind P, Marliot F, Bruneval P, et al. In situ cytotoxic and memory T cells predict outcome in patients with early-stage colorectal cancer. J Clin Oncol. 2009;27:5944–51.

4. Mlecnik B, Tosolini M, Kirilovsky A, Berger A, Bindea G, Meatchi T, Bruneval P, Trajanoski Z, Fridman WH, Pagès F, et al. Histopathologic-based prognostic factors of colorectal cancers are associated with the state of the local immune reaction. J Clin Oncol. 2011;29(6):610–18.

5. Anitei MG, Zeitoun G, Mlecnik B, Marliot F, Haicheur N, Todosi AM, Kirilovsky A, Lagorce C, Bindea G, Ferariu D, et al. Prognostic and predictive values of the immunoscore in patients with rectal cancer. Clin Cancer Res. 2014;20(7):1891–99.

6. Galon J, Mlecnik B, Bindea G, Angell HK, Berger A, Lagorce C, Lugli A, Zlobec I, Hartmann A, Bifulco C, et al. Towards the introduction of the ‘Immunoscore’ in the classification of malignant tumours. J Pathol. 2014;232(2):199–209.

#### Economic sustainability of melanoma treatments: regulatory, health technology assessment and market access issues

##### K17 Nivolumab, the regulatory experience in immunotherapy

###### Jorge Camarero^1^, Arantxa Sancho^2^

####### ^1^Oncology Area, Spanish Medicines Agency and Medical Devices (AEMPS), Madrid, Spain; ^2^Research Institute Hospital Puerta de Hierro Majadahonda, Madrid, Spain

######## **Correspondence:** Jorge Camarero

*Journal of Translational Medicine* 2016, **14(Suppl 1)**:K17

**Background:** Each year in Europe, 62,000 new cases of melanoma are diagnosed. DTIC was considered the Standard of Care until the appearance of two new classes of compounds in the treatment of metastatic melanoma. These drugs were a CTLA-4 blocking antibody and the therapies targeting the BRAF kinase pathways. Recently, Nivolumab a highly specific programmed death-1 (PD-1) checkpoint inhibitor has been authorised [1]. In this oral communication, the regulatory experience with nivolumab is reviewed.

**Materials and methods:** European public assessment report (a set of documents describing the evaluation of a medicine authorised via the centralised procedure) and the publications of the clinical trials of nivolumab, will be the basis for the review.

**Results:** The background for the authorisation of nivolumab in the treatment of subjects with advanced melanoma were two different studies: study CA209037 [2, 3]: a phase 3 trial of nivolumab vs investigator’s choice in advanced Melanoma patients progressing post anti-CTLA-4 therapy; and Study CA209069 [2, 4]: a phase 3, study of nivolumab vs dacarbazine in subjects with BRAF wild type, previously untreated.

Nivolumab shown convincing statistically significant and clinically relevant results in the first-line treatment of metastatic melanoma. Unfortunately, mature data on OS are not available, since only 34 % of the events have occurred. However, it seems reasonable to assume that the higher life expectancy associated to nivolumab in first line will be maintained.

Regarding the use of nivolumab in last line, after failure of ipilimumab and/or BRAF inhibitors, positive results have been observed for ORR and PFS although limited to a subset of patients. However, the OS curves show an unexpected profile with the lines crossing between 6 and 9 months. It seems plausible that the delay in the onset of the effect of nivolumab (3 months) contributes to the higher rate of deaths in the nivolumab arm in the first 3 months of treatment. This effect has been also observed in the treatment of non-squamous non–small-cell lung cancer (NSCLC) that had progressed during or after platinum-based doublet chemotherapy [5]. On the contrary, the same molecule has shown a clear benefit in survival in squamous NSCLC [6, 7].

**Conclusions:** The use of nivolumab in some patients might mean a worse option than chemotherapy. It remains a challenge to identify those patients who could have a chance to benefit from nivolumab.

References

1. Authorisation details for Opdivo. Available on: http://www.ema.europa.eu/ema/index.jsp?curl=pages/medicines/human/medicines/003985/human_med_001876.jsp&mid=WC0b01ac058001d124 (Retrieved October 23, 2015).

2. European public assessment report for Opdivo. Available on: http://www.ema.europa.eu/docs/en_GB/document_library/EPAR_-_Public_assessment_report/human/003985/WC500189767.pdf (Retrieved October 23, 2015).

3. Robert C, Long GV, Brady B, Dutriaux C, Maio M, Mortier L, et al. Nivolumab in previously untreated melanoma without BRAF mutation. N Engl J Med. 2015;372(4):320–30.

4. Weber JS, D’Angelo SP, Minor D, Hodi FS, Gutzmer R, Neyns B, et al. Nivolumab versus chemotherapy in patients with advanced melanoma who progressed after anti-CTLA-4 treatment (CheckMate 037): a randomised, controlled, open-label, phase 3 trial. Lancet Oncol. 2015;16(4):375–84.

5. Borghaei H, Paz-Ares L, Horn L, Spigel DR, Steins M, Ready NE, et al. Nivolumab versus docetaxel in advanced nonsquamous non-small-cell lung cancer. N Engl J Med. 2015;373(17):1627–39.

6. European public assessment report for nivolumab. Available on: http://www.ema.europa.eu/docs/en_GB/document_library/EPAR_-_Public_assessment_report/human/003840/WC500190651.pdf (Retrieved October 23, 2015).

7. Brahmer J, Reckamp KL, Baas P, Crinò L, Eberhardt WE, Poddubskaya E, et al. Nivolumab versus docetaxel in advanced squamous-cell non-small-cell lung cancer. N Engl J Med. 2015;373(2):123–35.

##### K18 Evidence to optimize access for immunotherapies

###### Claudio Jommi^1,2^

####### ^1^Department of Pharmaceutical Sciences, Università del Piemonte Orientale, Novara, Italy; ^2^Cergas (Centre for Research on Health and Social Care Management), Università Bocconi, Milano, Italy

######## **Correspondence:** Claudio Jommi

*Journal of Translational Medicine* 2016, **14(Suppl 1)**:K18

**Background:** Interest in onco immunotherapies has been growing in last years, due to market launch of ipilimumab for advanced melanoma. Ipilimumab provides important benefits, but is also very costly (1, 2). Its incremental cost-effectiveness is over thresholds and closer to the 50 k Euro per life-year saved threshold if US list prices (3) and European prices (4) are respectively used. NICE recommended ipilimumab with a hidden discount on list price. Drugs pipelines include many new onco immunotherapy. An assessment framework of their value for money is needed for managing their future market access (reimbursement/price/recommendation). The present research aims at providing some pillars of this framework.

**Materials and methods:** The study follows a Delphi methodology, a technique used to facilitate information-gathering on complex issues (5). More specifically, we relied on a e-Delphi (6) organized in three rounds and involving nine experts, including clinicians, health economists and payers. Panelists were asked to comment on ten statements, explaining the extent to which they agreed or disagreed with assertions. Statements were subsequently modified and resubmitted for a final consensus.

**Results:** This paragraph quotes six of the ten statements. The other four specifically concern the Italian context. (i) Medium-long term survival is the most appropriate endpoint to consider for market access of onco immunotherapies. Progression Free Survival and Best Overall Response Rate do not fully express benefits and immuno-related surrogate endpoints still need surrogacy validation. (ii) More specifically, the increase in 2–5 years probability of survival is the most important indicator. Quality of life and adverse events should be also considered. (iii) Market access would be easier managed if methods requirements (endpoints, indicators, follow-up, statistical analysis) are strengthened. (iv) Cost-effectiveness used for market access purposes should rely on robust extrapolation of future survival; results provided by the industry should be validated by independent research groups; incremental cost-effectiveness ratio thresholds should be carefully applied; cost-effectiveness should be integrated with a budget impact analysis. (v) Coverage with evidence development agreements should be implemented, due to benefits long-term and uncertainty: this requires post-approval evidence on survival, quality of life and costs. (vi) Long-term survival predictive factors would be extremely useful to enhance sustainability, provided that they actually reduce uncertainty. Manufacturer funded treatment initiation may be applied for expected non-responders. However clinical research has not found reliable markers for onco immunotherapies yet.

References

1. Jönsson B, Wilking N. Cancer vaccines and immunotherapeutics: challenges for pricing, reimbursement and market access. Hum Vaccin Immunother. 2012;8(9):1360–3.

2. Geynisman DM, Chien CR, Smieliauskas F, et al. Economic evaluation of therapeutic cancer vaccines and immunotherapy: a systematic review. Hum Vaccin Immunother. 2014;10(11):3415–24.

3. Barzey V, Atkins MB, Garrison LP, et al. Ipilimumab in 2nd line treatment of patients with advanced melanoma: a cost-effectiveness analysis. J Med Econ. 2013;16(2):202–12.

4. Radford M, Cortes P, Carrasco J, et al. Cost-effectiveness of ipilimumab in previously treated patients for advanced melanoma in Portugal. Value Health. 2013;16(3):A139.

5. Linstone HA, Turoff M. The Delphi Method: Techniques and Applications E-Book. Newark: Institute of Technology.

6. Keeney S, Hasson F, McKenna H. The Delphi technique, in the Delphi Technique in Nursing and Health Research. Wiley-Blackwell; p. 1–17.

### ORAL PRESENTATIONS

#### Molecular and immuno-advances

##### O1 Ipilimumab treatment results in CD4 T cell activation that is concomitant with a reduction in Tregs and MDSCs

###### Yago Pico de Coaña^1^, Maria Wolodarski^1^, Yuya Yoshimoto^1^, Giusy Gentilcore^1,2^, Isabel Poschke^1,3^, Giuseppe V. Masucci^1^, Johan Hansson^1^, Rolf Kiessling^1^

####### ^1^Department of Oncology-Pathology, Cancer Center Karolinska, Karolinska Institutet, Stockholm, Sweden; ^2^Division of Translational Medicine, Sidra Medical and Research Center, Doha, Qatar; ^3^Division of Molecular Oncology of Gastrointestinal Tumors, German Cancer Research Center, Heidelberg, Germany

######## **Correspondence:** Yago Pico de Coaña

*Journal of Translational Medicine* 2016, **14(Suppl 1)**:O1

**Background:** Blocking the immune checkpoint molecule Cytotoxic T-lymphocyte antigen-4 (CTLA-4) with ipilimumab has proven to induce long-lasting clinical responses in patients with metastatic melanoma. Although the theoretic base for the success of this therapy has been described, the detailed mechanisms involved are not yet fully understood. In addition to this, there is a great need of predictive, prognostic and pharmacodynamic biomarkers that may allow clinicians to screen for immune related adverse events and potentially provide information for patient preselection. In this study we show the results derived from the immune monitoring of advanced melanoma patients undergoing treatment with ipilimumab.

**Materials and methods:** Peripheral blood mononuclear cells (PBMCs) were isolated from 42 melanoma patients at three time points before and during treatment. PBMCs were stained with 12 multi-color flow cytometric panels within 2 h of sample collection.

**Results:** The results show that ipilimumab treatment contributes to activate CD 4 T cells as is shown by a significant increase in the frequency of ICOS + CD4 cells. This increase is correlated with a decrease in the frequency of granulocytic MDSCs (grMDSCs) and their suppressive potential, determined by their intracellular Arg1 levels. In contrast to these results, monocytic MDSC (moMDSC) frequency was not affected by CTLA-4 blockade, but the frequency of iNOS + cells within this MDSC subset was diminished. This suggests that that at least one of the suppressive mechanisms used by moMDSCs is impaired. Regulatory T cells (Tregs) were also reduced, although their pattern did not correlate with MDSC or activated T cell frequency.

In the 42 patients included, median OS was 55 weeks from the start of treatment. Adverse events were observed in 16 patients (38 %), including eight grade III–IV (19 %). Patients were classified according to their response as PD (progressive disease, 57 %), SD (stable disease, 23 %) and PR (partial response, 20 %). Possible correlations between treatment outcome and MDSC frequencies are currently being evaluated as the patient cohort increases.

**Conclusions:** These results suggest that there may be more than one mechanism of action by which ipilimumab releases the brake on the immune system. On one hand, blocking CTLA-4 results in an in cis effect on the activation status of CD4+ T cells. On the other hand the observed reduction in Tregs and MDSCs (in frequency and suppressive potential) may contribute to significantly alleviate the suppression exerted on the immune system. These effects and their possible clinical implications should be further explored in order to fully understand the mechanisms of action of CTLA-4 blockade with ipilimumab.

##### O2 Evaluation of prognostic and therapeutic potential of COX-2 and PD-L1 in primary and metastatic melanoma

###### Giosuè Scognamiglio^1^, Francesco Sabbatino^2^, Federica Zito Marino^1^, Anna Maria Anniciello^1^, Monica Cantile^1^, Margherita Cerrone^1^, Stefania Scala^3^, Crescenzo D’alterio^3^, Angela Ianaro^4^, Giuseppe Cirino^4^, Paolo Antonio Ascierto^5^, Giuseppina Liguori^1^, Gerardo Botti^1^

####### ^1^Pathology Unit, Istituto Nazionale per lo Studio e la Cura dei Tumori Fondazione “G. Pascale”, Napoli, Italy; ^2^Faculty of Pharmacy and Medicine, Department of Medicine, University of Salerno, Baronissi, Salerno, Italy; ^3^Molecular Immunology and Immuneregulation, Istituto Nazionale per lo Studio e la Cura dei Tumori “Fondazione G. Pascale”, Napoli, Italy; ^4^Department of Pharmacy, University of Naples Federico II, Napoli, Italy; ^5^Unit of Medical Oncology and Innovative Therapy, Istituto Nazionale per lo Studio e la Cura Tumori Fondazione “G. Pascale”, Napoli, Italy

######## **Correspondence:** Giosuè Scognamiglio

*Journal of Translational Medicine* 2016, **14(Suppl 1)**:O2

**Background:** Melanoma is a malignant tumor highly immuno-modulated, therefore different immunotherapy approaches in recent years have been developed. In addition, the American Society of Clinical Oncology (ASCO) Annual Meeting 2015 has emphasized the use of combined treatment strategies involving immunotherapies and/or biological agents target [1].

PD-L1 (programmed cell death ligand 1) is a membrane receptor that plays a key role in the suppression of immune responses, mainly by binding to PD-1 on T lymphocytes. PD-L1 can be expressed in metastatic melanoma and may be an independent prognostic marker in melanoma [2, 3]. Currently, target therapies against both PD-L1 and PD-1 are adopt in clinical trial. COX-2 is an enzyme that catalyzes the conversion of arachidonic acid to prostaglandins and active thromboxanes. Their activity occurs in the sites in which is present an inflammatory state. FANS can cause blocking of their biosynthesis. Several studies in the literature have shown aberrant expression of COX-2 in melanoma, especially in advanced stages [4].

The purpose of this study was to analyze the expression of COX-2 and PD-L1 in a series of primary melanomas and lymph node metastases in order to establish their prognostic value and provide information on the possibility of adopting combined therapeutic strategies.

**Materials and methods:** We have prepared a Tissue Micro Array containing 90 samples, consisting of 45 primary and 45 lymph node metastases from melanoma. On the sections of TMA the expression of COX-2 and PD-L1 (clone 5H1) was evaluated by immunohistochemical staining.

**Results:** Immunohistochemical analysis performed on samples of primary melanomas revealed that 20/45 (44.4) cases were positive for PDL1 and 16/45 (35.5 %) for COX2; for lymph node metastasis 23/45 (51.1 %) cases were positive for PDL1 and 19/45 (42.2 %) cases for COX2.

Our results demonstrated that the expression of COX-2 and PD-L1 were directly related both with primary tumors (p value = 0.003) and with the lymph node metastasis (p value = 0.002) of melanoma. In particular COX2 and PDL1 co-expression in primary melanomas was present in 12/45 cases (26.6 %) while it was absent in 21/45 (46.7 %) cases.

Instead the expression of COX2 and PDL1 in the lymph node metastases were present in 18/45 cases (40 %) while it is absent in 15/45 (33.3 %) cases.

**Conclusions:** The combined expression of both markers either in primary tumors than in lymph node metastases from melanoma could suggest new therapeutic strategies in the modulation of PD-L1 and COX-2 inhibitors.

References

1. Ascierto PA, Marincola FM, Atkins MB.What’s new in melanoma? Combination! J Transl Med. 2015;13:213.

2. Gadiot J, Hooijkaas AI, Kaiser AD, van Tinteren H, van Boven H, Blank C. Overall survival and PD-L1 expression in metastasized malignant melanoma. Cancer. 2011;117(10):2192–201.

3. Massi D, Brusa D, Merelli B, Ciano M, Audrito V, Serra S, Buonincontri R, Baroni G, Nassini R, Minocci D, Cattaneo L, Tamborini E, Carobbio A, Rulli E, Deaglio S, Mandalà M. PD-L1 marks a subset of melanomas with a shorter overall survival and distinct genetic and morphological characteristics. Ann Oncol. 2014;25(12):2433–42.

4. Becker MR, Siegelin MD, Rompel R, Enk AH, Gaiser T. COX-2 expression in malignant melanoma: a novel prognostic marker? Melanoma Res. 2009;19(1):8–16.

##### O3 Vemurafenib in patients with *BRAF*^V600^ mutation–positive metastatic melanoma: final overall survival results of the BRIM-3 study

###### Paul B. Chapman^1^, Caroline Robert^2^, James Larkin^3^, John B. Haanen^4^, Antoni Ribas^5^, David Hogg^6^, Omid Hamid^7^, Paolo Antonio Ascierto^8^, Alessandro Testori^9^, Paul Lorigan^10^, Reinhard Dummer^11^, Jeffrey A. Sosman^12^, Keith T. Flaherty^13^, Huibin Yue^14^, Shelley Coleman^15^, Ivor Caro^16^, Axel Hauschild^17^, Grant A. McArthur^18^

####### ^1^Melanoma and Immunotherapies Service, Memorial Sloan Kettering Cancer Center, New York, NY, USA; ^2^Dermatology Unit, Institut Gustave Roussy, Paris, France; ^3^Department of Medical Oncology and Hematology, The Royal Marsden Hospital, London, UK; ^4^Division of Immunology, The Netherlands Cancer Institute, Amsterdam, The Netherlands; ^5^Department of Medicine, Jonsson Comprehensive Cancer Center at University of California, Los Angeles, Los Angeles, CA, USA; ^6^Department of Medicine, Princess Margaret Hospital and University Health Network, Toronto, Canada; ^7^Melanoma Center, The Angeles Clinic and Research Institute, Los Angeles, CA, USA; ^8^Unit of Melanoma, Cancer Immunotherapy and Innovative Therapy, Istituto Nazionale Tumori Fondazione Pascale, Naples, Italy; ^9^Divisione chirurgia dermatoncologica, Europeo di Oncologia, Milan, Italy; ^10^Institute of Cancer Sciences, University of Manchester, Manchester, UK; ^11^Dermatologische Klinik, University of Zurich, Zurich, Switzerland; ^12^Division of Hematology and Oncology, Vanderbilt University School of Medicine, Nashville, TN, USA; ^13^Department of Medicine, Massachusetts General Hospital, Boston, MA, USA; ^14^Department of Biostatistics, Genentech Inc., San Francisco, CA, USA; ^15^Product Development Oncology, Genentech Inc., San Francisco, CA, USA; ^16^Dermatology Product Development, Genentech Inc., San Francisco, CA, USA; ^17^Department of Dermatology, University of Kiel, Kiel, Germany; ^18^Skin and Melanoma Service, Peter MacCallum Cancer Centre, East Melbourne, Australia

######## **Correspondence:** Paul B. Chapman

*Journal of Translational Medicine* 2016, **14(Suppl 1)**:O3

**Background:** The BRIM-3 trial showed improved progression-free survival (PFS) and overall survival (OS) for vemurafenib compared with dacarbazine in drug-naive *BRAF*^V600^ mutation–positive metastatic melanoma patients. Vemurafenib has shown a 74 % reduction in the risk for death or disease progression and a 63 % reduction in the risk for death compared with dacarbazine (*P* < 0.001 for both comparisons).

**Materials and methods:** Patients were randomly assigned to receive vemurafenib (960 mg BID) or dacarbazine (1000 mg/m^2^ every 3 week). OS and PFS were coprimary end points. OS was assessed in the intention-to-treat population, with and without censoring of data for dacarbazine patients who crossed over to vemurafenib. Effects of age, disease stage, baseline lactate dehydrogenase value, baseline Eastern Cooperative Oncology Group performance status (ECOG PS) value, and prior therapies were also analyzed by treatment received.

**Results:** A total of 675 patients were randomly assigned to receive vemurafenib (n = 337) or dacarbazine (n = 338). At the time of database lock (Aug 14, 2015), median follow-up was 13.37 months (range 0.4–59.6) for vemurafenib and 9.15 months (range 0–56.2) for dacarbazine. Of 338 patients initially randomly assigned to receive dacarbazine, 84 crossed over to vemurafenib. Subgroup analyses and final OS data will be presented.

**Conclusions:** Extended follow-up of patients enrolled in BRIM-3 provides insight regarding long-term efficacy and safety of vemurafenib in patients with metastatic melanoma and *BRAF*^V600^ mutations.

**Clinical trial registration number:** NCT01006980.

**Acknowledgement:** Supported by F. Hoffman-La Roche Ltd.

##### O4 Updated survival, response and safety data in a phase 1 dose-finding study (CA209-004) of concurrent nivolumab (NIVO) and ipilimumab (IPI) in advanced melanoma

###### Mario Sznol^1^, Margaret K. Callahan^2^, Harriet Kluger^1^, Michael A. Postow^2,3^, RuthAnn Gordan^2^, Neil H. Segal^2^, Naiyer A. Rizvi^2^, Alexander Lesokhin^2^, Michael B. Atkins^4^, John M. Kirkwood^5^, Matthew M. Burke^1^, Amanda Ralabate^1^, Angel Rivera^1^, Stephanie A. Kronenberg^2^, Blessing Agunwamba^2^, Mary Ruisi^6^, Christine Horak^7^, Joel Jiang^6^, Jedd Wolchok^2^

####### ^1^Yale University School of Medicine and Smilow Cancer Center, Yale-New Haven Hospital, New Haven, CT, USA; ^2^Memorial Sloan Kettering Cancer Center, New York, NY, USA; ^3^Weill Cornell Medical College, New York, NY, USA; ^4^Georgetown-Lombardi Comprehensive Cancer Center, Washington, DC, USA; ^5^University of Pittsburgh Medical Center, Pittsburgh, PA, USA; ^6^Bristol-Myers Squibb, Princeton, NJ, USA; ^7^Bristol-Myers Squibb, Lawrenceville, NJ, USA

######## **Correspondence:** Michael A. Postow

*Journal of Translational Medicine* 2016, **14(Suppl 1)**:O4

**Background:** The phase 3 study CheckMate 067 (NCT01844505) showed improved progression-free survival (PFS) and objective response rate (ORR) by RECIST v1.1 for NIVO (anti-PD-1) + IPI (anti-CTLA-4) (median 11.5 months [mo], 58 %) or NIVO (6.9 months, 44 %) compared with IPI (2.9 months, 19 %) in patients (pts) with previously untreated advanced melanoma [1]; however, long-term follow-up data including overall survival (OS) are not yet available. Updated data from CA209-004 (NCT01024231) in pts with unresectable or metastatic melanoma are reported here.

**Materials and methods:** Cohorts [C] 1, 2, 2a, and 3 received NIVO + IPI Q3W × 4 followed by NIVO Q3W × 4; pts without progressive disease or dose-limiting toxicity received NIVO + IPI Q12W × 8. An expansion cohort (C8) received NIVO 1 mg/kg + IPI 3 mg/kg Q3W × 4 followed by NIVO 3 mg/kg Q2W (regimen used in phase 2/3 trials). Data are reported for C1-3 (n = 53) and C8 (n = 41).

**Results:** 40 % of pts in C1-3 and 51 % of pts in C8 were previously treated. At a median follow-up of 32.7 months (range: 2.5–61.4) for C1-3 and 19.9 months (range: 0.9–24.0) for C8, ORR by mWHO criteria was 42 and 44 %, respectively, and the median duration of response was 22.3 and 13.8 months. Complete response was seen in 21 % of pts in C1-3 and 17 % of pts in C8, and the median tumor burden reduction was 70 and 58 %, respectively. OS rates were 79 % at 24 months in C1-3 and 68 % at 18 months in C8. PFS rates were 30 % at 24 months in C1-3 and 27 % at 18 months in C8. Incidence of drug-related adverse events (AEs) was 56 % for grade 3/4 AEs and 39 % for grade 3/4 select AEs in C1-3 and C8 combined (n = 94).

**Conclusions:** OS for concurrent NIVO + IPI was higher than expected for each agent alone in this population, with a manageable safety profile.

Reused with permission from the Society for Melanoma Research (SMR) and all authors.

Reference

1. Larkin J, Chiarion-Sileni V, Gonzalez R, Grob JJ, Cowey CL, Lao CD, et al. Combined nivolumab and ipilimumab or monotherapy in untreated melanoma. N Engl J Med. 2015;373:23–34.

#### Combination therapies

##### O5 Efficacy and correlative biomarker analysis of the coBRIM study comparing cobimetinib (COBI) + vemurafenib (VEM) vs placebo (PBO) + VEM in advanced *BRAF*-mutated melanoma patients (pts)

###### Paolo A. Ascierto^1^, Grant A. McArthur^2^, James Larkin^3^, Gabriella Liszkay^4^, Michele Maio^5^, Mario Mandalà^6^, Lev Demidov^7^, Daniil Stoyakovskiy^8^, Luc Thomas^9^, Luis de la Cruz-Merino^10^, Victoria Atkinson^11^, Caroline Dutriaux^12^, Claus Garbe^13^, Matthew Wongchenko^14^, Ilsung Chang^15^, Daniel O. Koralek^15^, Isabelle Rooney^15^, Yibing Yan^14^, Antoni Ribas^16^, Brigitte Dréno^17^

####### ^1^Unit of Melanoma, Cancer Immunotherapy and Innovative Therapy, Istituto Nazionale Tumori Fondazione G. Pascale, Naples, Italy; ^2^Skin and Melanoma Service, Peter MacCallum Cancer Centre, Melbourne, VIC, Australia; ^3^Department of Medical Oncology and Hematology, The Royal Marsden Hospital, London, UK; ^4^Division of Cancer Services National Institute of Oncology, Budapest, Hungary; ^5^Division of Medical Oncology and Immunotherapy, Azienda Ospedaliera Universitaria Senese, Siena, Italy; ^6^Division of Medical Oncology, Papa Giovanni XXIII Hospital, Bergamo, Italy; ^7^Department of Biotherapy, N. N. Blokhin Russian Cancer Research Center, Moscow, Russia; ^8^Department of Chemotherapy, Moscow City Oncology Hospital 62, Krasnogorsk, Russia; ^9^Department of Dermatology, Centre Hospitalier Lyon Sud, Pierre-Bénite, France; ^10^Department of Clinical Oncology, Hospital Universitario Virgen Macarena, Seville, Spain; ^11^Division of Cancer Services, Princess Alexandra Hospital, Woolloongabba, QLD, Australia; ^12^Department of Dermato-Oncology, Hôpital Saint André, Bordeaux, France; ^13^Deptartment of Dermatology, University of Tübingen, Tübingen, Germany; ^14^Oncology Biomarker Development, Genentech, Inc., South San Francisco, CA, USA; ^15^Product Development Oncology, Genentech, Inc., South San Francisco, CA, USA; ^16^Department of Medicine, Jonsson Comprehensive Cancer Center at the University of California, Los Angeles, Los Angeles, CA, USA; ^17^Department of Dermato Cancerology, Nantes University, Nantes, France

######## **Correspondence:** Paolo A. Ascierto

*Journal of Translational Medicine* 2016, **14(Suppl 1)**:O5

**Background:** The primary analysis of coBRIM showed superior progression-free survival (PFS) for COBI + VEM in advanced *BRAF*-mutated melanoma pts [1]. We present the protocol-defined, updated PFS analysis and final OS analysis and correlate these efficacy results to key biomarkers.

**Materials and methods:** coBRIM has been described previously [1]. Pts were randomly assigned to receive COBI + VEM or PBO + VEM. Primary endpoint was investigator-assessed PFS; overall survival (OS) was a secondary endpoint. Following the primary PFS analysis, coBRIM remained blinded, and pts were followed up for OS. Final OS analysis occurred after 250 events. For biomarker analysis, pretreatment tumor samples were evaluated for *BRAF*^*V600*^ allele frequency (*BRAF*^V600^ copy number) by targeted deep sequencing and pathway activation by pERK, pS6, and PTEN, and Ki67 by immunohistochemistry. Cox proportional hazards models were used to assess the association between OS and biomarkers.

**Results:** From Jan 2014 to Jan 2015, 495 pts were randomly assigned. Baseline characteristics in COBI + VEM and PBO + VEM pts were Eastern Cooperative Oncology Group (ECOG) 0: 76 and 67 %; stage IV M1c: 59 and 62 %; LDH high: 46 and 43 %. Updated PFS analysis was conducted after a median follow-up of 14.3 months and showed a median PFS of 12.3 months for COBI + VEM vs 7.2 months for PBO + VEM (HR, 0.58; 95 % CI; 0.46–0.72). PFS treatment benefit was seen across all prespecified patient subgroups, including patients with LDH high (COBI + VEM 8.2 months vs PBO + VEM 5.4 months; HR, 0.57; 95 % CI, 0.42–0.78) and normal (COBI + VEM 13.4 months vs PBO + VEM 7.8 months; HR, 0.59; 95 % CI, 0.43–0.81).

Final OS analysis was conducted after a median follow-up of 18.5 months and confirmed a statistically significant and clinically meaningful benefit for pts receiving COBI + VEM. OS data and correlation of key biomarkers with OS and PFS will be presented.

**Conclusions:** Updated PFS and OS data confirm the clinical benefit of COBI + VEM across all clinical and molecular subgroups vs PBO + VEM in patients with advanced BRAF-mutated melanoma.

Reference

1. Larkin J, Ascierto PA, Dreno B, et al. Combined vemurafenib and cobimetinib in BRAF-mutated melanoma. N Engl J Med. 2014;371:1867–76.

##### O6 Preliminary clinical safety, tolerability and activity results from a Phase Ib study of atezolizumab (anti-PDL1) combined with vemurafenib in *BRAF*^V600^-mutant metastatic melanoma

###### Ryan Sullivan^1^, Omid Hamid^2^, Manish Patel^3^, Stephen Hodi^1^, Rodabe Amaria^4^, Peter Boasberg^2^, Jeffrey Wallin^5^, Xian He^5^, Edward Cha^5^, Nicole Richie^5^, Marcus Ballinger^5^, Patrick Hwu^4^

####### ^1^Dana-Farber Cancer Institute, Boston, MD, USA; ^2^The Angeles Clinic and Research Institute, Los Angeles, CA, USA; ^3^Florida Cancer Specialists/Sarah Cannon Research Institute, Sarasota, FL, USA; ^4^The University of Texas MD Anderson Cancer Center, Houston, TX, USA; ^5^Genentech, Inc., South San Francisco, CA, USA

######## **Correspondence:** Ryan Sullivan

*Journal of Translational Medicine* 2016, **14(Suppl 1)**:O6

**Background:** BRAF-targeted therapy with vemurafenib (V) is associated with increased immune activation in *BRAF*^V600^-mutant melanoma, resulting in a rapid clinical response. The durability of response can potentially be further extended by combining V and atezolizumab (A; MPDL3280A), which may enhance and perpetuate antitumor immune activity.

**Materials and methods:** Patients with untreated *BRAF*^V600^-mutant unresectable or metastatic melanoma received A + V combination concurrently (cohort 1 [C1], n = 3) or after a run-in period with V alone (56 days for cohort 2 [C2, n = 8]; 28 days for cohort 3 [C3, n = 6]). Administration of A was IV q3w at 20 mg/kg (C1) and 15 mg/kg or 1200 mg fixed (C2 and C3). V was administered orally BID 960 mg during run-in and 720 mg during the A + V combination period. PD-L1 expression on tumor cells and tumor-infiltrating immune cells was centrally examined by immunohistochemistry (SP142 assay).

**Results:** Of 19 BRAF inhibitor—and immunotherapy-naive patients enrolled, 17 who received ≥1 dose of A were safety and efficacy evaluable (data cutoff, Mar 16, 2015). Across cohorts, 65 % of patients were male and median age was 53 y (range, 30–71 y). Patients received A + V combination for a median of 8.5 months (range, 1.2–27.8 months). Median safety follow-up (mo) was 6.5 (C1), 10.6 (C2) and 10.4 (C3). A + V did not result in any DLTs or unexpected AEs. Incidence of A- or V-related G3 AEs was 41 and 59 %, respectively, and was higher in C1 (67 % A; 100 % V) than in C2 (38 % A; 50 % V) or C3 (33 % A; 50 % V). AEs were manageable and generally reversible. Related G4 AEs were absent. No G5 AEs occurred. There were no A-related AEs leading to treatment discontinuation; 1 patient in C1 had V-related AEs leading to discontinuation. Clinical activity results are presented in Table 1. Higher ORR was seen with V run-in (C2, 75 %; C3, 100 %) than with concurrent A + V start (C1, 33 %). Preliminary data indicate an increase in PD-L1 expression in repeat biopsies after V run-in or A + V combination, suggesting induction of an enhanced antitumor immune response.

**Table 1:** Clinical activity results

	Cohort 1 (n = 3)	Cohort 2 (n = 8)	Cohort 3 (n = 6)	All (n = 17)
Objective response (confirmed, RECIST v1.1)				
ORR, % (n)	33 % (1)	75 % (6)	100 % (6)	76 % (13)
CR, % (n)	33 % (1)	12 % (1)	17 % (1)	18 % (3)
PR, % (n)	0	62 % (5)	83 % (5)	59 % (10)
PFS				
Median, mo (95 % CI)	2.7 (1.7, 22.0)	12.2 (4.6, NE)	NE (NE)	12.2 (5.7–22.0)
DOR				
Median, mo (95 % CI)	20.9 (NE)	10.6 (4.9, NE)	NE (NE)	20.9 (10.6, 20.9)

**Conclusions:** Overall, a manageable safety profile and promising antitumor activity were demonstrated with A + V combination therapy. In particular, staggered dosing of A with V run-in was better tolerated and yielded high rates of durable response. Triple-combination therapy with A + V+cobimetinib (MEK inhibitor) in this patient population is under investigation. (NCT01656642).

References

1. Larkin J, Chiarion-Sileni V, Gonzalez R, Grob JJ, Cowey CL, Lao CD, et al. Combined nivolumab and ipilimumab or monotherapy in untreated melanoma. N Engl J Med. 2015;373:23–34

##### O7 Preliminary safety and efficacy data from a phase 1/2 study of epacadostat (INCB024360) in combination with pembrolizumab in patients with advanced/metastatic melanoma

###### Thomas F. Gajewski^1^, Omid Hamid^2^, David C. Smith^3^, Todd M. Bauer^4^, Jeffrey S. Wasser^5^, Jason J. Luke^6^, Ani S. Balmanoukian^1^, David R. Kaufman^7^, Yufan Zhao^8^, Janet Maleski^8^, Lance Leopold^8^, Tara C. Gangadhar^9^

####### ^1^Department of Pathology and Department of Medicine-Section of Hematology/Oncology, University of Chicago, Chicago, IL, USA; ^2^Research Department, The Angeles Clinic and Research Institute, Los Angeles, CA, USA; ^3^Department of Internal Medicine and Department of Urology, University of Michigan, Ann Arbor, MI, USA; ^4^Drug Development Unit, Sarah Cannon Research Institute/Tennessee Oncology PLLC., Nashville, TN, USA; ^5^Division of Hematology and Medical Oncology, University of Connecticut Health Center, Farmington, CT, USA; ^6^Department of Medicine-Section of Hematology/Oncology, University of Chicago, Chicago, USA; ^7^ Merck & Co. Inc., Merck Research Laboratories, Kenilworth, NJ, USA; ^8^ Incyte Corporation, Drug Development, Wilmington, DE, USA; ^9^Hematology-Oncology Division, Abramson Cancer Center of the University of Pennsylvania, Philadelphia, PA, USA

######## **Correspondence:** Thomas F. Gajewski

*Journal of Translational Medicine* 2016, **14(Suppl 1)**:O7

**Background:** Indoleamine 2,3-dioxygenase 1 (IDO1) is a tryptophan-catabolizing enzyme that induces immune tolerance by suppressing T-cell responses. Epacadostat is a potent, selective oral IDO1 inhibitor. In a dose-escalation study in patients with metastatic melanoma, the combination of epacadostat with ipilimumab was generally tolerated and favorable response rates were observed in immunotherapy-naive patients.

**Materials and methods:** NCT02178722 is an ongoing study of epacadostat with pembrolizumab in patients with advanced melanoma and other cancers with a 3 + 3+ 3 phase 1 design. Patients previously treated with checkpoint inhibitors were excluded. During dose-escalation, patients received epacadostat 25, 50, or 100 mg BID with pembrolizumab 2 mg/kg IV every 3 weeks or epacadostat 300 mg BID with pembrolizumab 200 mg IV every 3 weeks. During dose-expansion, patients received epacadostat 50, 100, or 300 mg BID with pembrolizumab 200 mg IV every 3 weeks. Safety, tolerability, and investigator-assessed tumor response (RECIST 1.1) were evaluated.

**Results:** As of 29 Oct 2015, 62 patients were enrolled. Safety data on 56 patients with either melanoma (n = 20), RCC (n = 11), NSCLC (n = 10), transitional cell carcinoma (n = 5), endometrial adenocarcinoma (n = 5), TNBC (n = 3), or SCCHN (n = 2) as of October 1, 2015 are presented. The most common all grade treatment-related AEs were rash (25 %), fatigue (23 %), arthralgia (13 %), pruritus (13 %), nausea (11 %), and pyrexia (11 %). Grade 3 treatment-related AEs occurred in 11 % of patients; the most common grade 3 treatment-related AE was rash (5 %). There were no grade 4 treatment-related AEs and no treatment-related deaths. One patient discontinued for a treatment-related AE (grade 3 AST elevation). In 19 efficacy evaluable melanoma patients, ORR was 53 % and disease control rate was 74 % (3 CRs, 7 PRs, 4 SDs). Responses occurred in patients who were PD-L1-positive and PD-L1-negative (PD-L1 IHC 22C3 pharmDx Kit; ≥1 % cutoff for positivity). All responses are ongoing as of the data cutoff.

**Conclusion:** Preliminary safety data support continued evaluation of epacadostat with pembrolizumab. Although preliminary, the efficacy data suggest promising clinical activity of the combination in patients with advanced/metastatic melanoma. Updated safety data will be presented.

##### O8 Primary analysis of MASTERKEY-265 phase 1b study of talimogene laherparepvec (T-VEC) and pembrolizumab (pembro) for unresectable stage IIIB-IV melanoma

###### Reinhard Dummer^1^, Georgina V. Long^2^, Antoni Ribas^3^, Igor Puzanov^4^, Olivier Michielin^5^, Ari VanderWalde^6^, Robert H.I. Andtbacka^7^, Jonathan Cebon^8^, Eugenio Fernandez^9^, Josep Malvehy^10^, Anthony J. Olszanski^11^, Thomas F. Gajewski^12^, John M. Kirkwood^13^, Christine Gause^14^, Lisa Chen^15^, David R. Kaufman^14^, Jeffrey Chou^15^, F. Stephen Hodi^16^

####### ^1^University Hospital of Zurich, Zurich, Switzerland; ^2^Melanoma Institute Australia, The University of Sydney and The Mater Hospital, Sydney, Australia; ^3^University of California at Los Angeles Medical Center, Los Angeles, CA, USA; ^4^Vanderbilt University Medical Center, Nashville, TN, ISA; ^5^Centre Hospitalier Universitaire Vaudois; Lausanne, Switzerland; ^6^The West Clinic, Memphis, TN, USA; ^7^University of Utah Huntsman Cancer Institute, Salt Lake City, UT; ^8^Olivia Newton-John Cancer Center, Heidelberg, Australia; ^9^Hopitaux Universitaires de Geneve; Geneva, Switzerland; ^10^Hospital Clinic i Provincial de Barcelona, Barcelona, Spain; ^11^Fox Chase Cancer Center, Philadelphia, PA, USA; ^12^The University of Chicago Medicine, Chicago, IL, USA; ^13^University of Pittsburgh Cancer Institute, Pittsburgh, PA, USA; ^14^Merck & Co., Inc., Kenilworth, NJ, USA; ^15^Amgen Inc., Thousand Oaks, CA, USA; ^16^Dana-Farber Cancer Institute, Boston, MA, USA

######## **Correspondence:** Reinhard Dummer

*Journal of Translational Medicine* 2016, **14(Suppl 1)**:O8

**Background:** T-VEC is a herpes simplex virus-1 -based oncolytic immunotherapy designed to selectively replicate in tumors, produce GM-CSF, and stimulate antitumor immune responses. T-VEC significantly improved durable response rate (DRR; primary endpoint) in the T-VEC arm vs GM-CSF in OPTiM (Andtbacka et al. JCO 2015) and is approved in the US for local treatment of unresectable cutaneous, subcutaneous, and nodal lesions in patients with melanoma recurrent after initial surgery. Pembro is a PD-1 blocking antibody approved for the treatment of advanced metastatic or unresectable melanoma and has demonstrated superiority over the CTLA-4-blocking antibody ipilimumab in patients with stage III or IV melanoma (Robert et al. NEJM 2015). Both drugs have tolerable and non-overlapping adverse event (AE) profiles, and combining them may enhance antitumor efficacy. Safety and preliminary efficacy data from the phase 1b primary analysis of a phase 1b/3 study of T-VEC + pembro in unresectable stage IIIB-IV melanoma (NCT02263508) are reported.

**Materials and methods:** The primary endpoint is incidence of dose-limiting toxicities (DLT). Key secondary endpoints are objective response, survival, and AEs. Patients had stage IIIB-IV melanoma with injectable lesions, no prior systemic therapy, and ECOG PS 0-1. T-VEC was given ≤4 mL injected into (sub)cutaneous/nodal lesions 10^6^ PFU/mL d1, 10^8^ PFU/mL d22 then Q2W. Pembro was given IV 200 mg d36 then Q2W. Treatment (tx) continues until.

CR, all injectable tumors have disappeared (for T-VEC), confirmed PD per modified immune-related response criteria, intolerance of study treatment, 24 months from the date of the first dose of pembro or end of study, whichever occurs first.

**Results:** 21 patients were enrolled Dec 2014-Mar 2015 with data cutoff of Jun 2015. Patient characteristics: median age 58 year; 48 % stage IIIB-IVM1a, 52 % stage IVM1b/c melanoma; 81 % PD-L1+; 76 % HSV+. Median follow-up time was 18.7 w. No DLTs were reported. All 21 patients had an AE: 29 % G3, no G4, and one G5 (not attributed to tx). Most common AEs were fatigue 52 %, pyrexia 48 %, chills 43 %, and rash 38 %. Of 16 patients ≥12 w after first pembro tx and with evaluable response assessment, unconfirmed response rate per investigator was 56 %; disease control rate was 69 % (12.5 % CR, 44 % PR, 12.5 % SD, 31 % PD).

**Conclusions**: T-VEC+ pembro can be given at full doses with no unexpected safety signals. Responses were seen in over half of evaluable patients in this early efficacy analysis. A randomized phase 3 trial comparing T-VEC+ pembro vs T-VEC placebo + pembro is planned.

#### News in immunotherapy

##### O9 Two-year survival and safety update in patients (pts) with treatment-naïve advanced melanoma (MEL) receiving nivolumab (NIVO) or dacarbazine (DTIC) in CheckMate 066

###### Victoria Atkinson^1^, Paolo A. Ascierto^2^, Georgina V. Long^3^, Benjamin Brady^4^, Caroline Dutriaux^5^, Michele Maio^6^, Laurent Mortier^7^, Jessica C. Hassel^8^, Piotr Rutkowski^9^, Catriona McNeil^10^, Ewa Kalinka-Warzocha^11^, Celeste Lebbé^12^, Lars Ny^13^, Matias Chacon^14^, Paola Queirolo^15^, Carmen Loquai^16^, Parneet Cheema^17^, Alfonso Berrocal^18^, Karmele Mujika Eizmendi^19^, Luis De La Cruz-Merino^20^, Gil Bar-Sela^21^, Christine Horak^22^, Joel Jiang^22^, Helene Hardy^22^, Caroline Robert^23^

####### ^1^Princess Alexandra Hospital, Woolloongabba, and Gallipoli Medical Research Foundation, Greenslopes, QLD, Australia; ^2^Istituto Nazionale Tumori Fondazione Pascale, Naples, Italy; ^3^Melanoma Institute Australia, Sydney, NSW, Australia; ^4^Cabrini Health, Melbourne, VIC, Australia; ^5^Hôpital Saint André Centre Hospitalier Universitaire, Bordeaux, France; ^6^University Hospital of Siena, Siena, Italy; ^7^Hôpital Claude Huriez, Lille, France; ^8^University Hospital Heidelberg, Heidelberg, Germany; ^9^Sklodowska-Curie Memorial Cancer Center, Warsaw, Poland; ^10^Chris O’Brien Lifehouse, Royal Prince Alfred Hospital, and Melanoma Institute Australia, Camperdown, NSW, Australia; ^11^Wojewódzki Szpital Specjalistyczny im. M., Lodz, Poland; ^12^Hôpital Saint-Louis University Paris Diderot, Paris, France; ^13^University of Gothenburg, Gothenburg, Sweden; ^14^Instituto Alexander Fleming, Buenos Aires, Argentina; ^15^IRCCS San Martino, IST Istituto Nazionale per la Ricerca sul Cancro, Genova, Italy; ^16^University Hospital Mainz, 55122, Mainz, Germany; ^17^Sunnybrook Health Sciences Centre, Toronto, Canada; ^18^Consorcio Hospital General Universitario de Valencia, Valencia, Spain; ^19^Onkologikoa, Donosti Gipuzkoa, Spain; ^20^Hospital Universitario Virgen Macarena, Sevilla, Spain; ^21^Rambam Health Care Campus, Haifa, Israel; ^22^Bristol-Myers Squibb, Hopewell, NJ, USA; ^23^Gustave Roussy and Paris-Sud University, Villejuif Paris-Sud, France

######## **Correspondence:** Paolo A. Ascierto

*Journal of Translational Medicine* 2016, **14(Suppl 1)**:O9

**Background:** In a phase 3, randomized study (CheckMate 066, NCT01721772), NIVO demonstrated increased overall survival (OS) vs DTIC in treatment-naïve BRAF wild-type pts with MEL, and a safety profile consistent with prior reports [1]. Here, we report two-year OS as well as updated efficacy and safety data.

**Materials and methods:** 418 treatment-naïve BRAF wild-type pts with MEL were randomly assigned to receive NIVO (3 mg/kg Q2W and DTIC-matched placebo Q3W) or DTIC (1000 mg/m^2^ Q3W and NIVO-matched placebo Q2W).

**Results:** At a minimum survival follow-up of 15.1 months, NIVO continued to demonstrate significantly improved OS with median OS (mOS) not reached (NR) (95 % CI: 23.1, NR) vs 11.2 months (95 % CI: 9.6, 13.0) with DTIC (hazard ratio [HR]: 0.43 [95 % CI: 0.33, 0.57; P < 0.0001]). OS rates were 70.7 and 57.7 % for NIVO and 46.3 and 26.7 % for DTIC at 12 and 24 months, respectively. Subsequent treatment was used in 48.1 and 72.1 % of pts on NIVO and DTIC, respectively, and 27 DTIC pts crossed over to receive NIVO. Median progression-free survival continued to be significantly greater with NIVO vs DTIC (5.4 vs 2.2 months [HR: 0.42 (95 % CI: 0.32, 0.53; P < 0.0001)]). Objective response rate (ORR) remained higher for NIVO (42.9 %) vs DTIC (14.4 %; P < 0.0001). A higher rate of complete responses was reported with NIVO (10.5 %) vs DTIC (1.0 %); median duration of response was NR vs 7.2 months, respectively. Of 90 responders with NIVO, 64 (71 %) had ongoing responses vs 4/30 (13 %) with DTIC. Drug-related adverse events (AE) were reported in 77 and 78 % of pts on NIVO vs DTIC (drug-related grade 3–4 AEs in 13 and 17 %), respectively. Drug-related AEs (any grade) led to discontinuation in 6 % of NIVO and 3 % of DTIC pts. Among 57 pts who started ipilimumab (IPI) after NIVO, mOS was 9.0 months (95 % CI: 7.1, 14.7) and ORR was 8.8 % (n = 5, all partial responses) after start of IPI. Drug-related AEs were reported in 77 % of pts who switched to IPI.

**Conclusions:** In summary, NIVO provided durable responses and mOS NR with continued acceptable safety after 2 years in pts with MEL.

Reused with permission from the Society for Melanoma Research (SMR) and all authors.

Reference

1. Robert C, Long GV, Brady B, Dutriaux C, Maio M, Mortier L, et al. Nivolumab in previously untreated melanoma without BRAF mutation. N Engl J Med. 2015;372:320-330.

##### O10 Efficacy and safety of nivolumab (NIVO) in patients (pts) with advanced melanoma (MEL) who were treated beyond progression in CheckMate 066/067

###### Georgina V. Long^1^, Jeffrey S. Weber^2^, James Larkin^3^, Victoria Atkinson^4^, Jean-Jacques Grob^5^, Reinhard Dummer^6^, Caroline Robert^7^, Ivan Marquez-Rodas^8^, Catriona McNeil^9^, Henrik Schmidt^10^, Karen Briscoe^11^, Jean-François Baurain^12^, F. Stephen Hodi^13^, Jedd D. Wolchok^14^

####### ^1^Melanoma Institute Australia, The University of Sydney, and Mater Hospital, Sydney, NSW, Australia; ^2^Moffitt Cancer Center, Tampa, FL, USA; ^3^Royal Marsden Hospital, London, UK; ^4^Gallipoli Medical Research Foundation and Princess Alexandra Hospital, QLD, Australia; ^5^Hospital Timone APHM, Aix-Marseille University, Marseille, France; ^6^Universitaets Spital, Zurich, Switzerland; ^7^Gustave Roussy and Paris-Sud University, Villejuif Paris-Sud, France; ^8^Servicio de Oncología Médica, Hospital General Universitario Gregorio Marañón, Madrid, Spain; ^9^Chris O’Brien Lifehouse, The Melanoma Institute Australia & Royal Prince Alfred Hospital, Camperdown, Australia; ^10^Department of Oncology, Aarhus University Hospital, Aarhus, Denmark; ^11^Department of Medical Oncology, Coffs Harbour Health Campus, NSW, Australia; ^12^Cliniques Universitaires Saint-Luc, Bruxelles, Belgium; ^13^Dana-Farber Cancer Institute, Boston, MA, USA; ^14^Ludwig Center at Memorial Sloan Kettering Cancer Center, New York, NY, USA

######## **Correspondence:** Reinhard Dummer

*Journal of Translational Medicine* 2016, **14(Suppl 1)**:O10

**Background:** Responses to immune checkpoint inhibitors may be delayed or may develop after RECIST-defined disease progression [1, 2].

**Materials and methods:** We conducted a retrospective analysis to measure efficacy and safety outcomes in pts treated with NIVO (anti-PD-1 therapy) beyond RECIST-defined disease progression. Data were pooled from treatment-naive MEL pts who received NIVO monotherapy in phase 3 studies CheckMate 066 (NCT01721772) and CheckMate 067 (NCT01844505). Progression was assessed by RECIST v1.1 and investigators determined which pts might benefit from treatment beyond progression.

**Results:** Of all randomized pts (N = 526), 298 (57 %) experienced RECIST-defined disease progression with NIVO monotherapy. Of these 298 pts, 158 (53 %) did not continue NIVO treatment (Non-TBP group) and 140 (47 %) were treated with at least 1 dose of NIVO beyond progression (TBP group). 31/140 (22 %) pts in the TBP group showed greater than 30 % reduction in tumor burden after progression in comparison with baseline data (TBP30, a subset of the TBP group). Median time between progression and last dose of study treatment was 6.7 and 36.1 weeks for TBP and TBP30 groups, respectively. Maximum change in median tumor burden between randomization and first progression was an increase of 5.4 % in the TBP group, an increase of 20.2 % in the Non-TBP group and a reduction of 45.2 % in the TBP30 group. Treatment-related grade 3/4 adverse events (AEs) occurred in 7.0 and 11.4 % of pts in Non-TBP and TBP groups, respectively. Treatment-related select grade 3/4 AEs occurred in 3.8 and 4.3 % of pts in Non-TBP and TBP groups, respectively. Additional descriptive data in Non-TBP and TBP groups at baseline, during treatment and at progression will be presented.

**Conclusions:** These results suggest that pts with RECIST-defined disease progression can safely continue NIVO treatment and that a proportion of these pts may experience prolonged clinical benefit.

Reused with permission from the Society for Melanoma Research (SMR) and all authors.

References

1. Wolchok JD, Hoos A, O’Day S, Weber JS, Hamid O, Lebbé C, et al. Guidelines for the evaluation of immune therapy activity in solid tumors: immune-related response criteria. Clin Cancer Res. 2009;15:7412–20.

2. Ribas A, Chmielowski B, Glaspy JA. Do we need a different set of response assessment criteria for tumor immunotherapy? Clin Cancer Res. 2009;15:7116–8.

#### Tumor microenvironment and biomarkers

##### O11 New biomarkers for response/resistance to BRAF inhibitor therapy in metastatic melanoma

###### Rosamaria Pinto^1,*^, Simona De Summa^1,*^, Vito Michele Garrisi^1^, Sabino Strippoli^1^, Amalia Azzariti^1^, Gabriella Guida^2^, Michele Guida^1^, Stefania Tommasi^1^

####### ^1^Istituto Tumori “Giovanni Paolo II, Bari, Italy; ^2^Università di Bari, Bari, Italy

######## **Correspondence:** Stefania Tommasi

^*^Rosamaria Pinto and Simona De Summa have equally contributed to the work.

*Journal of Translational Medicine* 2016, **14(Suppl 1)**:O11

**Background:** About 50 % of metastatic melanomas (MM) harbour mutations in the BRAF gene, mainly at codon 600, resulting in constitutive activation of the MAPK pathway [1]. The selective inhibitors of BRAF V600, vemurafenib and dabrafenib have shown major tumor responses in about 50 % of patients. However, the majority of patients progress after 6–8 months due to several resistance mechanisms which are only partially understood. As both targeting agents appear to benefit only certain subsets of patients, the identification of predictors of response is mandatory. One of the available strategies that facilitates the simultaneous analysis of a large number of factors in biological material is surface enhanced laser desorption ionization time of flight mass spectrometry (SELDI ToF MS). The purpose of the present study is to verify if this high-throughput technique is able to individuate novel candidate peptides useful as biomarkers to predict the response or resistance to BRAF inhibitors. Moreover, we integrated results of our recent study [2] regarding the relationship between microRNA expression and response to Vemurafenib in order to better understand biological mechanism influencing disease progression.

**Materials and methods:** In order to discover novel candidate biomarkers predictive to treatment, serum of 39 MM vemurafenib treated patients at baseline, at disease control and at progression, were analyzed using SELDI-TOF technology. The Mascot Peptide Mass Fingerprint online tool (http://www.matrixscience.com/cgi/search_form.pl?FORMVER52&SEARCH5PMF) was used to identify more significant peaks and miRWalk (http://zmf.umm.uni-heidelberg.de/apps/zmf/mirwalk2/index.html) to verify interaction between microRNA and genes coding for the predicted proteins.

**Results:** At baseline as predictive biomarkers we identified four peaks—m/z 5900, 12544, 49124 and 11724—significantly up-regulated in longer *versus* shorter responders to vemurafenib. After response, three peptides m/z 9292, 7765 and 9176 appeared up-regulated in responder patients. In silico predictions indicated that the metastatic suppressor gene KLF17 and two cellular proteins, RBM10 and TOX3, which are involved in proliferation and apoptosis of cancer cells, could be the actors influencing the outcome of BRAF inhibitor therapy. TOX3, RMB10 and KLF17 resulted target of miR-132-3P, the miRNA whose lower expression is associated to shorter progression free survival.

**Conclusions:** In conclusion, our results highlighted the possibility to better stratify patients that could be addressed to BRAF inhibitor therapy. Moreover, the combined analysis of epigenetic, microRNAs, and proteomic data could help to uncover the biological mechanism of resistance in MM.

References

1. Fecher LA, Cummings SD, Keefe MJ, Alani RM. Toward a molecular classification of melanoma. J Clinical Oncol. 2007;25:1606–20.

2. Pinto R, Strippoli S, De Summa S et al. MicroRNA expression in BRAF-mutated and wild-type metastatic melanoma and its correlation with response duration to BRAF inhibitors. Expert Opin Ther Targets. 2015; 1–9.

##### O12 Chemokine receptor patterns in lymphocytes mirror metastatic spreading in melanoma and response to ipilimumab

###### Nicolas Jacquelot^1,2,3,*^, David Enot^3,4,*^ Caroline Flament^1,3,5,*^, Jonathan M. Pitt^1,3^, Nadège Vimond^3,5,6^, Carolin Blattner^7,8^, Takahiro Yamazaki^1,3^, Maria-Paula Roberti^1,3^, Marie Vetizou^1,3^, Romain Daillere^1,2,3^, Vichnou Poirier-Colame^1,3^, Michaëla Semeraro^1,9^, Anne Caignard^10^, Craig L Slingluff Jr^11^, Federica Sallusto^12^, Sylvie Rusakiewicz^1,3,5^, Benjamin Weide^13,14^, Aurélien Marabelle^1,3^, Holbrook Kohrt^15^, Stéphane Dalle^16,17^, Andréa Cavalcanti^3^, Guido Kroemer^3,4,17–21^, Anna Maria Di Giacomo^22^, Michele Maio^23^, Phillip Wong^24^, Jianda Yuan^24^, Jedd Wolchok^24^, Viktor Umansky^7,8^, Alexander Eggermont^3^, Laurence Zitvogel^1,2,3,5^

####### ^1^INSERM U1015, Gustave Roussy Cancer Institute, Villejuif, France; ^2^University Paris Sud XI, Kremlin Bicêtre, France; ^3^Gustave Roussy Cancer Institute, Villejuif, France; ^4^Metabolomics and Cell Biology Platforms, Gustave Roussy Cancer Institute, Villejuif, France; ^5^CIC Biotherapie IGR Curie, CIC1428, Gustave Roussy Cancer Institute, Villejuif, France; ^6^Laboratory of Immunomonitoring in Oncology (L.I.O), UMS 3655 CNRS/US 23 INSERM, Gustave Roussy Cancer Institute, Villejuif, France; ^7^Skin Cancer Unit, German Cancer Research Center (DKFZ), Heidelberg, Germany; ^8^Department of Dermatology, Venereology and Allergology, University Medical Center Mannheim, Ruprecht-Karl University of Heidelberg, Mannheim, Germany; ^9^Center of Clinical Investigation Hôpital Necker Enfants Malades, Paris, France; ^10^INSERM U1016, CNRS UMR8104 Cochin Institute, Paris, France; ^11^Division of Surgical Oncology, Department of Surgery, University of Virginia, Charlottesville, VA, USA; ^12^Cellular Immunology Laboratory, Institute for Research in Biomedicine, Università della Svizzera Italiana (USI), Via Vincenzo Vela 6, CH-6500 Bellinzona, Switzerland; ^13^Department of Immunology, Eberhard Karls Universität Tübingen, Tübingen, Germany; ^14^Department of Dermatology, University Medical Center Tübingen, Tübingen, Germany; ^15^Division of Oncology, Department of Medicine, Stanford University, Stanford, California; ^16^Centre Hospitalier Lyon-Sud, Hospices Civils de Lyon, Lyon, France; ^17^University Claude Bernard Lyon 1, Lyon, France; ^18^Equipe 11 labellisée par la Ligue contre le Cancer, Centre de Recherche des Cordeliers, Paris, France; ^19^INSERM, U1138, Paris, France; ^20^Université Paris Descartes, Sorbonne Paris Cité, Paris, France; ^21^Université Pierre et Marie Curie, Paris, France; ^22^Pôle de Biologie, Hôpital Européen Georges Pompidou, AP-HP, Paris, France; ^23^Medical Oncology and Immunotherapy Division, University Hospital of Siena, Viale Bracci, 14, 53100, Siena, Italy; ^24^Medical Oncology and Immunotherapy, Department of Oncology, University Hospital of Siena, Istituto Toscano Tumori, Siena, Italy; ^25^Ludwig Center for Cancer Immunotherapy, Department of Immunology and Department of Medicine, Memorial Sloan-Kettering Cancer Center, New York, NY, USA

######## **Correspondence:** Laurence Zitvogel

^*^Nicolas Jacquelot, David Enot and Caroline Flament contributed equally to this work.

*Journal of Translational Medicine* 2016, **14(Suppl 1)**:O12

**Background:** The prognosis of a variety of human malignancies including melanoma is dictated by the abundance, spatial trafficking and quality of intratumoral T cell infiltrates [1]. Salerno *and al.* recently studied chemokine receptors (CC/CXCR) expression on Tumor-Infiltrating T cells (TILs) from several metastasis localizations and suggest a limited tissue-specific homing to human melanoma [2]. We studied the expression of these CC/CXCR on peripheral T cells from metastatic melanoma (MM) patients, during Ipilimumab therapy and healthy volunteers (HV). We found an orchestrated deregulation of these molecules according to the various sites of disease dissemination. Moreover, we discovered some putative biomarkers of response to Ipilimumab before and during the therapy such as the amount of CD4 and CD8 + CLA + effector memory T cells.

**Materials and methods:** We assessed this chemokine receptors profiling on 57 cryopreserved blood samples from stage III/IV melanoma patients and on 26 HV. Moreover, 47 patients treated with Ipilimumab and enrolled in four clinical centers (France, Germany, Italy and USA) were studied. We performed flow cytometric analysis of CD4 and CD8 T cells subsets based on their expression of CCR7 and CD45RA and then assessed, for each subset, the expression of CLA, CXCR5, CRTH2, CCR10, CD103, CCR9, CXCR4, CXCR3 and CCR6.

**Results:** Firstly, correlation matrices indicated similar chemokine receptor expression on CD4 and CD8 T cells, except for CXCR4 and CD103, and concordant patterns in naïve, effector and memory T cells. After classification of patients based on their metastasis localizations with skin and lymph node, lung or other distant organs with or without lung involvement, 62 parameters were found to differ significantly (FDR < 0.1) according to distinct localizations metastasis. Expression level normalized with respect to HV indicated that specific signatures could identify preferential metastasis localizations. Loss of CCR6 or CXCR3 (but not CLA) on circulating T cell subsets was associated with skin or lymph node metastases, loss of CXCR4, CXCR5 and CCR9 with lung involvement, and a raise in CCR10 or CD103 with widespread dissemination. High frequencies of CD8^+^CCR9^+^ TN correlated with prolonged OS (p < 10^−4^), while neutralizing the CCR9/CCL25 axis in mice stimulated tumor progression. Effector memory CD4^+^ and CD8^+^ expressing CLA increased upon CTLA4 blockade, both predicting disease control at 3 months of therapy in 47 MM.

**Conclusion:** We discovered an immune chemokine receptor profile which mirrors the metastatic pattern of the disease and highlighted new pharmacodynamic parameters during immune checkpoint blockade with Ipilimumab.

References

1. Erdag G, Schaefer JT, Smolkin ME, Deacon DH, Shea SM, Dengel LT, et al. Immunotype and immunohistologic characteristics of tumor-infiltrating immune cells are associated with clinical outcome in metastatic melanoma. Cancer Res. 2012;72:1070–80.

2. Salerno EP, Olson WC, McSkimming C, Shea S, Slingluff CL. T cells in the human metastatic melanoma microenvironment express site-specific homing receptors and retention integrins. Int J Cancer. 2014;134:563–74.

##### O13 Serum levels of PD1- and CD28-positive exosomes before Ipilimumab correlate with therapeutic response in metastatic melanoma patients

###### Passarelli Anna^1^, Tucci Marco^1^, Stucci Stefania^1^, Mannavola Francesco^1^, Capone Mariaelena^2^, Madonna Gabriele^2^, Ascierto Paolo Antonio^2^, Silvestris Franco^1^

####### ^1^Medical Oncology Unit, Department of Biomedical Sciences and Clinical Oncology, University of Bari ‘Aldo Moro’, Italy; ^2^Melanoma, Cancer Immunotherapy and Innovative Therapy Unit, INT “G. Pascale”, Napoli, Italy

######## **Correspondence:** Tucci Marco

*Journal of Translational Medicine* 2016, **14(Suppl 1)**:O13

**Background:** Although Ipilimumab (IPI) definitely improves the PFS and extends the OS in metastatic melanoma [1], the immunologic criteria for both evaluation and prediction of the therapeutic response are presently uncertain in clinical management of patients. Recently, both melanoma [2] and immune cells [3] have been described to release exosomes (Exo), namely endomysial microvesicles with antigenic and molecular features resembling the original phenotype pattern of the producing cell [4]. Thus, serum levels of Exo may indirectly reflect the response to IPI immunotherapy and detection of both CD28 and PD1 expressed by T cell-released Exo in sera of IPI-treated patients may identify the responders, suggesting Exo measurement as a potential tool to evaluate the immune response against melanoma.

**Materials and methods:** Sixty patients with advanced melanoma in treatment with IPI were enrolled. Sera were collected both before and after the four-month treatment, and Exo were purified by the “Total Exosome Isolation kit” (Invitrogen, Carlsbad, California). Thus, they were investigated by flow-cytometry in their expression of PD1, CD28, ICOS by gating the CD3^+^ cells and CD86 with the purpose to identify Exo released by dendritic cells (DEX). Therefore, median levels of PD1-, CD28- and ICOS-positive Exo were calculated both at baseline and longitudinally and compared with the therapeutic response by waterfall chart. Finally, both PFS and OS were calculated by Kaplan–Meier regression analyses.

**Results:** The therapeutic response to IPI as disease stability, partial or complete response, associated with PFS improvement and favourable trend of OS, was detected in 12 patients. Data from Exo investigation revealed higher expression of both PD1 and CD28 before IPI (p = 0.03; and 0.008 respectively), whereas the ICOS level expression remained mostly unchanged. By contrast, the major group of patients with disease progression showed minimal levels of either PD1-, or CD28-positive Exo. Interestingly, we found in IPI-responsive patients a quantitative increase of DEX after completion of the immunotherapy treatment (p < 0.01). Thus, we interpreted this finding as effect of the anti-melanoma immune activation.

**Conclusions:** Our data suggest that higher levels of PD1- and CD28-positive Exo in sera of melanoma patients before undergoing IPI treatment may predict the anti-melanoma immune activation induced by IPI and that the measurement of these molecular biomarkers could early identify the melanoma patients responsive to immunotherapy.

References

1. Hodi F.S et al. Improved Survival with Ipilimumab in Patients with Metastatic Melanoma. NEJM 2010.

2. Logozzi M et al. High Levels of Exosomes Expressing CD63 and Caveolin-1 in Plasma of Melanoma Patients. Plos One. 2009.

3. Segura E et al. Mature dendritic cells secrete exosomes with strong ability to induce antigen-specific effector immune responses. Blood Cells Mol Dis. 2005.

4. Viaud S et al. Dendritic Cell-Derived Exosomes for Cancer Immunotherapy: What’s Next? Cancer Res. 2010.

##### O14 Immunological prognostic factors in stage III melanomas

###### María Paula Roberti^1,2,3,*^, Nicolas Jacquelot^1,2,4,*^, David P Enot^3,5,*^, Sylvie Rusakiewicz^1,3,4^, Michaela Semeraro^1,3,6^, Sarah Jégou^24^, Camila Flores^3^, Lieping Chen^8^, Byoung S. Kwon^9,10^, Ana Carrizossa Anderson^11^, Caroline Robert^3,12,13^, Christophe Borg^14,15,16^, Benjamin Weide^27^, François Aubin^28^, Stéphane Dalle^17^, Michele Maio^25^, Jedd D. Wolchok^26^, Holbrook Kohrt^22^, Maha Ayyoub^1,2,3,4^, Guido Kroemer^3,5,7,18–21^, Aurélien Marabelle^1,3,4^, Andréa Cavalcanti^3,12,23^, Alexander Eggermont^3,*^, Laurence Zitvogel^1,2,3,4,*^

####### ^1^INSERM U1015, Gustave Roussy Cancer Institute, Villejuif, France; ^2^Gustave Roussy Cancer Campus, Villejuif, France; ^3^CIC Biothérapie IGR Curie CIC1428, Gustave Roussy Cancer Institute, Villejuif, France; ^4^University Paris Sud XI, Kremlin Bicêtre, Villejuif, France; ^5^Metabolomics and Cell Biology Platforms, Gustave Roussy Cancer Institute, Villejuif, France; ^6^Center of Clinical Investigation Hôpital Necker Enfants Malades, Paris, France; ^7^INSERM U1138, Centre de Recherche des Cordeliers, Paris, France; ^8^Department of Immunobiology, Yale School of Medicine, 10 Amistad Street, New Haven, Connecticut, 06519, USA; ^9^Cancer Immunology Branch, Division of Cancer Biology, National Cancer Center, Ilsan, Goyang, Gyeonggi, Korea; ^10^Section of Clinical Immunology, Allergy, and Rheumatology, Department of Medicine, Tulane University Health Sciences Center, New Orleans, Louisiana, USA; ^11^Evergrande Center for Immunologic Diseases and Ann Romney Center for Neurologic Diseases, Brigham and Women’s Hospital and Harvard Medical School, Boston, MA 02115, USA; ^12^Cancer Institute Gustave Roussy, Departments of Dermatology, Villejuif, France; ^13^Clinical Oncology, Melanoma Branch, Gustave Roussy Cancer Institute, Villejuif, France; ^14^Department of Medical Oncology, University Hospital of Besancon, 3 Boulevard Alexander Fleming, Besancon, F-25030, France; ^15^Clinical Investigational Centre, CIC-1431, University Hospital of Besançon, Besançon, France; ^16^INSERM U1098, University of Franche-Comté, Besançon, France; ^17^Centre Hospitalier Lyon-Sud, Hospices Civils de Lyon; and University Claude Bernard Lyon 1, Lyon, France; ^18^Equipe 11 labellisée par la Ligue contre le Cancer, Centre de Recherche des Cordeliers; Paris, France; ^19^Université Paris Descartes, Sorbonne Paris Cité; Paris, France; ^20^Université Pierre et Marie Curie, Paris, France; ^21^Pôle de Biologie, Hôpital Européen Georges Pompidou, AP-HP; Paris, France; ^22^Division of Oncology, Department of Medicine, Stanford University, Stanford, California, USA; ^23^Department of Surgery, Gustave Roussy Cancer Campus, Villejuif, France; ^24^Saint Antoine Hospital, INSERM ERL 1157-CNRS UMR 7203, Paris, France; ^25^Medical Oncology and Immunotherapy, Department of Oncology, University Hospital of Siena, Istituto Toscano Tumori, Siena, Italy; ^26^Ludwig Institute for Cancer Research, Memorial Sloan-Kettering Cancer Center, New York, Weill Cornell Medical College, New York, NY, USA; ^27^Division of Dermatooncology, Department of Dermatology, University Medical Center Tübingen, Germany; ^28^Université de Franche Comté, EA3181, SFR4234, Service de Dermatologie, Centre Hospitalier Universitaire (CHU), Besançon, France

######## **Correspondence:** Laurence Zitvogel

^*^María Paula Roberti, Nicolas Jacquelot, David P Enot, Alexander Eggermont and Laurence Zitvogel contributed equally to this work.

*Journal of Translational Medicine* 2016, **14(Suppl 1)**:O14

**Background:** Stage III melanomas (MM) require adequate adjuvant immunotherapy to prevent relapses. The magnitude of metastatic lymph node (mLN) invasion and a BRAF^V600^ activating mutation have a clinical significance; however, prognostic factors are awaited to optimize the clinical management of these patients.

**Materials and methods:** We performed a comprehensive immunophenotyping of 252 parameters/patient by flow cytometry and ELISA techniques, in paired blood and mLN performed in 39 metastatic melanomas. Wilcoxon signed-rank test was applied to assess differences in concentration between matching pairs of blood leukocytes and tumor infiltrated cells samples. Overall Survival (OS) and Relapse Free Survival (RFS) determined from the date of sampling were used as the primary end-points. Survival curves were estimated by the Kaplan–Meier product-limit method.

**Results:** We found immunotypes corresponding to clinical presentations. mLN extensive invasion was associated with lower levels of circulating CD4^+^ T cells and lower expressions of CD137L on CD3^−^CD56^−^ cells and PD1 on CD8^+^, in blood and TILs, respectively. BRAF mutation was associated with higher levels of CD45RA^+^ CD4^+^ TILs and lower expression of CD137L on circulating CD8^+^ and NK cells. High frequencies of tumoral CD45RA^+^CD4^+^ and CD3^−^CD56^−^ TILs were independent prognostic factors of short RFS, and remained significant after adjusting for mLN invasion, therefore adding prognostic value to local aggressiveness. The best predictive markers of OS after adjusting for significant clinical variables included NKG2D expression levels on intratumoral CTLs, intratumoral Tregs infiltration, and PD-L1 intensity on blood T cells. When combined with BRAF mutation, very high percentages of NKG2D^+^CD8^+^ TILs or low percentages of Tregs protected against the BRAF mutation-associated dismal prognosis. In blood, high expression levels of PD-L1 on CD4^+^ and CD8^+^ T lymphocytes were associated with a bad prognosis, also adding value to the BRAF mutational status.

**Conclusions:** Overall, when classifying our 252 immunological markers determined in blood and tumor beds according to their biological and clinical significance in stage III MM, we concluded that (i) both blood and tumor markers equally contributed to defining the prognosis, (ii) exhaustion markers markedly affected the clinical outcome and (iii) activation markers appeared less relevant for the functional study of tumor beds. Only six markers were found to be significant prognostic factors influencing RFS or OS in blood or tumor beds independently of clinicopathological parameters in multivariate Cox regression analyses. These immunotypes deserve to be followed in the course of the development of monoclonal antibody therapies.

### POSTER PRESENTATIONS

#### Molecular and immuno-advances

##### P1 Human melanoma cells resistant to B-RAF and MEK inhibition exhibit mesenchymal-like features

###### Anna Lisa De Presbiteris^1^, Fabiola Gilda Cordaro^1^, Rosa Camerlingo^2^, Federica Fratangelo^2^, Nicola Mozzillo^2^, Giuseppe Pirozzi^2^, Eduardo J. Patriarca^1^, Paolo A. Ascierto^2^, Emilia Caputo^1^

####### ^1^Institute of Genetics and Biophysics –I.G.B., A. Buzzati-Traverso–, CNR, Naples, Italy; ^2^Istituto Nazionale Tumori Fondazione G. Pascale, Naples, Italy

######## **Correspondence:** Anna Lisa De Presbiteris

*Journal of Translational Medicine* 2016, **14(Suppl 1)**:P1

**Background:** Combined B-RAF and MEK inhibition elicits rapid anti-tumor responses in the most patients with B-RAF (V600E) mutant metastatic melanoma. However, the vast majority of patients develop resistance to these agents [1]. Defining the phenotypic properties of melanoma cells resistant to single B-RAF and combined with MEK inhibition is critical for therapeutic exploitation.

**Materials and methods:** Melanoma cell lines, resistant to B-RAF inhibitors (B-RAFi) as single agent or combined with MEK inhibitors (MEKi), were selected by growing them in medium containing increased concentrations of B-RAF inhibitors (B-RAFi: GSK2118436) alone or combined MEK inhibitors (MEKi: GSK1120212). Morphological and phenotypic investigation was performed by light microscopy, immunofluorescence and flow cytometer analysis.

**Results:** Here, we detected a cell subset inside of three different melanoma cell lines, deriving from patients with B-RAF (V600E) mutant metastatic melanoma. This cell-subset was able to proliferate and forming colonies at low density in presence of single B-RAF inhibitors (B-RAFi) or combined with MEK inhibitors (MEKi), suggesting that drug treatment was unable to kill all the cells, but few of them continued to grow forming sizeable colonies as the ones observed from untreated cells.

We enriched this cell-subset, by growing them in drugs and we characterized them by measuring drug IC50 and examining the effect of the drug on the cell cycle, by flow cytometer.

Successively, we investigated their morphological and phenotypic properties. We found that melanoma cells, resistant to B-RAFi alone or combined with MEKi, exhibited a bigger cellular body, an increased inter-cellular space and the pseudopodia formation compared to their sensitive counterparts. FACS analysis of CD90 and CD324 markers revealed an increased expression of CD90 with a reduction of CD324 in resistant cells. In addition, the drug resistant cells were able to attach and spread on the plate, in a 3D culture system, while the sensitive ones were unable to do it. We also found a reorganization of the network of vimentin mainly localized at perinuclear sites in the drug resistant cells. All together these data supported a mesenchymal-like phenotype of the melanoma cells, resistant to B-RAFi alone or combined with MEKi.

**Conclusions:** These data suggest that melanoma cells contained a cell-subset with mesenchymal-like features that were resistant to B-RAFi and MEKi drugs [2]. Further characterization of these cells will help to define novel targets opening up the possibility to develop efficacious therapeutic strategies and contributing to overcome the big issue of drug resistance in melanoma.

References

1. Larkin J, Ascierto PA, Dréno B, Atkinson V, Liszkay G, Maio M, Mandalà M, Demidov L, Stroyakovskiy D, Thomas L, de la Cruz-Merino L, Dutriaux C, Garbe C, Sovak MA, Chang I, Choong N, Hack SP, McArthur GA, Ribas A. Combined vemurafenib and cobimetinib in BRAF-mutated melanoma. N Engl J Med. 2014;371(20):1867–76

2. Singh A, Settleman J. EMT, cancer stem cells and drug resistance: an emerging axis of evil in the war on cancer. Oncogene. 2010;29(34):4741–51.

##### P2 Anti-proliferative and pro-apoptotic effect of ABT888 on melanoma cell lines and its potential role in the treatment of melanoma resistant to B-RAF inhibitors

###### Federica Fratangelo^1^, Rosa Camerlingo^1^, Emilia Caputo^2^, Maria Letizia Motti^1,3^, Rosaria Falcone^1^, Roberta Miceli^1^, Mariaelena Capone^1^, Gabriele Madonna^1^, Domenico Mallardo^1^, Maria Vincenza Carriero^1^, Giuseppe Pirozzi^1^ and Paolo Antonio Ascierto^1^

####### ^1^IRCCS Istituto Nazionale Tumori “Fondazione G. Pascale”, Naples, Italy; ^2^ Institute of Genetics and Biophysics –I.G.B., CNR, Naples, Italy; ^3^ University ‘Parthenope’, Naples, Italy

######## **Correspondence:** Federica Fratangelo

*Journal of Translational Medicine* 2016, **14(Suppl 1)**:P2

**Background:** Despite novel therapeutic treatments developed in recent years, advanced melanoma patients continue to die. Therefore, the development of efficacious therapeutic strategies to prevent or delay the onset of resistance mechanism in melanoma, is fundamental. ABT888 is a potential anti-cancer drug being evaluated in multiple tumor types; it acts as a poly-(ADP–Ribose) polymerases (PARP) inhibitor, nuclear enzymes that repairs damage to DNA. Through inhibition of PARP, ABT888 helps to prevent DNA repair in cancer cells, leading to cell death [1]. In the current study, we tested the anti-proliferative and pro-apoptotic action of ABT888 on the melanoma cell line A375 resistant to B-RAF inhibitor (Dabrafenib) and on other melanoma cell lines.

**Materials and methods:** Cell viability of melanoma cell lines (A375_B-RAFi_R, SK-MEL-5, SK-MEL-2, M14, COPA-159, A375, LOXI-IMVI, LCP, SK-MEL-28, 397-MEL and WM-115) after 72 h treatment with increasing concentrations of ABT888 (5, 10, 25 and 50 μM), was evaluated by MTT assay; analysis of the apoptotic process was performed by the Annexin V-FITC assay, using flow cytometry. Cell extracts from SK-MEL-28 cell line were also analyzed by western blotting to evaluate the effect of ABT888 on the main proliferative signaling pathways.

**Results:** Treatment of melanoma cell lines with ABT888 caused inhibition of cell growth, which was statistically significant starting from 10 μM ABT888 (mean of 65 % of cell survival). In SK-MEL-28 melanoma cell lysates, ABT888 drug exhibited an anti-proliferative action by the inhibition of AKT as demonstrated by decreased levels of pAKT, while the levels of Cyclin D1 and c-Myc were not affected. In addition, 25 μM ABT888 treatment was able to induce apoptosis in the cells (average cell death equal to 34 %, by Annexin V-FITC assay), as confirmed by the presence of PARP-1 cleavage in SK-MEL-28 cell lysates. Furthermore, ABT888 showed its anti-proliferative and pro-apoptotic activity on A375_B-RAFi_R, by inhibition of cell growth (71 % of cell survival, following 10 μM ABT888 treatment) and induction of apoptosis (75 % of cell death, following 25 μM treatment).

**Conclusions:** In this study we observe that ABT888 exhibited its anti-proliferative and pro-apoptotic activity on melanoma cell lines. Interesting, we found that ABT888 was able to kill melanoma cells resistant to B-RAF inhibitor (Dabrafenib). This results suggest that ABT888 may represent a promising therapeutic approach for melanomas that have developed resistance against some molecular targeted inhibitors. Further studies will be performed to assess whether the combination of B-RAF and PARP inhibitors can prevent or delay the onset of resistance mechanism in melanoma.

Reference

1. Donawho CK, Luo Y, Luo Y, Penning TD, Bauch JL, Bouska JJ, Bontcheva-Diaz VD, Cox BF, DeWeese TL, Dillehay LE, Ferguson DC, Ghoreishi-Haack NS, Grimm DR, Guan R, Han EK, Holley-Shanks RR, Hristov B, Idler KB, Jarvis K, Johnson EF, Kleinberg LR, Klinghofer V, Lasko LM, Liu X, Marsh KC, McGonigal TP, Meulbroek JA, Olson AM, Palma JP, Rodriguez LE, Shi Y, Stavropoulos JA, Tsurutani AC, Zhu GD, Rosenberg SH, Giranda VL, Frost DJ. ABT-888, an orally active poly (ADP-Ribose) polymerase inhibitor that potentiates DNA-damaging agents in preclinical tumor models. Clin Cancer Res. 2007;13(9):2728–37.

##### P3 Involvement of the l-cysteine/CSE/H_2_S pathway in human melanoma progression

###### Elisabetta Panza^1^, Paola De Cicco^1^, Chiara Armogida^1^, Giuseppe Ercolano^1^, Rosa Camerlingo^2^, Giuseppe Pirozzi^2^, Giosuè Scognamiglio^3^, Gerardo Botti^3^, Giuseppe Cirino^1^, Angela Ianaro^1^

####### ^1^Department of Pharmacy, University of Naples Federico II, Naples, Italy; ^2^Department of Experimental Oncology, Istituto Nazionale Tumori Fondazione “G. Pascale” Naples, Italy; ^3^Unit of Pathology, Istituto Nazionale per lo Studio e la cura dei tumori “Fondazione G. Pascale” IRCCS, Naples, Italy; ^4^Department of Physiology & Pharmacology, University of Calgary, Calgary, Alberta, Canada

######## **Correspondence:** Angela Ianaro

*Journal of Translational Medicine* 2016, **14(Suppl 1)**:P3

**Background:** Cutaneous malignant melanoma is the most aggressive form of skin cancer, with a high mortality rate. Various treatments for malignant melanoma are available, but due to the development of multi-drug resistance, current or emerging therapies have a relatively low success rates. This emphasizes the importance of discovering new compounds that are both safe and effective against melanoma [1]. In the last few years, numerous physiological and pathophysiological roles have been proposed for the gasotransmitter hydrogen sulfide (H_2_S), along with a plethora of cellular and molecular targets. Endogenously, H_2_S is produced as a metabolite of homocysteine (Hcy) by cystathionine β-synthase (CBS), cystathionine γ-lyase (CSE), and 3-mercaptopyruvate sulfurtransferase (3-MST) [2]. A number of studies have investigated the role of H_2_S in triggering cell death and evidence has been presented that this gas can exert both pro- and anti-apoptotic activity in cultured cells [3, 4]. Recently, CBS has emerged as a potential therapeutic target in both colon cancer and ovarian cancer [5, 6]. The aim of our study was to evaluate the role of the metabolic H_2_S pathway in human melanoma.

**Methods:** We utilized cell culture, immunohistochemistry, molecular biology techniques and mice melanoma model.

**Results:** Using immunohistochemistry we demonstrated the presence of the l-cysteine/CSE/H_2_S pathway in human melanoma specimens and provided evidence that CSE expression was highest in primary tumors while decreased in the metastatic lesions and it was almost silent in non lymph node metastases. Conversely, CBS did not appear to play an important role in melanoma. The role of CSE and the downstream signal transduction were investigated by using several human melanoma cell lines. The primary role played by CSE, already revealed by the human study, was confirmed by the finding that the over-expression of CSE induced spontaneous apoptosis of human melanoma cells (30 %; *P* < *0.001vs* mock transfected cells). The same effect was achieved by using different H_2_S donors and, among them, the most active resulted to be diallyl trisulfide (DATS) (IC_50_ = 89 μM). The main pro-apoptotic mechanisms involved were suppression of nuclear factor-κB activity and inhibition of AKT and ERK1/2 pathways. A proof of concept was obtained in vivo by using a murine melanoma model. In fact, either l-cysteine, the CSE substrate, or DATS inhibited tumor growth by 51 % and by 67 % respectively *(P* < 0.001, n = 8).

**Conclusion:** In conclusion, this work establishes that the l-cysteine/CSE/H_2_S pathway is involved in human melanoma and provide the fundamentals to exploit a possible therapeutic/diagnostic use in this aggressive disease.

References

1. Chinembiri TN, du Plessis L, Gerber M, Hamman JH, du Plessis J. Review of natural compounds for potential skin cancer treatment. Molecules. 2014;19:11679–721.

2. Sen U, Sathnur PB, Kundu S, Givvimani S, Coley DM, Mishra PK, et al. Increased endogenous H_2_S generation by CBS, CSE, and 3MST gene therapy improves ex vivo renovascular relaxation in hyperhomocysteinemia. Am J Physiol Cell Physiol. 2012;303(1):C41–51.

3. Hu LF, Wong PT, Moore PK, Bian JS. Hydrogen sulfide attenuates lipopolysaccharide-induced inflammation by inhibition of p38 mitogen-activated protein kinase in microglia. J Neurochem. 2007;100:1121–8.

4. Taniguchi S, Kang L, Kimura T, Niki I. Hydrogen sulphide protects mouse pancreatic β-cells from cell death induced by oxidative stress, but not by endoplasmic reticulum stress. Br J Pharmacol. 2011;162:1171–8.

5. Bhattacharyya S, Saha S, Giri K. Cystathionine beta-synthase (CBS) contributes to advanced ovarian cancer progression and drug resistance. PLoS One. 2013;8: e79167.

6. Szabo C, Coletta C, Chao C, Modis K, Szczesny B, Papapetropoulos A, Hellmich MR. Tumor-derived hydrogen sulfide, produced by cystathionine-beta-synthase, stimulates bioenergetics, cell proliferation, and angiogenesis in colon cancer. Proc Natl Acad Sci USA. 2013;110:12474–9.

##### P4 Cancer stem cell antigen revealing pattern of antibody variable region genes were defined by immunoglobulin repertoire analysis in patients with malignant melanoma

###### Beatrix Kotlan^1,2^, Gabriella Liszkay^3^, Miri Blank^4^, Timea Balatoni^3^, Judit Olasz^5^, Emil Farkas^6^, Andras Szollar^6^, Akos Savolt^6^, Maria Godeny^7^, Orsolya Csuka^5^, Szabolcs Horvath^2^, Klara Eles^2^, Yehuda Shoenfeld^4^ and Miklos Kasler^8^

####### ^1^Molecular Immunology and Toxicology, National Institute of Oncology, Budapest, Hungary; ^2^Center of Surgical and Molecular Tumorpathology, National Institute of Oncology, Budapest, Hungary; ^3^Oncodermatology, National Institute of Oncology, Budapest, Hungary; ^4^Zabludowitz Center for Autoimmune Diseases, Sheba Medical Center affiliated to Sackler Faculty of Medicine, Tel-Aviv University, Tel Aviv, Israel; ^5^Pathogenetics, National Institute of Oncology, Budapest, Hungary; ^6^Oncosurgery, National Institute of Oncology, Budapest, Hungary; ^7^Department of Radiological Diagnostics, National Institute of Oncology, Budapest, Hungary; ^8^Board of Directors, National Institute of Oncology, Budapest, Hungary

######## **Correspondence:** Beatrix Kotlan

*Journal of Translational Medicine* 2016, **14(Suppl 1)**:P4

**Background:** Revealing cancer stem cells (CSC) is a major issue for effective cancer therapeutics. We postulated that an immunoglobulin (Ig) repertoire analysis and investigating freshly removed cancerous tissues would serve this aim. Our focus was kept on the natural humoral immune response, having the capacity to recognize modified oligosaccharide expression patterns, characteristic for cancer.

**Materials and methods:** In the course of a tumorimmunological study primary and metastatic tumors as well as peripheral blood samples of patients with malignant melanoma (n = 123) were investigated in terms of antibody repertoire. Heavy (VH) and light chain (VL) Ig variable region gene fragments were amplified by polymerase chain reaction and VH and VL were adjoined by a [(Gly)4Ser]_3_ linker for the single chain Fv (scFv) construct. Two technical approaches for scFv phage display library (1) generation were standardised. A column purification process was used to get enriched immunoglobulins for binding assays (2). Comparative DNA sequence analyses and antibody phage enzyme-linked immunosorbence assay (ELISA) with native membrane preparations were done.

**Results:** The expressed immunoglobulin variable region gene usage in the VH3 family represented the VH3-23 and VH3-21 overexpression, and revealed that sequences were frequently in a germline configuration with a low replacement to silent ratio of mutations. Among the representative Ig clusters a cancer stem cell-associated CD34 and GD3 disialylated glycosphingolipid-targetting pattern could be defined. Using native membrane preparations and primary cultures from the tumor was found to be ideal to reveal novel cancer cell characteristics and antibody fragments with CSC binding potential.

**Conclusions:** Our technology helps to understand and harness the miscellaneous field of human antibody repertoire composition in malignant diseases, compared to autoimmune and normal conditions. The present data show unique potentials of tumor infiltrating B cell-derived antibodies to reveal cancer stem cell characteristics and would serve for novel diagnostic and therapeutic developments.

**Acknowledgements**: The Harry J. Lloyd Charitable Trust Melanoma Research Award (2010), OTKA T048933, Fulbright No1206103, No1214104 Grants given to B. Kotlan are acknowledged.

References

1. Kotlan B, Glassy MC. Antibody phage display. Overview of a powerful technology that has quickly translated to the clinic. In: Aitken R, editor. Methods in Molecular Biology. Humana Press Inc.,U.S. Series: Methods in Molecular Biology No. 562. 2009. p. 1–16

2. Shoenfeld Y, Blank M, Branch DR, Vassilev T, Käsermann F, Bayry J, Kaveri S, Simon HU. IVIG pluripotency and the concept of Fc-sialylation: challenges to the scientist. Nat Rev Immunol. 2014;14(5):349.

##### P5 Upregulation of Neuregulin-1 expression is a hallmark of adaptive response to BRAF/MEK inhibitors in melanoma

###### Debora Malpicci^1^, Luigi Fattore^2^, Susan Costantini^2^, Francesca Capone^2^, Paolo Antonio Ascierto^2^, Rita Mancini^3,4^, Gennaro Ciliberto^2^

####### ^1^Dipartimento di Medicina Sperimentale e Clinica, Università degli Studi di Catanzaro “Magna Graecia”, Catanzaro, Italy, ^2^Istituto Nazionale per lo Studio e la Cura dei Tumori “Fondazione G. Pascale”, Naples, Italy Rome, Italy ^3^ Dipartimento di Chirurgia “P. Valdoni”, Sapienza Università di Roma, Rome, Italy, ^4^ Dipartimento di Medicina Clinica e Molecolare, Sapienza Università di Roma, Rome, Italy

######## **Correspondence:** Debora Malpicci

*Journal of Translational Medicine* 2016, **14(Suppl 1)**:P5

**Background:** Understanding the mechanisms which contribute to the development of resistance to BRAF and MEK inhibitors in melanoma may lead to new target discovery and to the development of more effective combination therapies. Recently the ErbB3 receptor has been shown by us and by others to act as a central node in the promotion of adaptive survival of BRAF mutated melanoma cells early upon drug exposure. Two alternative modes of ErbB3 activation have been proposed: the first involves FOXD3-dependent transcriptional activation of ErbB3 and increased cell sensitization to exogenous ErbB3 ligand neuregulin (NRG1); the second increased NRG1 production and autocrine activation of ErbB3. The present study was undertaken to provide better insights into these two alternative mechanisms.

**Materials and methods:** BRAF-mutated melanoma cell lines M14, A375 and LOX-IMVI (BRAF V600E), WM115 and WM266 (BRAF V600D) were exposed to BRAFi and/or MEKi treatments at different times. mRNA expression levels were assessed by Real Time PCR. Western blot analysis was performed on total protein extracts from untreated and treated cells. NRG-1 in melanoma cell media was assessed using Bio-Plex Pro NRG-1 Assays (Bio-Rad).

**Results:** Melanoma cells were treated with BRAFi and/or MEKi for different times (2–72 h) and changes in gene expression levels of NRG-1, ErbB3 and FOXD3 were determined. We observed two different patterns. In the first case exemplified by M14 and A375 cells we detected simultaneous increases in mRNA levels of NRG-1, FOXD3 and (to a lower extent) ErbB3 only at late times after drug exposure. Surprisingly, the late increase of NRG-1 mRNA expression was not accompanied by increase in the secretion of the cytokine in the cell medium. In contrast in WM266 cells a sharp increase of NRG1 and to a lower extent of FOXD3 without changes in ErbB3 was observed early after drug exposure. In line with this last finding we were able to detect up to thousand –fold increase in secreted neuregulin-1 in cell media of WM266 shortly after drug exposure.

**Conclusions:** Our preliminary results show that a constant feature of melanoma cells exposed to drugs that inhibit the BRAF/MEK axis is the increased expression of NRG-1, albeit with different kinetics in different cells. Interestingly increased expression is not always accompanied by increased cytokine secretion. We are currently performing transfections with NRG-1 promoter luciferase fusion in order to provide new insights on the transcriptional regulation of this gene upon drug exposure.

##### P6 HuR positively regulates migration of HTB63 melanoma cells

###### Farnaz Moradi^1^, Pontus Berglund^1^, Karin Leandersson^2^, Rickard Linnskog^1^, Tommy Andersson^1^, Chandra Prakash Prasad^1^

####### ^1^Department of Cell and Experimental Pathology, Lund University, Lund, Sweden; ^2^Center for Molecular Pathology, Institution of Translational Medicine, Lund University, Lund, Sweden

######## **Correspondence:** Chandra Prakash Prasad

*Journal of Translational Medicine* 2016, **14(Suppl 1)**:P6

**Background:** HuR is a ubiquitously expressed member of the Hu family that contains three RNA-recognition motifs with the ability to bind adenylate- and uridylate-rich elements (AREs) in target mRNA [1]. These AREs are usually located in the 3′UTR of target transcripts, and the binding of HuR largely results in mRNA stabilization and/or increased translation [2, 3]. Furthermore, aberrant HuR expression has been reported in malignancies such as colon, breast and prostate cancer [4, 5, 6,]. Among the many established HuR targets are the transcripts of genes involved in cancer and inflammation, including COX-2 and MMP-9 [7]. Using cutting-edge PAR-CLIP technique, Lebedeva et al. demonstrated RBP HuR binding sites in WNT5A mRNA, in the transcriptome of HeLa cells [8]. Overexpression of WNT5A plays a significant role in melanoma cancer progression; however, the mechanism(s) involved remains unknown.

In the present study, we investigated if RNA-binding protein HuR interacts with WNT5A mRNA thereby regulating its expression. We also delineated the functional consequences of such interaction by studying its impact on melanoma cell migration.

**Materials and methods:** To identify HuR–WNT5A mRNA interaction, HuR-RNA binding protein immunoprecipitation (RIP) assay and WNT5A 3′UTR luciferase assay were carried out. HuR-WNT5A association studies were investigated via siRNA knockdown of HuR and short-term treatment with the low molecular weight HuR inhibitor, MS-444. To investigate functional consequences of HuR-WNT5A interactions on melanoma cell migration, we performed trans-well cell migration assays.

**Results:** We selected HTB63 as a model cell line for our study for two reasons. Firstly, it expresses WNT5A protein endogenously and secondly, according to HOPP algorithm it is characterized as invasive cell phenotype. We found that endogenous expression of WNT5A correlates with levels of active HuR in these cells. We further demonstrated that HuR protein binds to 3′ UTR of WNT5A mRNA in HTB63 cells. HuR inhibition either via siRNA knockdown or by MS-444 treatment resulted in significant inhibition of WNT5A mRNA and protein levels in these cells. Finally, we demonstrated that MS-444 treatment significantly inhibited migration of HTB63 cells in WNT5A-dependent fashion, as MS-444-induced inhibition was restored by the addition of recombinant WNT5A.

**Conclusions:** HuR protein binds to 3′UTR of WNT5A mRNA and governs HTB63 melanoma cell migration via up-regulating WNT5A protein expression.

References

1. Myer VE, Fan XC, Steitz JA. Identification of HuR as a protein implicated in AUUUA-mediated mRNA decay. The EMBO J. 1997;16:2130–9.

2. Meisner NC, Filipowicz W. Properties of the regulatory RNA-binding protein HuR and its role in controlling miRNA repression. Adv Exp Med Biol. 2010;700:106–23.

3. Peng SS, Chen CY, Xu N, Shyu AB. RNA stabilization by the AU-rich element binding protein, HuR, an ELAV protein. The EMBO J. 1998;17:3461–70.

4. Denkert C, Koch I, Von Keyserlingk N, Noske A, Niesporek S, Dietel M, Weichert W. Expression of the ELAV-like protein HuR in human colon cancer: association with tumor stage and cyclooxygenase-2. Mod Pathol. 2006;19:1261–9.

5. Hinman MN, Lou H. Diverse molecular functions of Hu proteins. Cell Mol Life Sci. 2008;65:3168–81.

6. Niesporek S, Kristiansen G, Thoma A, Weichert W, Noske A, Buckendahl AC, Jung K, Stephan C, Dietel M, Denkert C. Expression of the ELAV-like protein HuR in human prostate carcinoma is an indicator of disease relapse and linked to COX-2 expression. Int J Oncol. 2008;32:341–7.

7. Srikantan S, Gorospe M. HuR function in disease. Front Biosci. 2012;17:189–205.

8. Lebedeva S, Jens M, Theil K, Schwanhausser B, Selbach M, Landthaler M, Rajewsky N. Transcriptome-wide analysis of regulatory interactions of the RNA-binding protein HuR. Mol Cell. 2011;43:340–52.

##### P7 Prolyl 4- (*C*-*P4H*) hydroxylases have opposing effects in malignant melanoma: implication in prognosis and therapy

###### Cristiana Lo Nigro^1^, Laura Lattanzio^1^, Hexiao Wang^2^, Charlotte Proby^2^, Nelofer Syed^3^, Marcella Occelli^4^, Carolina Cauchi^4^, Marco Merlano^4^, Catherine Harwood^5^, Alastair Thompson^2^, Tim Crook^2^

####### ^1^Laboratory of Cancer Genetics and Translational Oncology, S. Croce & Carle Teaching Hospital, Cuneo, Italy; ^2^Dundee Cancer Center, University of Dundee, Ninewells Hospital and Medical School, Dundee, Scotland, UK; ^3^Faculty of Medicine, Imperial College London, London, UK; ^4^Medical Oncology, Oncology Department, S. Croce & Carle Teaching Hospital, Cuneo, Italy; ^5^Barts and the London School of Medicine and Dentistry, Queen Mary University of London, London, UK

######## **Correspondence:** Cristiana Lo Nigro

*Journal of Translational Medicine* 2016, **14(Suppl 1)**:P7

**Background:** Alterations in the properties of the basement membrane are a recognized feature of cancer cells. Collagen, the principal structural component, must undergo appropriate post-translational processing for efficient function and this requires prolyl hydroxylation, catalyzed by the prolyl 3- (*C*-*P3H*) and prolyl 4- (*C*-*P4H*) hydroxylases, which have multiple family members in the human genome.

**Materials and methods:** Using qPCR, western blotting and pyrosequencing, we have investigated the expression and transcriptional regulation, by means of CpG Island methylation, in the *C*-*P4H* family in melanoma cell lines and in clinical cases of melanoma including primary and metastatic lesions, both in tissues and in sera.

To seek mechanistic correlates of the proposed differences between *P4HA1*/*P4HA2* and *P4HA3*, we modulated expression of *P4HA2* and *P4HA3* in melanoma cell lines using ectopic expression and knock-down.

**Results:** We show that the *C*-*P4HsP4HA1* and *P4HA2* are invariably unmethylated and often over-expressed in melanoma, whereas *P4HA3* is subject to methylation-dependent transcriptional silencing in primary and particularly metastatic melanoma. Ectopic expression of *P4HA2* and *P4HA3* has opposing effects on melanoma growth, *P4HA2* promoting and *P4HA3* inhibiting proliferation of melanoma cell lines. Pharmacological inhibition of *C*-*P4H* has anti-proliferative effects on melanoma cells irrespective of *P4HA3* status and results in dose-dependent G2-M cell cycle block. Detection of methylated *P4HA3* in genomic DNA from *t*he peripheral blood of melanoma patients is a sensitive and specific biomarker of metastatic melanoma.

**Conclusions:** Our data thus identify the *C*-*P4H* family as potentially important modulators of melanoma growth and imply that pharmacological inhibitors of collagen prolyl 4-hydroxylation merit further investigation as novel anti-melanoma therapeutics. Further, the frequent methylation of *P4HA3* in metastatic melanoma suggests that *P4HA3* may have utility as a tissue and serum biomarker of metastatic disease.

##### P8 Urokinase receptor antagonists: novel agents for the treatment of melanoma

###### Maria Letizia Motti^1,2^, Katia Bifulco^1^, Vincenzo Ingangi^1^, Michele Minopoli^1^, Concetta Ragone^1^, Federica Fratangelo^1^, Antonello Pessi^3^, Gennaro Ciliberto^1^, Paolo Antonio Ascierto^1^, Maria Vincenza Carriero^1^

####### ^1^IRCCS Istituto Nazionale Tumori “Fondazione G. Pascale”, Naples, Italy; ^2^University ‘Parthenope’, Naples, Italy; ^3^PeptiPharma, Viale Città D’Europa 679, 00144, Rome, Italy

######## **Correspondence:** Maria Vincenza Carriero

*Journal of Translational Medicine* 2016, **14(Suppl 1)**:P8

**Background:** Melanoma has a high metastatic potential due to its tendency to metastasize through blood and lymphatic vessels. However, to date, effective treatments for metastatic melanoma are lacking and the prognosis of these patients remains very poor. One major goal of melanoma research is to identify new molecular targets for the development of novel treatments aimed to counteract intra and/or extravasation of melanoma cells. Several studies support the role of the urokinase-type plasminogen activator receptor (uPAR) in melanoma as increased expression of uPAR has been found to be associated with poor prognosis and increased risk of metastasis [1, 2]. uPAR forms complex signaling units with trans-membrane receptors, including G protein coupled Formyl Peptide Receptors (FPR)s [3]. These complexes lead to the activation of signaling cascades controlling cell migration as well as growth and survival. The uPAR_84–95_ chemotactic sequence is required to induce cell motility by interacting with FPRs that, in turn, signal through the integrins [4, 5]. By a drug-design approach based on the conformational analysis of the uPAR chemotactic sequence, we have developed a family of peptides which inhibit cell migration by preventing uPAR/FPR-1 interaction [6, 7, 8]. This study was aimed to investigate the contribution of uPAR on the capability of melanoma cells to cross endothelial barriers during metastasis with the aim to block this process.

**Materials and methods:** Human melanoma A375 and M14 cell lines expressing different levels of uPAR and comparable levels of FPR-1 on cell surface, were analyzed for their ability to move toward chemotactic gradients, to invade extra-cellular matrix and to disrupt endothelial monolayers using the Real Time Cell Analysis System and the xCELLigence technology [9].

**Results:** We found that uPAR levels positively correlate with motility, invasiveness and capability of human melanoma cells to cross endothelial monolayers. The important role of the uPAR_84–95_ sequence in determining invasive ability of melanoma cells was confirmed by the finding that specific antibodies recognizing uPAR_84–95_ as well as peptide inhibitors of the uPAR_84–95_/FPR1 interaction abrogate melanoma cell ability to move toward chemotactic gradients, to invade basal membranes and to cross endothelial monolayers.

**Conclusions:** These findings identify uPAR as a novel prognostic marker, the FPR as novel therapeutic target in melanoma and suggest that inhibitors of the uPAR_84–95_/FPR1 interaction may be useful for the treatment of metastatic melanoma.

References

1. Besch R1, Berking C, Kammerbauer C, Degitz K. Inhibition of urokinase-type plasminogen activator receptor induces apoptosis in melanoma cells by activation of p53. Cell Death Differ. 2007;14:818–29.

2. Nip J, Rabbani SA, Shibata HR, Brodt P. Coordinated expression of the vitronectin receptor and the urokinase-type plasminogen activator receptor in metastatic melanoma cells. J Clin Invest.1995;95: 2096–103.

3. Carriero MV, Stoppelli MP. The urokinase-type plasminogen activator and the generation of inhibitors of urokinase activity and signaling. Curr Pharm Des. 2011;17:1944–61. Review.

4. Resnati M, Pallavicini I, Wang JM, Oppenheim J, Serhan CN, Romano M, Blasi F. The fibrinolytic receptor for urokinase activates the G protein-coupled chemotactic receptor FPRL1/LXA4R. Proc Natl Acad Sci USA. 2002;99:1359–64.

5. Gargiulo L, Longanesi-Cattani I, Bifulco K, Franco P, Raiola R, Campiglia P, Grieco P, Peluso G, Stoppelli MP, Carriero MV. Cross-talk between fMLP and vitronectin receptors triggered by urokinase receptor-derived SRSRY peptide. J Biol Chem. 2005;280:25225–32.

6. Carriero MV, Longanesi-Cattani I, Bifulco K, Maglio O, Lista L, Barbieri A, Votta G, Masucci MT, Arra C, Franco R, De Rosa M, Stoppelli MP, Pavone V. Structure-based design of an urokinase-type plasminogen activator receptor-derived peptide inhibiting cell migration and lung metastasis. Mol Cancer Ther. 2009;8:2708–17.

7. Bifulco K, Longanesi-Cattani I, Liguori E, Arra C, Rea D, Masucci MT, De Rosa M, Pavone V, Stoppelli MP, Carriero MV. A urokinase receptor-derived peptide inhibiting VEGF-dependent directional migration and vascular sprouting.Mol Cancer Ther. 2013;12:1981–93.

8. Carriero MV, Bifulco K, Minopoli M, Lista L, Maglio O, Mele L, Di Carluccio G, De Rosa M, Pavone V. UPARANT: a urokinase receptor-derived peptide inhibitor of VEGF-driven angiogenesis with enhanced stability and in vitro and in vivo potency. Mol Cancer Ther. 2014;13:1092–104.

9. Bifulco K, Votta G, Ingangi V, Di Carluccio G, Rea D, Losito S, Montuori N, Ragno P, Stoppelli MP, Arra C, Carriero MV. Urokinase receptor promotes ovarian cancer cell dissemination through its 84–95 sequence. Oncotarget. 2014;5:4154–69.

##### P9 Exosomes released by melanoma cell lines enhance chemotaxis of primary tumor cells

###### Francesco Mannavola, Stella D’Oronzo, Claudia Felici, Marco Tucci, Antonio Doronzo, Franco Silvestris

####### Medical Oncology Unit, University of Bari ‘Aldo Moro’, Bari, Italy

######## **Correspondence:** Francesco Mannavola

*Journal of Translational Medicine* 2016, **14(Suppl 1)**:P9

**Background:** Exosomes (Exo) are nanovesicles produced by tumor cells that regulate the intercellular signals leading to cell proliferation and spreading to distant sites [1]. Variable amounts of Exo produced within the tumor microenvironment by both malignant and accessory cells also participate to enhance both migration and invasiveness [2] and recent studies support the role of Exo in tumor progression of several cancer models [3, 4]. However, their effect in melanoma progression has not been completely elucidated [5] and we explored here their chemotactic activity in vitro.

**Materials and methods:** Exo were purified by ultracentrifugation from culture medium of primary (LCP) and metastatic (LCM) melanoma cell lines. The ability of LCP and LCM to migrate was explored either in presence of Exo stimulation or not, using 8 μm trans-well chambers. Briefly, the upper chambers were filled with 10^4^ cells in 1 % fetal bovin serum (FBS) Exo-free medium, whereas the lower chambers were filled with the same medium supplemented with LCP- or LCM-Exo at different concentrations (10–50 μg/ml). Cells entrapped within the membrane were visualized after nuclei counterstaining with DAPI and counted at 40× magnification in at least five microscopic fields. Mann–Whitney test was used for statistical analyses.

**Results:** Not-stimulated LCP and LCM showed a similar migratory behaviour (13.5 ± 2.3 and 16.3 ± 2.6 cells/field, respectively) that, however, was significantly increased by stimulation with homologous Exo. In particular, LCP- and LCM-Exo stimulation at 10 μg/ml produced a 2.3- and 2.7-fold-increase of the migratory capacity as compared to the corresponding untreated cells (p = 0.01) and this effect was apparently dependent on Exo concentration (p < 0.05). Furthermore, the stimulation by Exo derived from the metastatic LCM cell line provoked an increased number of migrated LCP cells with respect to both untreated cells (4.47, 5.52 and 7.96-fold increase at 10, 20 and 50 μg/ml, respectively) and LCP-Exo stimulated cells (1.96, 2.19 and 2.28-fold increase, respectively) (p < 0.0002).

**Conclusion:** Although preliminary, our data appear to support the hypothesis that Exo released by melanoma cells enhance the chemotactic activity of primary tumor cells and that this effect is mainly evident when using line-related or homologous Exo. Therefore, it is suggestive that they may have a role in tumor progression in melanoma in particular in favoring a metastatic phenotype.

References

1. Akers JC et al. Biogenesis of extracellular vesicles (EV): exosomes, microvesicles, retrovirus-like vesicles, and apoptotic bodies. J Neurooncol. 2013.

2. Rodrigues CA et al. Exosome in Tumour Microenvironment: Overview of the Crosstalk between Normal and Cancer Cells. Biomed Res Int. 2014.

3. Ye SB et al. Tumor derived exosomes promote tumor progression and T cell dysfunction through the regulation of enriched exosomal microRNAs in human nasopharyngeal carcinoma. Oncotarget. 2014.

4. Harris DA et al. Exosomes Released from Breast Cancer Carcinomas Stimulate Cell Movement. Plos One. 2015.

5. Hood JL, San RS, Wickline SA. Exosomes released by melanoma cells prepare sentinel lymph nodes for tumor metastasis. Cancer Res. 2011.

##### P10 New insights in mitochondrial metabolic reprogramming in melanoma

###### Anna Ferretta^1,2^, Gabriella Guida^2^, Stefania Guida^2^, Imma Maida^2^, Tiziana Cocco^2^, Sabino Strippoli^1^, Stefania Tommasi^1^, Amalia Azzariti^1^, Michele Guida^1^

####### ^1^Istituto Tumori “Giovanni Paolo II” I.R.R.C.S., Istituto di ricovero e cura a carattere scientifico, Bari, Italy; ^2^Dipartimento di Scienze Mediche di Base, Neuroscienze e Organi di Senso, Universita degli Studi di Bari, Bari, Italy

######## **Correspondence:** Gabriella Guida

*Journal of Translational Medicine* 2016, **14(Suppl 1)**:P10

**Background:** Metabolic reprogramming is a peculiarity of tumoral cells. Its role in melanoma has been described but molecular actors, the influence of BRAF mutations on cell metabolism and molecules involved in bioenergetic transformation, have not been clearly defined.

**Materials and methods:** Energetic metabolism of melanoma cell lines with different BRAF mutational status (Table 2) was studied through the evaluation of oxygen consuption, ATP, lactate and ROS levels. Western blot analysis of subunits of mitochondrial respiratory chain (17 kDa, Cox IV), porin (marker for mitochondrial matrix), lactate carriers (MCT1, MCT4) and MITF were also performed. Furthermore, mRNA levels of regulators of mitochondrial biogenesis (PGC-1alpha, PGC-1beta), their targets (TFAM, cyt c) and HIF-1alpha were evaluated.

**Table 2:** Energetic metabolism of melanoma cell lines with different BRAF mutational status

Cell line	Origin	NRAS	BRAF exon 15
HBL	Metastasis	WT/WT	WT/WT
LND1	Metastasis	WT/WT	WT/WT
hmel1	Metastasis	WT/WT	V600K/WT
hmel9	Primary melanoma	WT/WT	V600R/V600R
hmel11	Primary melanoma	WT/WT	V600R/WT
M3	Metastasis	WT/WT	V600E/V600E

**Results:** BRAFV600 cell lines showed lower levels of MITF, PGC-1alpha/PGC-1beta and a downregulation of oxidative metabolism related to a low oxygen consumption and to a higher glycolytic ATP production, compared to BRAFwt cell lines. These latter showed an increased expression of HIF-1alpha (50–200 %), MCT4 (150 %) and extracellular lactate (about 20 %).

**Conclusions:** Our results highlight the existence of a specific metabolic phenotype related to BRAF mutational status. These data demonstrated the possibility to take into account drugs acting on cellular metabolism for the treatment of metastatic melanoma, in order to support the activity of anti BRAF/antiMEK drugs or to overcome resistance mechanism.

##### P11 Lenalidomide restrains the proliferation in melanoma cells through a negative regulation of their cell cycle

###### Stella D’Oronzo, Anna Passarelli, Claudia Felici, Marco Tucci, Davide Quaresmini, Franco Silvestris

####### Medical Oncology Unit, University of Bari ‘Aldo Moro’, Bari, Italy

######## **Correspondence:** Stella D’Oronzo

*Journal of Translational Medicine* 2016, **14(Suppl 1)**:P11

**Background:** Immunotherapy is presently providing favourable clinical responses in metastatic melanoma (MM) and appears as innovative approach to be applied to other solid tumors. However, the therapeutic response is temporary and additional therapeutic approaches may extend its effectiveness and duration. Lenalidomide (Len) is an immunomodulant drug (IMiD) showing anti-inflammatory and anti-tumor properties in several hematologic disorders [1], as well as anti-melanoma activity in phase 2/3 clinical trials in MM [2]. Furthermore, in association with Sorafenib Len exerts a defined anti-angiogenic effect in a human ocular melanoma xenograft model [3]. As IMiD, Len activates the CD28 checkpoint expressed by T cells and drives the NF-kB activation downstream the p21 cell cycle negative regulator, while increasing the T-cell cytotoxicity. This activity occurs by overcoming the inhibition of CTLA4 [4]. Therefore, this study explores the molecular activity of Len against melanoma cells for future translation to clinical treatment of melanoma.

**Materials and methods:** Three melanoma cell lines (A375, SKMEL28, WN266) were first cultured in serum-free medium to synchronize the cell cycle and then for 24 h in complete medium, supplemented with Len at different concentrations from 0.01 to 100 μM. A standard curve of cytotoxicity was assessed to measure the IC^50^ whereas both viability and proliferation of melanoma cells were evaluated by MTT assay. The effect of Len on the cycle phases was investigated by measuring the DNA content by flow-cytometry after cell staining with propidium iodide. Finally, real-time PCR explored the RNA expression levels of *p21.*

**Results:** All three melanoma cell lines showed a threefold decrease of their proliferation extent by Len at 10 μM (mean values: 640 ± 30 OD vs 1.915 ± 15 OD, p < 0.05). However, the majority of cells were viable (>91 %) although frozen at the G0–G1 phase of cell-cycle (64 ± 10 %) with respect to untreated cells (38 ± 4.6 %). Percentage number of apoptotic cells was apparently similar (Len-treated: 0.7 ± 0.02 vs untreated: 1.7 ± 0.04 %) as well as those undergoing to S and G2-M phases (Len-treated: 35 ± 3 vs untreated: 60 ± 2 %). After treatment with Len, melanoma cell were up-regulated in their *p21* RNA content (p < 0.01).

**Conclusions:** Len exerts a cytostatic effect on melanoma cell lines through *p21* up-regulation that restrains the NFkB activity. This provides the rationale for planning further pre-clinical investigations exploring the Len effect on both CDK4 and CDK6 expression and activity, in order to design combination strategies with negative regulators of the cell cycle for the treatment of melanoma.

References

1. Kotla V, Goel S, Nischal S, Heuck C, Vivek K, Das B, Verma A. Mechanism of action of lenalidomide in hematological malignancies. J Hematol Oncol. 2009;2:36.

2. Eisen T, Trefzer U, Hamilton A, Hersey P, Millward M, Knight RD, Jungnelius JU, Glaspy J. Results of a multicenter, randomized, double-blind phase 2/3 study of lenalidomide in the treatment of pretreated relapsed or refractory metastatic malignant melanoma. Cancer. 2010;116(1):146–54.

3. Mangiameli DP, Blansfield JA, Kachala S, Lorang D, Schafer PH, Muller GW, Stirling DI, Libutti SK. Combination therapy targeting the tumor microenvironment is effective in a model of human ocular melanoma. J Transl Med. 2007;5:38.

4. LeBlanc R, Hideshima T, Catley LP, Shringarpure R, Burger R, Mitsiades N, Mitsiades C, Cheema P, Chauhan D, Richardson PG, et al. Immunomodulatory drug costimulates T cells via the B7-CD28 pathway. Blood. 2004;103(5):1787–90.

#### Combination therapies

##### P12 Chemoimmunotherapy elicits polyfunctional anti-tumor CD8 + T cells depending on the activation of an AKT pathway sustained by ICOS

###### Ornella Franzese^1^, Belinda Palermo^2^, Cosmo Di Donna^2^, Isabella Sperduti^3^, MariaLaura Foddai^2^, Helena Stabile^4^, Angela Gismondi^4^, Angela Santoni^4^, Paola Nisticò^2^

####### ^1^Department of Systems Medicine, University of Tor Vergata, Rome, Italy; ^2^Department of Research, Advanced Diagnostics and Technological Innovation, Regina Elena National Cancer Institute, Rome, Italy; ^3^Biostatistics and Scientific Direction, Regina Elena National Cancer Institute, Rome, Italy; ^4^Department of Molecular Medicine, University of Rome “La Sapienza”, Rome, Italy

######## **Correspondence:** Paola Nisticò

*Journal of Translational Medicine* 2016, **14(Suppl 1)**:P12

**Background:** Harnessing the T-cell response has proven to be a successful strategy in the prevention of tumor relapse. Recently we reported that Melan-A-specific CD8 T cells isolated from long-term surviving patients treated with DTIC before peptide-vaccination plus IFN-α possess higher anti-tumor reactivity and an enlarged T-cell repertoire, compared to cells isolated after vaccination alone, suggesting that chemoimmunotherapy may favor a protective anti-tumor specific immune response.

**Materials and methods:** To identify the mechanisms enhancing the immune response, we analyzed the endogenous and treatment-induced antigen specific CD8+ T-cell response in a panel of Melan-A- and gp100-specific clones from five patients. We analyzed the differentiation phenotype (CCR7 and CD45RA), the co-stimulatory (CD27, CD28 and ICOS) and inhibitory (TIM-3, LAG-3 and PD-1) profile, in parallel with the polyfunctionality (IFN-γ, TNF-α and Granzyme B) and activation of AKT (pSer473-AKT).

**Results:** Melan-A-specific T cells isolated from the endogenous response of both treatments showed poor polyfunctionality and low ICOS expression and an AKT activation status related to the differentiation profile (in term of CD28 and/or CD27 expression). Conversely, after therapy only patients treated with combined chemoimmunotherapy showed tumor-specific CD8+ T cells displaying the hallmarks of differentiated and highly activated effector T cells. These clones were highly efficient in tumor killing, possessed polyfunctional activity, up-regulated inhibitory receptors, retained proliferative capability and activated an AKT pathway not-related to the differentiation phenotype and dependent on ICOS engagement. Strikingly, T-cell polyfunctionality elicited by the combined therapy was strictly dependent on this AKT activation, as demonstrated by the blockade with selective inhibitors, which occurred only in Melan-A-specific CD8+ T cells and not in cells specific for gp100, suggesting that the nature of the peptide is crucial for the activation of this pathway.

**Conclusions:** We suggest that the phenotypic and functional T-cell signature elicited by chemoimmunotherapy is a fine-tuned balance between quality of the Ag/TCR complex, co-stimulatory signals such as ICOS, inhibitory checkpoints and AKT activation, associated with anti-tumor T cells that can protect patients from tumor recurrence. The study represents a critical contribution for the comprehension of the mechanisms underlying the advantages of combined chemo-immunotherapy and paves the way for the identification of new biomarkers of T-cell activation that may be employed as markers of immune responsiveness.

##### P13 Favourable toxicity profile of combined BRAF and MEK inhibitors in metastatic melanoma patients

###### Andrea P. Sponghini^1^, Francesca Platini^1^, Elena Marra^2^, David Rondonotti^1^, Oscar Alabiso^1^, Maria T. Fierro^2^, Paola Savoia^2^, Florian Stratica^1^, Pietro Quaglino^2^

####### ^1^A.O.U. Maggiore della Carità, S.C. di Oncologia, Novara, Italy; ^2^A.O.U. Città della Salute e della Scienza, S.C. di Dermatologia, Torino, Italy

######## **Correspondence:** Andrea P. Sponghini

*Journal of Translational Medicine* 2016, **14(Suppl 1)**:P13

**Background:** Combination therapy with BRAF and MEK inhibitors is a recommended treatment strategy for metastatic melanoma patients with BRAFV600 mutations. Combining a selective BRAF inhibitor, such as dabrafenib, and a MEK inhibitor, such as trametinib, has been shown to improve the response rate and progression-free survival in patients with advanced melanoma. This combination treatment results in a reduction in skin toxicity seen with a BRAF inhibitor alone; however, addition of the MEK inhibitor adds other toxicities, such as pyrexia and gastrointestinal or ocular toxicity. Trametinib was approved as a single agent for the treatment of patients with V600E-mutated metastatic melanoma by FDA in May 2013 and by EMA in September 2013. Later FDA has approved the combination of dabrafenib and trametinib for the treatment of patients with BRAF V600E/K-mutant metastatic melanoma and their use seems to be currently the best approach.

**Materials and methods:** From November 2013 to September 2015, we have collected data from 54 patients with BRAF V600E/K mutation-positive stage IV melanoma. Patients received a combination of dabrafenib (150 mg orally twice daily) and trametinib (2 mg orally once daily). Metastatic sides were: skin (30 %), liver (28 %), lymph nodes (22 %), lung (15 %), brain (4 %) and spleen (1 %). Adverse events were monitored according to CTCAE v4.03. If dose modification was necessary we used standard dose reductions as recommended by manufacturers. Patients were monitored every 8 weeks and therapy has been continued until progression or unacceptable toxicity.

**Results:** Among our patients no malignant and hyperproliferative skin lesions, interstitial lung disease, venous thromboembolism, gastrointestinal bleeding and heart (cardiomyopathy, decreased left ventricular ejection fraction) toxicities were appreciated. Evolution of melanocytic nevi was also assessed, none of the monitored nevi increased in pigmentation nor morphological changes in preexisting nevi was seen following combination therapy. The most common adverse events (G1 grade) were fatigue (25 %) and pyrexia (23 %), no treatment discontinuation was necessary. Cutaneous rash (G1) occurred in 11 %, diarrhea (G1) and arthralgia (G1) in 5 %, ocular uveitis occurred in 2 % of 54 patients, but no dose modifications were necessary. Severe papilloedema occurred in 1 patient of 54, that led to permanent therapy discontinuation. Clinically meaningful improvements from baseline in favour of dabrafenib/trametinib were observed for pain, nausea and vomiting, distress and appetite at weeks 8 already.

**Conclusions:** The combination of dabrafenib and trametinib was well tolerated and patients reported a better global health and functional status with less exacerbation of symptoms.

##### P14 Electrothermal bipolar vessel sealing system dissection reduces seroma output or time to drain removal following axillary and ilio-inguinal node dissection in melanoma patients: a pilot study

###### Di Monta Gianluca, Caracò Corrado, Di Marzo Massimiliano, Marone Ugo, Di Cecilia Maria Luisa, Mozzillo Nicola

####### Department Melanoma, Div Surgery of Melanoma, National Cancer Institute “Fondazione Pascale”, Naples, Italy

######## **Correspondence:** Di Monta Gianluca

*Journal of Translational Medicine* 2016, **14(Suppl 1)**:P14

**Background:** Radical lymph node dissection (RLND) is the treatment of choice in stage III melanoma patients. Over half of the observed complications were related to post-operative serum collection affecting wound healing. The possibility to reduce seroma formation and the drainage permanence may offer benefits to the patients. The aim of the study was to evaluate the impact of this technique to decrease post-operative lymph output and time of drainage removal. This represents the first report of electrothermal bipolar vessel sealing system (SJ) in melanoma patients affected by nodal disease submitted to nodal dissection.

**Methods:** This was a prospective study carried out at a single center in which consecutive patients were submitted to radical axillary or ilio-obturator dissection using SJ. Control group data of seroma output and drainage removal were obtained from a recent Author’s analyses published in 2012 in which were analysed patients submitted to nodal dissection with conventional electrocautery. Daily drainage volume was compared with historical control group on days 3, 10 or 15 and day 20. Duration of drainage was compared with historical control group.

**Results:** 70 patients with melanoma underwent an axillary or ilio-inguinal RLND and were included in the study. The mean postoperative lymph output was lower at days 3, 10, 15 and 20 than historical control group. About 66.6 % of patients had less of 55 cc of drainage at 10 days. In patients treated with SJ the mean of drainage removal was lower than historical control group and was 14.5 and 24.0 days respectively.

**Conclusion:** This is the first report of nodal dissection in patients with cutaneous melanoma performed with electrothermal bipolar vessel sealing system, the technique was feasible, safe, and effective. Compared with historical data regarding the conventional electrocautery, this technique seems to result in reduced drainage volume and duration of days of suction drain. Prospective randomized controlled studies are necessary to evaluate the effect of this technique on perioperative complications.

#### News in immunotherapy

##### P15 Clinical and immunological response to ipilimumab in a metastatic melanoma patient with HIV infection

###### Francesco Sabbatino, Celeste Fusciello1, Antonio Marra, Rosario Guarrasi, Carlo Baldi, Rosa Russo, Di Giulio Giovanni, Vincenzo Faiola, Pio Zeppa, Stefano Pepe

####### Department of Onco-Hematology, AOU S. Giovanni di Dio e Ruggi D’Aragona, University of Salerno, Salerno, Italy

######## **Correspondence:** Francesco Sabbatino

*Journal of Translational Medicine* 2016, **14(Suppl 1)**:P15

**Background:** Immunotherapy with immune-checkpoint molecule inhibitors such as anti-CTLA4, anti-PD-1 and anti-PD-L1 increases the overall survival of melanoma patients with or without BRAF V600 mutations. This effect is associated with an enhanced antitumor T cell response. Patients with HIV infection show a high incidence of primary melanoma because of their immunodeficient status. No effective treatment options are currently available for patients with HIV and metastatic melanoma and few data are available in literature for those treated with immune checkpoint-molecule inhibitors. Evidence suggests that both CTLA4 and PD-1 may play a role in HIV viral persistence as well as in the production of HIV-specific CD4 T cells. Here we report our recent experience in a patient with metastatic melanoma and HIV infection treated with ipilimumab in combination with antiretroviral therapy.

**Materials and methods:** A 49-year-old man was diagnosed with HIV AIDS in 2011. After treatment with multiple antiretroviral therapies, he was treated with the combination of tenofovir, emtricitabine and efavirence. In 2015 he was diagnosed with melanoma with multiple lymph node and lung metastases. Furthermore his history showed the diagnosis of a previous basal cell carcinoma of the back and the presence of severe anal dysplasia HPV-related. BRAF status was detected using the **“**Cobas” test. Ipilimumab was administered at 3 mg/kg intravenously, every 3 weeks, for four doses, starting from June 7 to August 8, 2015. Restaging TC scan was performed on September 9, 2015. Total number of CD3+/CD4+/CD8+/CD16+/CD19+ cells and levels of HIV RNA copies were monitored during the treatment.

**Results:** A BRAF V600E mutation was found. No adverse events were reported. No significant changes were found in the number of CD3+/CD8+/CD16+/CD19+ cells. At baseline, the number of CD4+ cells was 296 cells/μl while the HIV RNA copies were 20/ml. An increase in the number of CD4+ cells and a decrease in HIV RNA copies were already detected after the first administration of ipilimumab. Following four courses of ipilimumab the number of CD4+ cells was 580 cells/μl, while the HIV RNA copies were not detectable. Restaging TC scan showed disappearance of previous lesions and no development of new lesions.

**Conclusions:** Our single-patient experience demonstrates that administration of ipilimumab in HIV infected metastatic melanoma patient is safe and well tolerated. Furthermore it demonstrated to have a beneficial effects on both HIV infection and melanoma treatment. Clinical trials are warranted to determine the clinical efficacy of ipilimumb in this setting.

##### P16 Immunotherapy and hypophysitis: a case report

###### Elisabetta Gambale, Consiglia Carella, Alessandra Di Paolo, Michele De Tursi

####### Department of Medical, Oral and Biotechnological Sciences, University G. D’Annunzio of Chieti-Pescara, Chieti, Italy

######## **Correspondence:** Elisabetta Gambale

*Journal of Translational Medicine* 2016, **14(Suppl 1)**:P16

**Background:** Recent studies suggest that hypophysitis occurs in about 10–15 % of patients receiving Ipilimumab [1, 2], in contrast with the rarity of idiopathic autoimmune hypophysitis or hypophysitis after treatment with other immunotherapies [1, 3–8]. Here we present the case of a patient with Ipilimumab- induced hypophysitis (IH).

**Case report:** A 68-year-old man was admitted to our Oncology Unit in June 2015 with the diagnosis of metastatic melanoma. ECOG Performance Status was 0 and no comorbidities were reported. In June 2011 primary melanoma was removed surgically; during follow up, in May 2015, bilateral lung metastases were highlighted by CT-scan. Pathological lymph nodes in left axilla (more than 8 cm in diameter) were also reported. Ipilimumab-based therapy started in June 2015. In August 2015, after the third course out four scheduled doses of Ipilimumab, the patient referred us one-week history of heavy headache. No nausea, vomiting, visual symptoms, polydipsia or polyuria were reported. A brain MRI was performed and a pituitary swelling consistent with hypophysitis was diagnosed. Haematological reports were consistent with hypopituitarism diagnosis: morning cortisol level 0,7 mcgr/dl (reference range 8–25) and TSH level 0,208 UI/ml (0,250–4,5). Therapy with dexamethasone 4 mg IM every 6 h was started; due to the low decreasing THS values, levothyroxine 100 mcg once daily was also added. A rapid symptoms improvement was obtained by this therapeutic approach. The dose of dexamethasone was gradually decreased over 4 weeks and hydrocortisone replacement of 20 mg in twice daily doses has been just started. The fourth course of Ipilimumab was not performed because of the contemporary high-dose glucocorticoid therapy; furthermore, it occurred delayed liver toxicity: AST 160 U/L (15–46) and ALT 350 U/L (11–66). Total body CT-scan and brain MRI were scheduled, but a significant shrinkage in axillary lymph node swelling by clinical examination was observed.

**Discussion:** Development of IH is associated with a significant clinical morbidity, so early recognition and therapeutic intervention are extremely relevant. Furthermore, in the absence of data demonstrating clinical benefit from high-dose glucocorticoid therapy, we wondered if, in absence of symptoms such as visual disturbances, there is the indication to start with hydrocortisone replacement therapy alone from the beginning [1]. We also wondered if it was reasonable to administer Ipilimumab during prolonged high- dose glucocorticoid therapy. Thus, guidelines for surveillance and management of IH are highly required [9, 10].

**Consent to publish** Written informed consent for publication of their clinical details was obtained from the patient/parent/guardian of the patient. A copy of the consent form is available for review by the Editor of this journal.

References

1. Faje A. Immunotherapy and hypophysitis: clinical presentation, treatment and biologic insights. Pituitary. 2015. (**Epub ahead of print**).

2. Albarel F et al. Long- term follow- up of Ipilimumab-induced Hypophysitis, a common adverse event of the anti- CTLA-4 antibody in melanoma. Eur J Endocrinol. 2015;172:195–204.

3. Torino F, Bernabei A, et al. Thyroid dysfunction as an unintended side effect of anticancer drugs. Thyroid. 23:1345–66.

4. Chan WB et al. Panhypopituitarism in association with interferon- alpha treatment. Singap Med J. 2004;45:93–94.

5. Concha LB et al. Interferon- induced hypopituitarism. Am J Med. 2003;114:161–3.

6. Ridruejo et al. Central hypothyroidism and hypophysitis during treatment of chronic hepatitis C with pegylated interferon alpha and ribavirin. Eur J Gastroenterol Hepatol. 2006;18:693–94.

7. Sakane N et al. Reversible hypopituitarism after interferon- alpha therapy. Lancet. 1995;345:305.

8. Tebben et al. Granulomatous adenohypophysitis after interferon and ribavirin therapy. Endoc Pract. 2007;13:169–175.

9. Juszczak A et al. Ipilimumab: a novel immunomodulating therapy causing autoimmune hypophysitis: a case report and review. Eur J of Endocrinology. 2012;167:1–5.

10. Lam T et al. Ipilimumab- induced hypophysitis in melanoma patients: An Australian case series. Intern Med J. 2015. (**Epub ahead of print**).

#### Tumor microenvironment and biomarkers

##### P17 New immuno- histochemical markers for the differential diagnosis of atypical melanocytic lesions with uncertain malignant potential

###### Laura Marra^*^, Giosuè Scognamiglio, Monica Cantile, Margherita Cerrone, Fara De Murtas, Valeria Sorrentino, Anna Maria Anniciello, Gerardo Botti

####### Pathology Unit, Istituto Nazionale Tumori Fondazione “G. Pascale”, Napoli, Italy

######## **Correspondence:** Laura Marra

*Journal of Translational Medicine* 2016, **14(Suppl 1)**:P17

**Background:** For specific subsets of melanocytic proliferations, there are morphologic limitations in the histologic diagnosis, especially for borderline melanocytic tumors. [1]. In particular, Spitzoid proliferations can be difficult to diagnose. For these reasons, in the last years, clinic research has focused attention on discovery of new diagnostic markers.

Published gene expression and proteomic profiling data indicate new candidate molecules involved in melanoma pathogenesis, and useful in differential diagnosis of difficult melanocytic lesions [2,3].

Recently, the diagnostic power of galectin-3 was demonstrated in a series of melanocytic lesions, with a strong increasing of expression in malignant lesions compared with benign lesions. Similarly, the accumulation of Collagen XVII antibody was detected in vertical melanoma fronts and associated with invasive phenotype. Moreover, overexpression of cyclin D1 and p21 was detected in Spitz nevi compared with non-spitzoid melanomas, Ki-67 appears highly expressed in deep areas of non-spitzoid melanomas [4,5]

The purpose of this study was to evaluate the expression of a panel of markers, suggested by literature, on a series of melanocytic lesions selected from our archives, to define the most useful for the differential diagnosis.

**Materials and methods:** We selected 56 samples from database of Pathology Unit of I.N.T. “G. Pascale” (Naples): 24 atypical Spitz tumor, 10 atypical Spitz nevi, 12 primary melanomas, 5 blue nevi and 5 common nevi. The samples were analyzed by immunohistochemistry for the expression of Cyclin D1, Ki67, Galectin 3, COX2, Topoisomerase 2α, Collagen XVII, GLUT1, Survivin. Ten atypical lesions and ten melanomas were also used for a molecular analysis to evaluate specific chromosomal aberrations, typical of melanoma, by FISH.

**Results:** Almost all of the atypical lesions showed a low proliferative index (<5 %), while the high expression of Ki67 is present in malignant lesions. The expression of cyclin D1, collagen XVII and Topoisomerase 2α was high in borderline lesions and in melanomas with a Breslow thickness >1 mm and high mitotic index, and lower in common and blue nevi. The expression of COX2, survivin is not particularly significant, reflecting the trend described in the literature. Only three atypical lesions showed alteration in the RREB1 locus.

**Conclusions:** Our preliminary data showed that Cyclin D1, the collagen XVII and Topoisomerase 2α could provide useful diagnostic and prognostic markers for atypical lesions, being significantly overexpressed in invasive lesions.

References

1. Magro CM, Crowson AN, Mihm MC Jr, Gupta K, Walker MJ, Solomon G.The dermal-based borderline melanocytic tumor: a categorical approach. J Am Acad Dermatol. 2010;62(3):469–79.

2. Busam KJ, Fang Y, Jhanwar SC, Pulitzer MP, Marr B, Abramson DH. Distinction of conjunctival melanocytic nevi from melanomas by fluorescence in situ hybridization. J CutanPathol. 2010;37(2):196–203.

3. DeMarchis EH, Swetter SM, Jennings CD, Kim J. Fluorescence in situ hybridization analysis of atypical melanocytic proliferations and melanoma in young patients. Pediatr Dermatol. 2014;31(5):561–9.

4. Szekeres G, Battyáni Z. Immuno-diagnosis of malignant melanoma. MagyOnkol. 2003;47(1):45–50.

5. McCormack CJ, Conyers RK, Scolyer RA, Kirkwood J, Speakman D, Wong N, Kelly JW, Henderson MA. Atypical Spitzoid neoplasms: a review of potential markers of biological behavior including sentinel node biopsy. Melanoma Res. 2014;24(5):437–47.

##### P18 Utility of simultaneous measurement of three serum tumor markers in melanoma patients

###### Angela Sandru^1^, Silviu Voinea^1^, Eugenia Panaitescu^2^, Madalina Bolovan^3^, Adina Stanciu^3^, Sabin Cinca^3^

####### ^1^Department of Surgical Oncology, “Carol Davila” University of Medicine and Pharmacy; “Alexandru Trestioreanu” Oncologic Institute, Bucharest, Romania; ^2^Department of Medical Informatics and Biostatistics,“Carol Davila” University of Medicine and Pharmacy, Bucharest, Romania; ^3^Department of Carcinogenesis and Molecular Biology, “Alexandru Trestioreanu” Oncologic Institute, Bucharest, Romania

######## **Correspondence:** Angela Sandru

*Journal of Translational Medicine* 2016, **14(Suppl 1)**:P18

**Background:** Unlike other malignancies, in which measurement of serum tumor markers is routine, in malignant melanoma their use has remained far behind. The only generally accepted serological marker for melanoma is Lactatdehydrogenase (LDH) [1]. This study aims to compare the ability of three serum markers, Melanoma Inhibitory Activity (MIA), LDH and C Reactive Protein (CRP), to identify patients with melanoma. MIA serum concentration represents an independent predictor for melanoma patients’ survival [2], while an increase in serum CRP is considered a sign of melanoma progression [3].

**Materials and methods:** During January 2014–May 2015 we determined MIA, LDH and CRP serum concentrations in 100 melanoma patients (22-stage I, 42-stage II, 25-stage III, 13-stage IV) and 51 healthy donors. The proteins were measured by a high sensitivity ELISA method. For CRP and LDH, were accepted the thresholds recommended by the kit manufacturers: 5 mg/L, respectively 225 U/L. Using the ROC curve, MIA cut-off level was estimated at 12.5 ng/mL, value at which the marker has the maximum capacity to discriminate between healthy and melanoma subjects.

**Results:** MIA sensitivity is greater than that of CRP or LDH for stage IV (76.9 %/69.2 %/53.8 %). Conversely CRP is more sensitive than MIA or LDH in identifying patients with localized disease (17.74 %/8.06 %/3.23 %). Taking into account the whole lot, combination of CRP and MIA identifies more patients than that formed by MIA and LDH or LDH and CRP (40.0 %/32.0 %/30.0 %). Interestingly, none of the 62 patients with localized disease had more than one marker above the admitted cut-off, while in stage IV at least one of the markers was increased in all individuals. Patients with a serum MIA > 12.5 ng/mL have a likelihood of disseminated disease (stage III and IV), 12 times higher than those with MIA < 12.5 ng/mL (OR = 12.73; 95 %CI = 3.3810−47.9148; p = 0.000169), while a pathological LDH increases the probability of disseminated disease only 10 times (OR = 10.71;95 % CI = 1.7527−65.3953; p = 0.010235).

**Conclusion:** Simultaneous measurement of LDH, CRP and MIA identifies a larger number of patients than measurement of any marker alone or any possible combination of two of them. But adding LDH to MIA/CRP association leads to an increase in sensitivity with only 4 %. Although serum LDH is used in TNM staging system, we noted that, even in stage IV, it has a lower sensitivity than either of the other two studied markers. Regarding stage IV patients, MIA/CRP combination has 100 % sensitivity, making LDH determination useless. We believe that simultaneous measurement of MIA/CRP best describes melanoma patients.

**Source of funding** Project PN-II-PCCA type 1 Nr.4/2012 (HRCarrays) “Array structures for prevention, individualized diagnosis and treatment in cancers with high risk of incidence and mortality”

References

1.Balch CM, Gershenwald JE, Soong SJ, Thompson JF, Atkins MB, Byrd DR, Buzaid AC, et al. Final version of 2009 AJCC melanoma staging and classification. J Clin Oncol. 2009;27(36):6199–206.

2. Sandru A, Panaitescu E, Voinea S, Bolovan M, Stanciu A, Cinca S, Blidaru A. Prognostic Value of Melanoma Inhibitory Activity Protein in Localized Cutaneous Malignant Melanoma. J Skin Cancer. 2014; Article ID 843214.

3. Fang S, Wang Y, Sui D, Liu H, Ross MI, Gershenwald JE, et al. C-reactive protein as a marker of melanoma progression. J Clin Oncol. 2015;20 (33):1389–96.

##### P19 The significance of various cut-off levels of melanoma inhibitory activity in evaluation of cutaneous melanoma patients

###### Angela Sandru^1^, Silviu Voinea^1^, Eugenia Panaitescu^2^, Madalina Bolovan^3^, Adina Stanciu^3^, Sabin Cinca^3^

####### ^1^Department of Surgical Oncology, “Carol Davila” University of Medicine and Pharmacy; “Alexandru Trestioreanu” Oncologic Institute, Bucharest, Romania; ^2^Department of Medical Informatics and Biostatistics,“Carol Davila” University of Medicine and Pharmacy, Bucharest, Romania; ^3^Department of Carcinogenesis and Molecular Biology, “Alexandru Trestioreanu” Oncologic Institute, Bucharest, Romania

######## **Correspondence:** Angela Sandru

*Journal of Translational Medicine* 2016, **14(Suppl 1)**:P19

**Background:** Cutaneous melanoma is a tumor with intense metabolic activity that expresses and releases into blood circulation a large number of enzymes, cytokines and growth factors. In this study we aimed to identify optimal cut-off of serum melanoma inhibitory activity (MIA), a protein synthesized by malignant melanocytes. As there is no maximum MIA serum concentration widely accepted as the upper normal limit, every laboratory calculates its own cut-off value. We have established in a previous study a MIA threshold of 9.4 ng/mL [1]. Various MIA cut-off values were reported in literature, ranging from 6.5 [2] to 17.4 ng/mL [3].

**Materials and methods:** During the past year, MIA serum concentration was measured by a high sensitivity ELISA method in 100 melanoma patients staged as follows: stage I-22, stage II-42, stage III-25 and stage IV-13. Also, blood was collected from 51 healthy controls. In order to evaluate MIA ability to differentiate melanoma patients from healthy subjects, we plotted the ROC curve for the entire group and for each stage separately and estimated area under the curve (AUC).

**Results:** In the control group the lowest MIA value was 5.08 ng/mL and the highest 16.35 ng/mL. In melanoma patients group, MIA serum concentration ranged between 4.39 and 121.29 ng/mL with a mean of 12.21 ng/mL and a median of 8.07 ng/mL. We have noticed a significant increase in MIA serum concentration in stage IV compared with all the other stages (p < 0.000061 Likelihood Ratio). Taking into account the entire lot, the optimal ratio between sensitivity and specificity was found for a MIA value of 9.125 ng/mL, same as for localized disease, but for these two categories the ability of MIA to separate melanoma subjects from healthy ones is very low, making it useless (Sn = 35.0 %, respectively 19.4 %). For patients with metastases the optimal cut-off was set at 12.46 ng/mL (AUC = 0.910; p < 0.0001; Sn = 84.60 %; Sp = 86.30 %). To facilitate application in clinical practice, the above values were approximated to 9, 9.5 and 12.5. By raising the threshold from 9 ng/mL to 12.5 ng/mL, MIA diagnostic efficacy for melanoma was increased from 33.77–43.71 %.

**Conclusions:** In our study a MIA cut-off of 12.5 ng/mL provided maximum diagnostic efficacy for each clinical stage compared to a cut-off of 9 or 9.5 ng/mL. Likelihood of having melanoma for an individual with MIA serum concentration greater than 12.5 ng/mL was 75 %. At this cut-off, MIA ability to differentiate between patients with metastases and healthy people was 84 %.

**Source of funding** Project PN-II-PCCA type 1 Nr.4/2012 (HRCarrays) “Array structures for prevention, individualized diagnosis and treatment in cancers with high risk of incidence and mortality”

References

1. Sandru A, Panaitescu E, Voinea S, Bolovan M, Stanciu A, Cinca S, Blidaru A. Prognostic Value of Melanoma Inhibitory Activity Protein in Localized Cutaneous Malignant Melanoma. J Skin Cancer. 2014; Article ID 843214.

2. Bosserhoff AK, Kaufmann M, Kaluza B, Bartke I, Zirngibl H, Hein R, Stolz W, Buettner R. Melanoma-inhibiting activity, a novel serum marker for progression of malignant melanoma, Cancer Res. 1997 Aug 1;57(15):3149–53.

3. Tas F, Yasasever V, Duranyildiz D, Camlica H, Ustuner Z, Aydiner A, Topuz E. Clinical value of protein S100 and melanoma-inhibitory activity (MIA) in malignant melanoma, Am J Clin Oncol. 2004 Jun;27(3):225–8.

##### P20 The long noncoding RNA *HOTAIR* is associated to metastatic progression of melanoma and it can be identified in the blood of patients with advanced disease

###### Chiara Botti^1^, Giosuè Scognamiglio^2^, Laura Marra^2^, Gabriella Aquino^2^, Rosaria Falcone^1^, Annamaria Anniciello^2^, Paolo Antonio Ascierto^1^, Gerardo Botti^2^, Monica Cantile^2^

####### ^1^Unit of Melanoma, Cancer Immunotherapy and Innovative Therapy, Istituto Nazionale Tumori Fondazione “G. Pascale”, Napoli, Italy; ^2^Pathology Unit, Istituto Nazionale Tumori Fondazione “G. Pascale”, Napoli, Italy

######## **Correspondence:** Monica Cantile

*Journal of Translational Medicine* 2016, **14(Suppl 1)**:P20

**Background:** The molecular mechanisms responsible for the metastatic progression of melanoma have not been fully defined yet [1, 2]. We have recently shown that an important role in this process is certainly played by HOX genes [2]. Regulation of HOX genes expression is under control of particular non-coding RNAs, some of which are present within the HOX locus [3]. HOTAIR is the most studied among them, whose aberrant expression is associated with the metastatic progression of many malignancies [4].

The main objective of this study was to verify the role played by HOTAIR in metastatic progression of melanoma and to evaluate the circulating levels of HOTAIR in the blood of patients with metastatic melanoma.

**Materials and methods:** A series of benign melanocytic/borderline lesions, primary melanomas pT1a/b, primary melanomas pT3/pT4, associated with the corresponding metastases and visceral metastases, were selected to evaluate the potential changes in the expression of HOTAIR during the evolution of the disease. HOTAIR gene expression studies were carried out both by ISH and RT-PCR, to quantitate the expression levels of the lncRNA in tissues and in the serum of metastatic patients.

**Results:** None of the benign melanocytic lesions showed the presence of HOTAIR. The staining of HOTAIR resulted very weak in the primary pT1 lesions, while it was very strong in all pairs of primary tissues and corresponding metastases, with a cytoplasmic and membrane positivity. Surprisingly, we found the presence of HOTAIR in some intratumoral lymphocytes, while this positivity decreased in lymphocyte component further away from the tumor. HOTAIR gene expression data were similar to those obtained by ISH. Finally, HOTAIR was detected in the serum of all analyzed metastatic patients.

**Conclusions:** These data allowed us to speculate on the fundamental role played by HOTAIR in the tumor evolution of melanoma. In addition, its presence in intratumoral lymphocytes could suggest that it may be also involved in the modulation of the tumor microenvironment [5]. Finally, its detection in serum might suggests its use, as circulating serum marker, in the management of melanoma patients.

References

1. Botti G, Scognamiglio G, Marra L, Collina F, Di Bonito M, Cerrone M, Grilli B, Anniciello A, Franco R, Fulciniti F, Ascierto PA, Cantile M. SPARC/osteonectin is involved in metastatic process to the lung during melanoma progression. Virchows Arch. 2014;465(3):331–8.

2. Cantile M, Scognamiglio G, Anniciello A, Farina M, Gentilcore G, Santonastaso C, Fulciniti F, Cillo C, Franco R, Ascierto PA, Botti G. Increased HOX C13 expression in metastatic melanoma progression. J Transl Med. 2012;10:91.

3. Soshnikova N. Hox genes regulation in vertebrates. Dev Dyn. 2014;243(1):49–58.

4. Wu Y, Zhang L, Wang Y, Li H, Ren X, Wei F, Yu W, Wang X, Zhang L, Yu J, Hao X. Long noncoding RNA HOTAIR involvement in cancer. Tumour Biol. 2014;35(10):9531–8.

5. Botti G, Cerrone M, Scognamiglio G, Anniciello A, Ascierto PA, Cantile M. Microenvironment and tumor progression of melanoma: new therapeutic prospectives. J Immunotoxicol. 2013;10(3):235–52.

#### Other

##### P21 The effect of Sentinel Lymph Node Biopsy in melanoma mortality: timing of dissection

###### Cristina Fortes^1^, Simona Mastroeni^1^, Alessio Caggiati^2^, Francesca Passarelli^3^, Alba Zappalà^4^, Maria Capuano^5^, Riccardo Bono^6^, Maurizio Nudo^6^, Claudia Marino^7^, Paola Michelozzi^7^

####### ^1^Epidemiology Unit, IDI-IRCCS, Rome, Italy; ^2^Plastic Surgery Unit, IDI-IRCCS, Rome, Italy; ^3^Pathology Unit IDI-IRCCS, Rome, Italy; ^4^Oncology Unit IDI-IRCCS, Rome, Italy; ^5^Division Dermatology Villa Paola, Capranica, Italy; ^6^V Division Dermatology IDI-IRCCS, Rome, Italy; ^7^Department of Epidemiology of the Regional Health Service, Rome, Italy

######## **Correspondence:** Cristina Fortes

*Journal of Translational Medicine* 2016, **14(Suppl 1)**:P21

**Background:** Although SLNB is considered an useful instrument for staging, to guide treatment decisions and as entry criteria for clinical trials [1], some controversies remain in terms of its therapeutic benefit [2].

**Materials and methods:** The aim of the study was to evaluate the effect of early sentinel lymph node biopsy (SLNB) in patients with primary melanoma (≤30 days) versus delayed SLNB (≥31 days), controlling for all known histological prognostic parameters.We conducted a 10-year cohort study among 748 patients who had undergone excision of the primary melanoma plus sentinel-node biopsy between January 1998 and December 2008 with a Breslow thickness of 1.00 mm or more and from the same geographic area (Lazio). Survival probability was estimated by Kaplan–Meier method, and prognostic factors were evaluated by multivariate analysis (Cox proportional hazards model).

**Results:** Out of the 748 patients with cutaneous melanoma, 141 patients were SLNB positive (18.9 %) and 607 cases (81.1 %) were negative. Patients with positive SLNB had a higher frequency of melanoma with more than 2 mm of Breslow thickness (66.7 versus 31.8 %, p < 0.0001), with the presence of ulceration (26.2 versus 15.3 %, p = 0.002), high mitotic rate (61.2 versus 32.3 %, p < 0.0001) and lower frequency of marked tumor-infiltration (8.7 versus 23.9 %, p = 0.03) than patients with negative SLNB. The frequency of nodular melanomas was also higher among patients with positive SLNB in comparison to negative SLNB (36.4 versus 21.0 %, p < 0.0001). No difference was found for sex, age, anatomic site, timing of SLNB by SLNB status. Ten-year melanoma specific survival was 78.7 %. Because a statistical interaction was observed between timing of SLNB and SLNB status (p = 0.002), the model was run for SLNB positive patients and SLNB negative patients. Among patients with positive node and who had undergone early dissection (≤30 days) of SLNB, 10-year melanoma survival was 76.5 versus 39.9 % among those who had undergone delayed SLNB dissection (≥31 days). After adjusting for sex, age, Breslow thickness, mitotic rate, ulceration and histological type, patients with a positive SLNB and who underwent early SLNB (≤30 days) had a three times decreased risk of melanoma mortality (HR: 0.29; 95 % CI: 0.11–0.77) in comparison with patients with positive SLNB who were underwent delayed SLNB (≥31 days). No protective effect was found for early dissection among negative node (HR:1.77; 95 % CI:0.97–3.26).

**Conclusion**s: These data suggest that, among melanoma patients with positive SLNB, early dissection of SLNB (≤30 days) decreased melanoma mortality.

References

1. Balch CM, Gershenwald JE, Soong SJ, Thompson JF, Atkins MB, Byrd DR, et al. Final version of 2009 AJCC melanoma staging and classification. J Clin Oncol. 2009;27(36):6199–206.

2. Yang JC, Sherry RM, Rosenberg SA. Melanoma: Why is sentinel lymph node biopsy ‘standard of care’ for melanoma? Nat Rev Clin Oncol. 2014;11(5):245–46.

##### P22 Epidemiological survey on related psychopathology in melanoma

###### Valeria De Biasio^1^, Vincenzo C. Battarra^2^

####### ^1^Mental Health Department, Hospital of Lauria, Italy; ^2^Dermatology and Oncology surgery of skin growths Department, Hospital of Caserta, Italy

######## **Correspondence:** Vincenzo C. Battarra

*Journal of Translational Medicine* 2016, **14(Suppl 1)**:P22

**Background:** 30 % of melanoma patients present high levels of psychological stress and would require clinical intervention [1–2]

This study evaluates whether patients with melanoma experience:

1) anxiety, depression and distress;

2) depression, anxiety, distress and psychological distress correlated with each other;

3) socio-demographic factors predisposed to related psychopathology;

4) the coping strategies most frequently used to influence the anxiety, depression and distress experienced.

**Materials and methods:** To manage this study we contacted 107 patients referred to the clinic of Dermatology of the Hospital “Sant’Anna e San Sebastiano” in Caserta. Fifty-five of them accepted to be interviewed.

The sample was composed of 29 females (47.3 %) and 26 males (52.7 %) with a mean age of 52.51 (52.65 male, 52.38 female).

Patients were required to fill a socio-demographic and clinical form inquiring about sex, marital status, education, occupation and date of diagnosis.

Patients were administered HADS, distress thermometer, BSI and COPE NVI.

**Results:** Women have higher scores in all scales with psychological distress as the more significant statistically value (p = 0.014).

About marital status and its correlation with the values of the scales under consideration, statistically significant results are observed for unmarried patients with BSI values much higher than married patients (p = 0.034) and with anxiety symptoms to HADS (p = 0.001). The comparison between the means shows that a low level of education is significantly associated with depression symptoms HADS (p = 0.016) and the total score (p = 0.036). Relating the values of the scales to each other, we appreciated that HADS and thermometer distress correlated positively (r = 0.329) whether for anxiety symptoms (p = 0.032; r = 0.289) or for depression (p = 0.037; r = 0.282), and BSI values correlate significantly with all other scales.

Among the risk factors, such as smoking, alcohol, lack of exercise or inappropriate diet, those who take alcohol and live sedentarily present higher levels of depression (p = 0.029 and p = 0.013).

Coping Strategies (COPE-NVI): social support correlates positively with distress (r = 0.337), avoidance correlates positively with depression (r = 0.357), positive attitude negatively correlates with depressive symptoms (r = −0.362), problem orientation correlates negatively with depressive symptoms (r = −0.484), and transcendent orientation positively correlates with depressive symptoms (r = 0.337).

**Conclusions:** Melanoma and psychopathological aspects correlate positively with each other, especially in relation to gender, marital status, education, risk factors and coping styles.

References

1. Sollner W, Zingg-Schir M, Rumpold G, Mairinger G, Fritsch P. Need for supportive counselling —The professionals’ versus the patients’ perspectives. A survey in a representative sample of 236 melanoma patients. Psychotherapy and Psychosomatics. 1998;67:94–104

2. Nadine A. Kasparian, Jordana K. McLoone, Phyllis N Butow. Psychological Responses and Coping Strategies Among Patients With Malignant Melanoma: a systematic review of the literature.

## IMMUNOTHERAPY BRIDGE

### KEYNOTE SPEAKER PRESENTATIONS

#### Immunotherapy beyond melanoma

##### K19 Predictor of response to radiation and immunotherapy

###### Silvia Formenti

####### Department of Radiation Oncology, Weill Cornell Medical College, New York, USA

######## **Correspondence:** Silvia Formenti

*Journal of Translational Medicine* 2016, **14(Suppl 1)**:K19

Radiation therapy contributes both immunogenic and immunosuppressive signals to the tumor microenvironment. Preclinical strategies to enhance the formers and/or mitigate the latter have demonstrated the concrete possibility to shift this balancing act toward a therapeutic success (1). Results from preclinical experiments, in immunocompetent syngeneic models mimicking the setting of advanced cancer treated by radiation and immunotherapy have consistently found clinical confirmation. Particularly when combined with immune checkpoint blockade, radiotherapy has demonstrated to be a powerful adjuvant to immunotherapy. Clinical examples of synergy between radiation and immune checkpoint inhibitors have been reported, and interim results in our prospective clinical trial confirm this finding (2–7).

Currently, multiple clinical trials are exploring optimal combinations and scheduling of radiotherapy and immunotherapy. While at least some early evidence from these trials confirms the hypothesis that radiation can enhances responses to immune checkpoint inhibitors, in the majority of patients tumors remain unresponsive, warranting research to identify markers that predict response. A recent study testing radiation with ipilimumab in melanoma (8) suggested that tumor expression of PDL-1 may predict lack of response to radiation and ipilimumab. However, in lung cancer patients treated with radiation and ipilimumab we found high PDL-1 expression among patients achieving durable complete and partial responses, without addition of PD-1 pathway inhibitors (9). In fact, higher expression of immune checkpoints has been hypothesized as a marker of more immunogenic tumors (10). In addition, pre-treatment mutational load has been found to be associated with responses to immune checkpoint inhibitors (11). It will be important to determine if radiation can compensate tumors with a low mutational load, by inducing induce de novo T cell priming to multiple tumor antigens (12) and could, therefore, achieve responses in the absence of pre-existing neoantigens (13)

Importantly, the overall degree of immune impairment of the patients may be a critical predictor of response to radiation + immunotherapy. For instance, we found the pretreatment neutrophil/lymphocyte ration might enable a priori selection of individuals with a propensity to respond to the combination of radiation and GM-CSF (14). In another study in metastatic breast cancer patients we found that the impaired ability of T cells to signal in response to TCR stimulation was associated with a shorter progression-free survival after treatment with radiation and fresolimumab (Formenti et al., in preparation).

Overall, while radiation has emerged as a promising partner for immunotherapy, the identification of tumor and patient characteristics that can predict which patients should receive upfront the combination of immunotherapy with radiotherapy instead of immunotherapy alone remains unclear.

References

1. Formenti SC, Demaria S. Combining radiotherapy and cancer immunotherapy: a paradigm shift. J Natl Cancer Inst. 2013;105(4):256–65.

2. Demaria S, Kawashima N, Yang AM, et al. Immune-mediated inhibition of metastases following treatment with local radiation and CTLA-4 blockade in a mouse model of breast cancer. Clin Cancer Res. 2005;11:728–34.

3. Postow MA, Callahan MK, Barker CA, et al. Immunologic correlates of the abscopal effect in a patient with melanoma. N Engl J Med. 2012;366(10):925–31.

4. Hiniker SM, Chen DS, Reddy S, et al. A systemic complete response of metastatic melanoma to local radiation and immunotherapy. Transl Oncol. 2012;5(6):404–07.

5. Stamell EF, Wolchok JD, Gnjatic S, Lee NY, Brownell I. The abscopal effect associated with a systemic anti-melanoma immune response. Int J Radiat Oncol Biol Phys. 2013;85(2):293–95.

6. Golden EB, Demaria S, Schiff PB, Chachoua A, Formenti SF. An abscopal response to radiation and ipilimumab in a patient with metastatic non-small cell lung cancer. Cancer Immunol Res. 2013;1(6):365–72.

7. Demaria S, Golden EB, Formenti SC. Role of Local Radiation Therapy in Cancer Immunotherapy. JAMA Oncol. 2015.

8. Twyman-Saint Victor C, Rech AJ, Maity A, Rengan R, Pauken KE, Stelekati E, Benci JL, Xu B, Dada H, Odorizzi PM, Herati RS, Mansfield KD, Patsch D, Amaravadi RK, Schuchter LM, Ishwaran H, Mick R, Pryma DA, Xu X, Feldman MD, Gangadhar TC, Hahn SM, Wherry EJ, Vonderheide RH, Minn AJ. Radiation and dual checkpoint blockade activate non-redundant immune mechanisms in cancer. Nature. 2015;520(7547):373–7.

9. Golden Encouse. Abscopal responses in metastatic non-small cell lung cancer (NSCLC) patients treated on a phase II study of combined radiation therapy and ipilimumab: evidence for the in situ vaccination hypothesis of radiation. ASTRO 2015 Scientific Session T Lung II- Immunotherapy and Metastatic Disease Abstract 149.

10. Van Allen EM, Miao D, Schilling B, Shukla SA, Blank C, Zimmer L, Sucker A, Hillen U, Foppen MH, Goldinger SM, Utikal J, Hassel JC, Weide B, Kaehler KC, Loquai C, Mohr P, Gutzmer R, Dummer R, Gabriel S, Wu CJ, Schadendorf D, Garraway LA. Genomic correlates of response to CTLA-4 blockade in metastatic melanoma. Science 2015;350(6257):207–11.

11. Rizvi NA, Hellmann MD, Snyder A, Kvistborg P, Makarov V, Havel JJ, Lee W, Yuan J, Wong P, Ho TS, Miller ML, Rekhtman N, Moreira AL, Ibrahim F, Bruggeman C, Gasmi B, Zappasodi R, Maeda Y, Sander C, Garon EB, Merghoub T, Wolchok JD, Schumacher TN, Chan TA. Cancer immunology. Mutational landscape determines sensitivity to PD-1 blockade in non-small cell lung cancer. Science. 2015;348(6230):124–8.

12. Vanpouille-Box C, Diamond JM, Pilones KA, Zavadil J, Babb JS, Formenti SC, Barcellos-Hoff MH, Demaria S. TGFβ Is a Master Regulator of Radiation Therapy-Induced Antitumor Immunity. Cancer Res. 2015:75(11):2232–42.

13. Schumacher TN, Schreiber RD. Neoantigens in cancer immunotherapy. Science. 2015;348(6230):69–74.

14. Golden EB, Chhabra A, Chachoua A, Adams S, Donach M, Fenton-Kerimian M, Friedman K, Ponzo F, Babb JS, Goldberg J, Demaria S, Formenti SC. Local radiotherapy and granulocyte–macrophage colony-stimulating factor to generate abscopal responses in patients with metastatic solid tumors: a proof-of-principle trial. Lancet Oncol. 2015;16(7):795–803.

##### K20 Response and resistance to PD-1 pathway blockade: clues from the tumor microenvironment

###### Maria Libera Ascierto^1^, Tracee L. McMiller^2^, Alan E. Berger^3^, Ludmila Danilova^1,4^, Robert A. Anders^5^, George J. Netto^5^, Haiying Xu^7^, Theresa S. Pritchard^2^, Jinshui Fan^3^, Chris Cheadle^3^, Leslie Cope^1,4^, Charles G. Drake^1,6^, Drew M. Pardoll^1^, Janis M. Taube^5,7^ and Suzanne L. Topalian^2^

####### ^1^Department of Oncology, The Johns Hopkins University School of Medicine and Sidney Kimmel Comprehensive Cancer Center, Baltimore, MD, 21287, USA; ^2^Department of Surgery, The Johns Hopkins University School of Medicine and Sidney Kimmel Comprehensive Cancer Center, Baltimore, MD, 21287, USA; ^3^The Lowe Family Genomics Core, The Johns Hopkins University School of Medicine and Sidney Kimmel Comprehensive Cancer Center, Baltimore, MD, 21287, USA; ^4^Oncology Bioinformatics Core, The Johns Hopkins University School of Medicine and Sidney Kimmel Comprehensive Cancer Center, Baltimore, MD, 21287, USA; ^5^Department of Pathology, The Johns Hopkins University School of Medicine and Sidney Kimmel Comprehensive Cancer Center, Baltimore, MD, 21287, USA; ^6^The James Buchanan Brady Urological Institute, The Johns Hopkins University School of Medicine and Sidney Kimmel Comprehensive Cancer Center, Baltimore, MD 21287, USA; ^7^Department of Dermatology, The Johns Hopkins University School of Medicine and Sidney Kimmel Comprehensive Cancer Center, Baltimore, MD, 21287, USA

######## **Correspondence:** Maria Libera Ascierto

*Journal of Translational Medicine* 2016, **14(Suppl 1)**:K20

**Background:** Monoclonal antibodies blocking the PD-1 immune checkpoint have shown promising clinical results in multiple tumor types, including melanoma and renal cell carcinoma (RCC) [1]. We previously demonstrated that PD-L1 expression on tumor cells in pre-treatment biopsies is associated with favorable response to anti-PD-1 [2, 3]. In melanoma, comparing PD-L1+ vs. PD-L1(−) tumors, we found an overexpression of genes involved in CD8^+^ T-cell activation (e.g., *CD8A*, *IFNG*) and other immunosuppressive pathways (e.g., *LAG3* and *IL10*) in PD-L1+ tumors, suggesting the coordinate expression of multiple immunological factors that could influence response to anti-PD-1 therapy. The current study in RCC was undertaken to better understand why some patients with PD-L1+ tumors do not respond to anti-PD-1 treatment.

**Materials and methods:** Formalin-fixed, paraffin-embedded (FFPE) pre-treatment tumor biopsies expressing PD-L1 were derived from 13 RCC patients treated on four clinical trials of nivolumab (anti-PD-1) monotherapy at a single institution [4 responders (R), 9 non-responders (NR); RECIST]. PD-L1+ specimens were defined as those having ≥5 % of tumor cells with cell surface PD-L1 expression by immunohistochemistry (IHC) with the 5H1 mAb. RNA was isolated from PD-L1+ regions on FFPE slides and subjected to whole genome microarray profiling and multiplex quantitative (q)RT-PCR gene expression analysis.

**Results:** In RCC specimens, (q)RT-PCR and IHC assessment of expression of candidate immune genes previously found to be up-regulated in PD-L1+ vs. PD-L1(−) melanomas showed that none of these molecules was differentially expressed according to clinical outcome. Instead, unbiased whole genome expression analysis revealed that an up-regulation of genes associated with metabolic and solute transport functions was associated with treatment failure. Conversely, PD-L1+ RCCs from responding patients were found to overexpress immune markers such as BMP1, which has been shown to positively regulate PD-L1 expression, and CCL3 involved in leukocyte migration.

**Conclusions:** Metabolic factors are a hallmark of RCC and may be associated with resistance to anti-PD-1 therapy. Our findings suggest that the general approach used to identifying markers predicting clinical response to PD-1-targeted therapies, which has focused largely on immune modulatory receptors and ligands (e.g., PD-1, PD-L2, LAG-3) and T cell infiltrates, should be reevaluated. Indeed, a deeper level of investigation might be warranted for individual tumor types to which these new therapies are being applied.

**Clinical trials:** NCT00441337, NCT00730639, NCT01354431, NCT01358721

Supported by grants from Bristol-Myers Squibb, the National Cancer Institute, and a Stand Up To Cancer—Cancer Research Institute Cancer Immunology Translational Cancer Research Grant.

References

1. Topalian SL, Drake CG, Pardoll DM. Immune checkpoint blockade: a common denominator approach to cancer therapy. Cancer Cell. 2015;27(4):450–61.

2. Topalian SL, Hodi FS, Brahmer JR, Gettinger SN, Smith DC, McDermott DF, Powderly JD, Carvajal RD, Sosman JA, Atkins MB, Leming PD, Spigel DR, Antonia SJ, Horn L, Drake CG, Pardoll DM, Chen L, Sharfman WH, Anders RA, Taube JM, McMiller TL, Xu H, Korman AJ, Jure-Kunkel M, Agrawal S, McDonald D, Kollia GD, Gupta A, Wigginton JM, Sznol M. Safety, activity, and immune correlates of anti-PD-1 antibody in cancer. N Engl J Med. 2012;366(26):2443–54.

3. Taube JM, Klein A, Brahmer JR, Xu H, Pan X, Kim JH, Chen L, Pardoll DM, Topalian SL, Anders RA. Association of PD-1, PD-1 ligands, and other features of the tumor immune microenvironment with response to anti-PD-1 therapy. Clin Cancer Res. 2014;20(19):5064–74

##### K21 Combination immunotherapy with autologous stem cell transplantation, protein immunization, and PBMC reinfusion in myeloma patients

###### Sacha Gnjatic^1^, Sarah Nataraj^1^, Naoko Imai^1^, Adeeb Rahman^1^, Achim A. Jungbluth^2^, Linda Pan^3^, Ralph Venhaus^3^, Andrew Park^3^, Frédéric F. Lehmann^4^, Nikoletta Lendvai^2,*^, Adam D. Cohen^5,*^, and Hearn J. Cho^1,*^

####### ^1^Icahn School of Medicine at Mount Sinai, New York, USA; ^2^Memorial Sloan-Kettering Cancer Center, USA; ^3^Ludwig Institute for Cancer Research, New York, NY, USA; ^4^GSK Vaccines, Rixensart, Belgium; ^5^University of Pennsylvania, Philadelphia, PA, USA

######## **Correspondence:** Sacha Gnjatic

^*^Nikoletta Lendvai, Adam D. Cohen and Hearn J. Cho contributed equally to this work.

*Journal of Translational Medicine* 2016, **14(Suppl 1)**:K21

**Background:** Cancer vaccines, including those targeting cancer/testis antigens (CTA) such as MAGE-A3, are able to induce measurable adaptive immune responses, but have largely failed to demonstrate clinical benefit in phase III studies of solid tumors. Recent successes of adoptive cell therapy suggest that CTA-specific T cells may act better in the context of reinfusion after lympho/myeloablative therapy, due in part to immunologic benefits via lymphopenia-induced proliferation of T cells, elimination of suppressive populations, and possible release of tumor antigens for cross-priming in hematologic diseases. Therefore, we asked whether combining immunization with recMAGE-A3 + AS15 immunostimulant together with autologous stem-cell transplant (ASCT) and vaccine-primed lymphocyte reinfusion is safe and may lead to better immunogenicity in multiple myeloma (MM) patients.

**Materials and methods:** Thirteen MM patients who achieved at least very good partial response after induction therapy enrolled in a pilot study (NCT01380145) with one recMAGE-A3 + AS15 intramuscular immunization prior to ASCT followed by 7 booster immunizations shortly after ASCT and vaccine-primed lymphocyte reinfusion. Antibody (Ab) and cellular immune responses were assessed by ELISA, ELISpot, and intracellular cytokine staining (ICS) assays. Phenotypic changes in peripheral blood were assayed by mass cytometry. MAGE-A expression was assessed in MM cells by immunohistochemistry (IHC) using mAb M3H67.

**Results:** All patients had MAGE-A + MM cells at baseline. All patients developed MAGE-A3-specific Ab by ELISA and CD4 T cell responses by IFNγ ELISpot, with significant expansion after booster vaccinations that persisted through 1 year post-ASCT. ICS confirmed a polyfunctional, Th1-biased CD4 T cell response (IFNγ+, TNFα+, IL5−) in all patients. Three of 13 patients developed MAGE-A3-specific CD8 responses. Humoral antigen spreading was also observed. With a median follow-up of 31 mos, 9 patients have relapsed (median PFS is 27 months), one died of progressive MM, and one of subsequent allogeneic SCT complications. MAGE-A expression was assessed by IHC in seven relapse biopsies, and three were MAGE-A negative.

**Conclusions:** MAGE-A3 immunotherapy and PBL reconstitution induces strong Ab and Th1-biased CD4 T cell immune responses, and less frequently CD8 immune responses, in the setting of ASCT for MM. These Ab and CD4 T cell responses appear greater in magnitude than those seen historically in other cancers (PMID 14978137, 26309191), despite significant immune compromise after ASCT, highlighting the immunologic potency of this approach. Absence of MAGE-A3 expression in some relapsing patients points toward antigen-specific immune selective pressure and suggests that combination strategies aimed at limiting immune escape should be investigated.

**Funding sources:** GlaxoSmithKline Biologicals SA, Ludwig Institute for Cancer Research, Cancer Research Institute.

##### K22 Anti-cancer immunity despite T cell “exhaustion”

###### Speiser Daniel^1,2^

####### ^1^Department of Oncology and Ludwig Cancer Research Center, University of Lausanne, Lausanne, Switzerland; ^2^Campbell Family Institute, Princess Margaret Hospital, Toronto, Canada

######## **Correspondence:** Speiser Daniel

*Journal of Translational Medicine* 2016, **14(Suppl 1)**:K22

In cancers with increasing aggressiveness, cancer cells specialize for enhanced proliferation and cell survival. Simultaneously, they specialize on exploiting the host for supporting tumor growth. Consequently, many of the host’s reactions are contributing to the malignant behaviors of cancers. In turn, the immune system has a number of capabilities that have the potential of counteracting tumor growth and protecting the patient. Specifically, CD8+ cytotoxic T cells and CD4+ T helper one cells can destroy up to large tumor masses. More importantly, these anti-cancer T cells can provide long-term protection by keeping (minimal) residual disease in check, and patients alive for many years. However, in most patients T cells are dysfunctional and remain in a state of T cell “exhaustion”. Novel antibody therapies that block inhibitory receptors can lead to strong activation of anti-tumor T cells, mediating clinically significant anti-cancer immunity for many years. Therapeutic interventions are now being optimized for enforcing anti-cancer T cell functions, and avoiding immune inhibitory mechanisms of malignant diseases. Novel therapy modalities will likely become beneficial, provided that they help tipping the balance towards more activation and less inhibition of anti-cancer T cells. Carefully designed clinical studies with state-of-the art therapeutic and analytical techniques will provide detailed insight in the tumor microenvironment of patients, and the underlying mechanisms that cause tumor growth or regression. Such information is crucial for the design of promising large-scale (combination therapy) trials.

#### Immunotherapy in oncology (I-O): data from clinical trial

##### K23 The Checkpoint Inhibitors for the Treatment of Metastatic Non-small Cell Lung Cancer (NSCLC)

###### Vera Hirsh

####### McGill University, MUHC Royal Victoria Hospital, Montreal, QC, Canada

######## **Correspondence:** Vera Hirsh

*Journal of Translational Medicine* 2016, **14(Suppl 1)**:K23

Lung cancer is the leading cause of cancer-related deaths in men and the second leading cause in women world-wide^1^. Approximately 85 % of lung cancers are non-small cell lung cancers^2, 3^ and about 30 % of these, have a squamous histology which is associated with poorer outcomes and limited treatment options^4^.

The human immune system can be an effective way of fighting cancer. One mechanism the lymphatic checkpoint uses, to negatively regulate the early stages of T-cell activation, is through expression of the cytotoxic T-lymphocyte antigen-4 (CTLA-4) and antibody blockade of CTLA-4 has been shown to increase antitumor immunity^5^.

In peripheral tissues, the effector phase of the adaptive immune response against tumor cells is partly negatively regulated through the binding of the programmed cell death protein 1 (PD-1) expressed on activated T-cells with the ligands PD-L1 or PD-L2 expressed on tumor cells. Tumor cells can evade the immune response through the upregulation of PD-L1 in tumors or the tumor microenvironment^6^, leading to immune resistance.

CTLA-4 inhibitors re-activate T-cells by blocking the deactivating effects of CTLA-4. PD-1 and PD-L1 inhibitors re-activate T-cell activity in peripheral tissues by blocking the binding of PD-1 to its ligand PD-L1. Two CTLA-4 inhibitors, two PD-1 inhibitors, and four PD-L1 inhibitors are under development for NSCLC^7^.

Nivolumab recently received FDA approval for use as a single agent in second-line squamous NSCLC based on phase III trial data [8, 9]. The presentation will review available evidence for use of checkpoint inhibitors in advanced NSCLC.

References

1. Travis WD, Brambilla E, Riely GJ. New pathologic classification of lung cancer: relevance for clinical practice and clinical trials. J Clin Oncol. 2013;31:992–1001.

2. American Lung Association: Lung Cancer Fact Sheet [Web Page]. Available at: http://www.lung.org/lung-disease/lung-cancer/resources/facts-figures/lung-cancer-fact-sheet.html Accessed June 12 2015.

3. Herbst RS, Heymach JV, Lippman SM. Lung cancer. N Engl J Med. 2008;359:1367–80.

4. McKeage MJ, Jameson MB, Investigators ASSG. Comparative outcomes of squamous and non-squamous non-small cell lung cancer (NSCLC) patients in phase II studies of ASA404 (DMXAA)—retrospective analysis of pooled data. J Thorac Dis. 2010;2:199–204.

5. Leach DR, Krummel MF, Allison JP. Enhancement of antitumor immunity by CTLA-4 blockade. Science 1996;271:1734–36.

6. Pardoll DM. The blockade of immune checkpoints in cancer immunotherapy. Nat Rev Cancer 2012;12:252–64.

7. Pilotto S, Kinspergher S, Peretti U, et al. Immune checkpoint inhibitors for non-small cell lung cancer: does that represent a ‘new frontier’? Anticancer Agents Med Chem. 2014.

8. Gettinger SN HL, Gandhi L, et al. Overall survival and long-term safety of nivolumab (anti-PD-1 antibody, BMS-936558, ONO-4538) in patients with previously treated advanced non-small cell lung cancer. J Clin Oncol. 2015; April 20 (**Epub ahead of print**).

9. Brahmer JR, Horn L, Gandhi L, et al. Nivolumab (anti-PD-1, BMS-936558, ONO-4538) in patients (pts) with advanced non-small cell lung cancer (NSCLC): survival and clinical activity by subgroup analysis. ASCO Meeting Abstracts 2014;32:8112.

